# *De novo* transcriptomic analysis and development of EST-SSRs for *Sorbus pohuashanensis* (Hance) Hedl.

**DOI:** 10.1371/journal.pone.0179219

**Published:** 2017-06-14

**Authors:** Congcong Liu, Ying Dou, Xuelian Guan, Qiang Fu, Ze Zhang, Zenghui Hu, Jian Zheng, Yizeng Lu, Wei Li

**Affiliations:** 1 College of Landscape Architecture, Beijing University of Agriculture, Beijing, China; 2 Beijing Collaborative Innovation Center for Eco-environmental Improvement with Forestry and Fruit Trees, Beijing, China; 3 Beijing Engineering Research Center of rural landscape planning and design, Beijing, China; 4 Shandong Provincial Center of Forest Tree Germplasm Resources, Jinan, Shandong Province, China; 5 College of Landscape Architecture and Forestry, Qingdao Agricultural University, Qingdao, China; Youngstown State University, UNITED STATES

## Abstract

*Sorbus pohuashanensis* is a native tree species of northern China that is used for a variety of ecological purposes. The species is often grown as an ornamental landscape tree because of its beautiful form, silver flowers in early summer, attractive pinnate leaves in summer, and red leaves and fruits in autumn. However, development and further utilization of the species are hindered by the lack of comprehensive genetic information, which impedes research into its genetics and molecular biology. Recent advances in *de novo* transcriptome sequencing (RNA-seq) technology have provided an effective means to obtain genomic information from non-model species. Here, we applied RNA-seq for sequencing *S*. *pohuashanensis* leaves and obtained a total of 137,506 clean reads. After assembly, 96,213 unigenes with an average length of 770 bp were obtained. We found that 64.5% of the unigenes could be annotated using bioinformatics tools to analyze gene function and alignment with the NCBI database. Overall, 59,089 unigenes were annotated using the Nr database(non-redundant protein database), 35,225 unigenes were annotated using the GO (Gene Ontology categories) database, and 33,168 unigenes were annotated using COG (Cluster of Orthologous Groups). Analysis of the unigenes using the KEGG (Kyoto Encyclopedia of Genes and Genomes) database indicated that 13,953 unigenes were involved in 322 metabolic pathways. Finally, simple sequence repeat (SSR) site detection identified 6,604 unigenes that included EST-SSRs and a total of 7,473 EST-SSRs in the unigene sequences. Fifteen polymorphic SSRs were screened and found to be of use for future genetic research. These unigene sequences will provide important genetic resources for genetic improvement and investigation of biochemical processes in *S*. *pohuashanensis*.

## 1. Introduction

*Sorbus pohuashanensis* (Hance) Hedl. is a species of small deciduous tree in the genus *Sorbus* (subfamily Maloideae, family Rosaceae) that is native to China [1]. It has a high ornamental value due to the appearance of its leaves, flowers, and fruit in different seasons (S1 Fig). The trees also attract bird life, increase biodiversity, and provide appreciable ecological benefits [2]. With the increasing emphasis on city greening, greater attention is being paid to *S*. *pohuashanensis* trees because of their ornamental value and practical characteristics. However, limited bioinformatics information of *S*. *pohuashanensis* is available; previous studies on rowans mainly concentrated on the geographic distribution and habitats of wild plants [3], breeding strategies [4, 5], and population genetic diversity [6–8].Few molecular biology studies have been reported.

Here, we studied the transcriptome in the leaves of *S*. *pohuashanensis* trees. The transcriptome is the set of all RNA molecules in one cell or a population of cells, and includes both mRNAs and non-coding RNAs. The development of next generation sequencing of the transcriptome (RNA-seq) has provided the advantages of high accuracy and throughput, great sensitivity, and low operating costs. Not only can *de novo* sequencing of the transcriptome provide genetic information of a species without the aid of a reference genome, but also can forecast probable non-coding RNAs [9]. RNA-seq has gradually replaced gene chip technology and become the preferred method for genomic study of gene expression in plants [10]. RNA-seq has been used to analyze the transcriptomes of *Chimonanthus praecox* [11], *Eucommia ulmoides* [12], *Platycladus orientalis* [13], *Myrica rubra* [14], *Salix* spp. [15], and *Syringa oblata* [16]. Here, we used RNA-seq to construct a transcriptome database for *S*. *pohuashanensis*. This information will be of value for future development and genetic modification of the germplasm of this species.

Simple sequence repeats (SSRs) are one of the most efficient genetic markers. By virtue of their reproducibility, multi-allelic nature, co-dominant inheritance, relative abundance, and good genome coverage, SSR markers have been widely applied in genetic diversity studies [17]. According to their locations in the genome, SSR markers are generally divided into genomic SSRs and EST-SSRs (expressed sequence tag SSRs). In comparison with genomic SSR markers, EST-SSR markers are derived from coding regions and are believed to have some practical advantages, such as low cost and relative ease of identification, applicability to assays of functional diversity in natural populations or germplasm collections, high transferability to related species, and utility as anchor markers for comparative mapping or evolutionary studies [18].

However, the development of EST-SSRs can be difficult because of the laborious methods required and high cost of identifying ESTs. As a consequence, EST-SSR marker development in *S*. *pohuashanensis* has not been reported. With the development of next-generation sequencing technology, creating transcriptome-level sequence collections has become much quicker and cheaper. As a result, several gene-based SSRs and other genetic markers depending on such resources have been identified and developed in various plant species such as *Amentotaxus spp*. [19], *Dipteronia spp*. [20], *Taxodium* ‘zhongshansa’ [21], *E*. *ulmoides* [12], *Pinus koraiensis* [22], and *Cunninghamia lanceolata* [23]; however, to date, they have not been reported in *S*. *pohuashanensis*. Therefore, The transcriptome database offers an attractive alternative to complement existing SSR collections. In this study, we reported the first development of EST-SSR markers in *S*. *pohuashanensis*, which could provide new opportunities for assessing molecular phylogeny and genetic diversity in this species and other species in the genus *Sorbus*.

## 2. Materials and methods

### 2.1 Plant materials

Five *S*. *pohuashanensis* plants with strong growth and no indication of pests or disease infections were selected from the forest germplasm resources nursery of Beijing University of Agriculture, of the National Forest Genetic Resources Platform (NFGR). During the summer, three leaves were picked from each of the five trees (biological replicates) and separately frozen in liquid nitrogen.

Eight 2-year-old *S*. *pohuashanensis* plants from Laoshan provenance were randomly chosen from the forest germplasm resources nursery of Beijing University of Agriculture, of the National Forest Genetic Resources Platform (NFGR) and used in the development of the EST-SSR markers in this study.

### 2.2 DNA extraction

Genomic DNAs were extracted from leaves of the2-year-old plants using a DNA extraction kit [Model: tgDP320-03, Tiangen Biotech (Beijing) CO. LTD] following the manufacturer’s protocol. DNA quality and quantity were checked in 1% agarose gels and Eppendorf BioSpectrometer (Eppendorf, Germany), respectively.

### 2.3 RNA extraction and purification

Total RNA was extracted using an RNAqueousTotal RNA Isolation kit (Ambion) following the manufacturer’s instructions. The integrity of the extracted RNA was checked using an Agilent Bioanalyzer 2100 (Agilent Technologies, Santa Clara, CA, US). Total RNA was further purified using an RNeasyMicro kit (Qiagen, GmBH, Germany) and RNase-Free DNase Set (Qiagen).

### 2.4 Sequencing library construction

mRNA was isolated from the purified total RNA and fragmented. The following steps were performed as described in the Illumina workflow protocol: synthesis of first and second strand cDNAs, tail end repair, addition of A to the 3′-end, joint bonding, and enrichment [24]. Five cDNA libraries were constructed, and their concentrations and sizes were checked individually using a Qubit2.0 Fluorometer and Agilent 2100 Fluorometer. Cluster generation and first dimension primer hybridization were performed using an IlluminacBot matched to llluminaHiSeq, and paired end (2×125 nt multiplex) sequencing was performed with the paired-end program. Sequencing was performed by the Shanghai Biotechnology Corporation.

### 2.5 Sequence data analysis and transcript assembly

To obtain clean reads, the Fastax online software (version: 0.0.13, http://hannonlab.cshl.edu/fastx_toolkit/index.html) was used to screen out unqualified reads from the raw reads; this step removed joint sequences, reads with low overall quality (mainly reads with lengths less than 20bp), reads with base N(base with uncertain identity), reads with more than 20% of the bases possessing Q-values ≤10, and or low end quality. Clean reads from the five libraries were de novo assembled using CLC Genomics Workbench (version:6.0.4) according to the scaffolding contig algorithm (word-size = 45, minimum contig length = 300) [25–27]. These various steps produced the primary unigenes. These were then assembled for a second time using CAP3 EST software [28] to acquire the final unigene sequence set. This unigene set was used for further exploration of the transcriptome.

### 2.6 Sequence annotation and functional classification

The final unigene set was compared against the NCBI non-redundant (Nr) database and Uniprot database using BLASTX [29], with an E-value<1e^-5^. Gene function was annotated through these comparisons. Unigene sequences annotated to the Nr database were then compared against GO (Gene Ontology) [30] using BLASTX (E-value<1e^-5^). GO function classification was obtained for the top five alignment results against the processes “molecular function”, “cellular component” and “biological process”. The annotated unigenes were compared against the CDD database [31] with an E-value ≤ 1e^-5^. COG function prediction [32] was performed on the top five results, which were classified and then mapped on each level of COG. Finally, a mapping analysis using KEGG (Kyoto Encyclopedia of Genes and Genomes) [33] was performed using the online pathway alignment analysis tool KEGG KASS.

### 2.7 Identification of EST-SSRs loci

The unigene sequences were analyzed for SSRs using MISA software [34]. SSR loci were identified using the search criteria that the minimum repetitions of di-, tri-, tetra-, penta-, and hexa-nucleotides were 6, 5, 4, 4, and 4, respectively, and the flanking sequence length of the SSR loci was greater than 50 bp. As it is difficult to authenticate the presence and stability of single nucleotide repeats and compound nucleotide repeats, these two structures were not analyzed in this study.

### 2.8 Primer screening and designing of EST-SSR markers

Primer pairs flanking the SSRs were designed using Primer3 online design software (http://www.simgene.com/Primer3) following the core criteria: the optimum length of the primers was 20 bp, ranging from 18 to 22 bp; the annealing temperature was between 55°C and 62°C; the GC content ranged from 40–60%; and the optimum size of the PCR products was 100–300 bp. A total of 140 EST-SSRprimer pairs were designed with the criteria mentioned above (S1 File). To confirm their utility, all of the designed primers were screened using a sample from one 2-year-old plant, which was chosen at random, by 2% agarose gel electrophoresis. Then, primers producing a single clear band were screened on samples from four other plants by 3% agarose gel electrophoresis. Finally, the primers producing a single clear band in all the four samples were screened on samples from eight plants using an ABI 3730XL capillary electrophoresis analyzer, and the polymorphic SSR loci were identified.

#### Polymerase chain reaction (PCR)and polymorphic marker validation

Genotyping PCR amplifications were performed in 25 μL reaction volumes as follows: 12.5 μL of 2×Taq MasterMix (Ruibiotech, Beijing, China), approximately 50 ng of DNA, 5 pmol forward primer with the 5′end labeled with a fluorescent dye (FAM; Ruibiotech, Beijing, China), 5 pmol reverse primer, and sterile double-distilled water added to 25 μL. The amplifications were performed using the following schedule: denaturation at 94°C for 5 min; 30 cycles of denaturation for 30 s at 94°C, annealing for 30 s at the optimal temperature, and then extension for 30 s at 72°C; a final extension at 72°C for 7 min. All the PCR amplifications were performed using the same thermal cycler (Applied Biosystems, Foster, CA, USA). The PCR products were separated by an ABI 3730XLcapillary electrophoresis analyzer (Applied Biosystems, Foster, CA, USA) with a GeneScan-500LIZ size standard. Fragments were genotyped for their presence/absence at each locus, and the allele sizes were scored using GeneMaker 2.2.0 software (Soft Genetics LIC, State College, PA, USA) and visually checked twice to reduce genotyping errors.

## 3. Results

### 3.1 *De novo* assembly

The preliminary assembly yielded 137,506 contigs, with an average length of 588 bp and an N50 value of 650 (Table 1). Using the CLC Genomics Workbench, 115,987 primary unigenes with an average length of 703 bp (N50 value: 768) were obtained. The primary unigenes were assembled using CAP3 EST software to produce a final unigene set of 96,213 unigenes with an average length of 700 bp and N50 value of 894.

**Table 1:** Summary of assembly statistics for *Sorbus pohuashanensis* leaf transcriptome.

Statistics	counts	Total length (bp)	N50 (bp)	Average length (bp)	longest (bp)	N%	GC%
Contigs	137,506	80,881,146	650	588	12,084	0	42.0
Primary Unigenes	115,987	81,529,176	768	703	13,674	0.8	42.0
Final Unigenes	96,213	74,058,783	894	770	13,675	0.87	42.0

**Fig 1:**
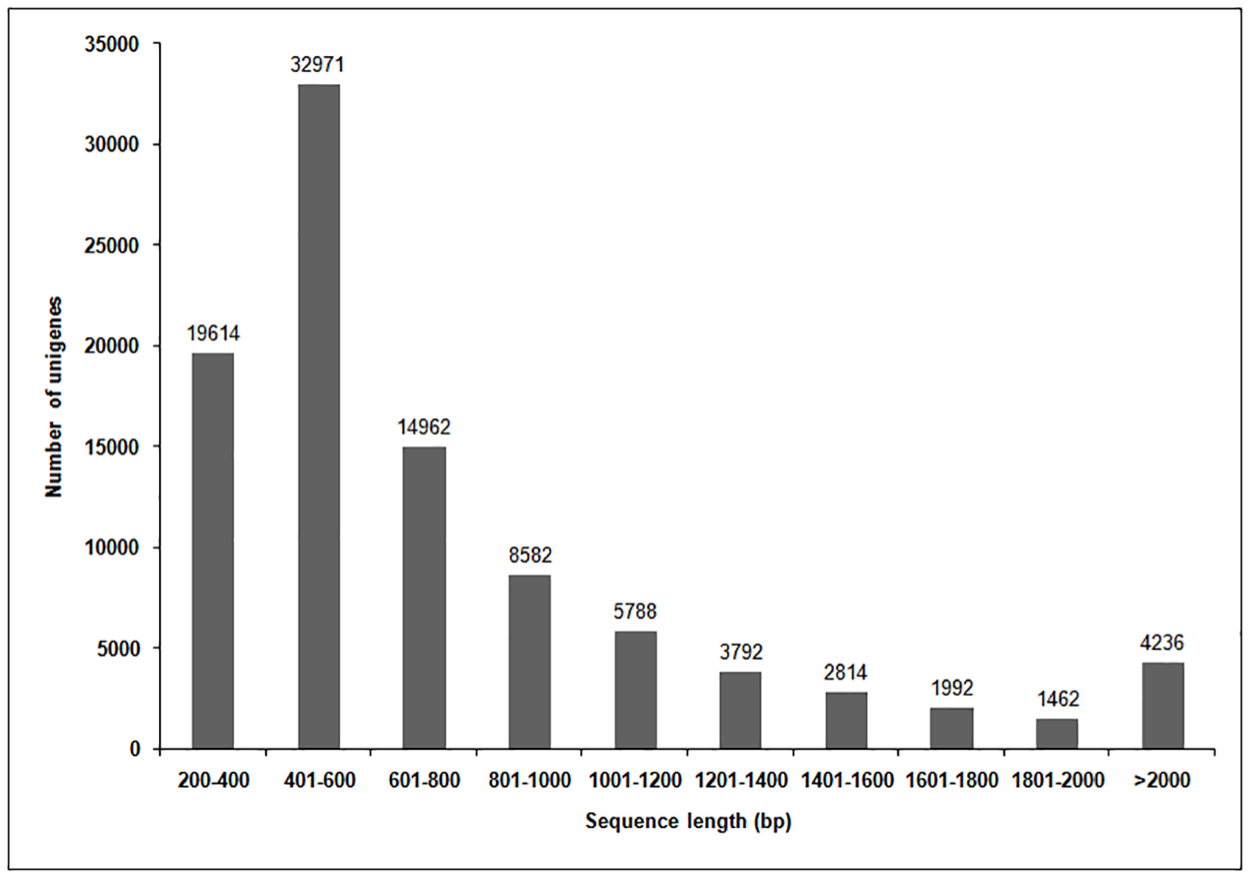
Length distribution of assembled *Sorbus pohuashanensis* leaf unigenes. Clean reads for each *S*. *pohuashanensis* leaf were combined and resulted in 96,213 unigenes. Horizontal and vertical axes show the size and number of unigenes, respectively.

The final unigene set contained 19,614 sequences (20.39%), with lengths less than 400 bp; 32,971 unigenes (34.27%), with lengths ranging from 401 to 600 bp; 14,962 unigenes (15.55%), with lengths ranging from 601 to 800 bp;8,582 unigenes (8.91%), with lengths ranging from 801 to 1000 bp; and 20,084 unigenes (20.87%), with lengths above 1000 bp (Fig 1).

### 3.2 Gene function annotation

**Fig 2:**
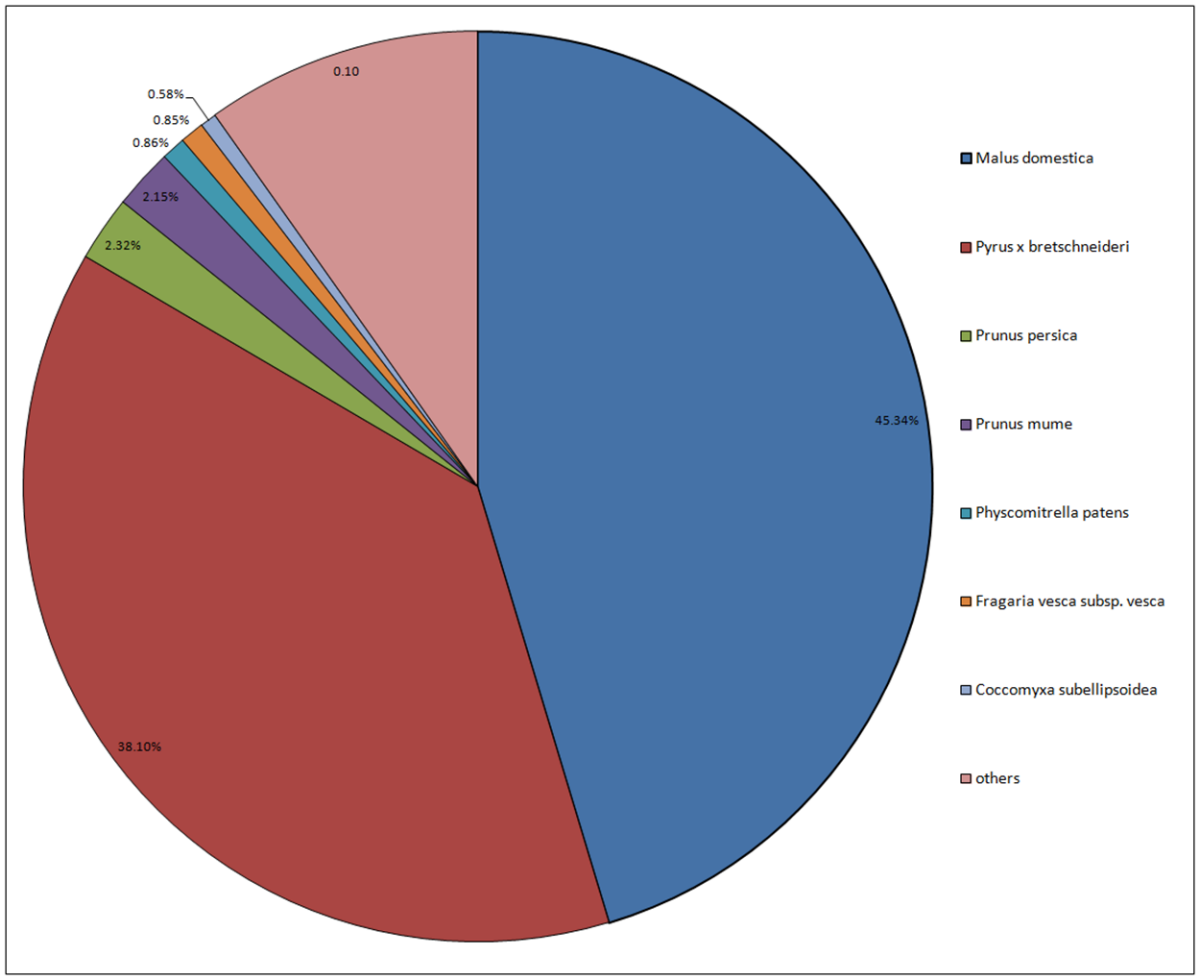
Species-based distribution of BLASTX matches for unigenes against NCBI Nr database. We used all plant proteins in the NCBI Nr database in performing the homology search; for each unigene, we selected the closest match for analysis.

The final unigene set was searched against the Nr, Uniprot, GO, COG and KEGG databases and a total of 62,053 unigenes were annotated (Table 2). From the species statistics on unigenes that were annotated in the Nr database, we found that 26,788 genes (45.34%) were annotated to *Malus domestica*, 22,512 genes (38.10%) to *Pyrus* × *bretschneideri*, 1,369 (2.32%) to *Pyrus persica*, and 1,270 (2.15%) to *P*. *mume*. This suggests that *S*. *pohuashanensis*is is closely related to *M*. *domestica* and *Pyrus* × *bretschneideri*, and less closely related to *P*. *persica* and *P*. *mume*. The details of the comparison are shown in Fig 2 and S2 File.

**Table 2:** Statistics of annotation results for *Sorbus pohuashanensis* unigenes.

Database	NR	SwissProt	GO	COG	KEGG	All
Number annotated	59,089	55,978	35,225	33,168	13,953	62,053

### 3.3 GO classification for unigenes

A search of the GO database using BLAST2GO software and the final unigene set generated 35,225 unigenes that annotated to the GO database. GO classification was carried out on the annotated unigenes which showed they mainly involved three biological functions: 28,895 (82.03%) unigenes annotated to “biological process” (GO: 0008150); 12,495 unigenes (35.47%) annotated to “cellular component” (GO: 0005575); and 31,424 (89.21%) annotated to “molecular function” (GO: 0003674). The GO database annotation indicated that the 35,225 unigenes annotated to 2,939 GO terms; biological process included 2,888 GO terms and was the largest group (Table 3, S2 Fig).

**Table 3:** Statistics of GO functional annotation results for *Sorbus pohuashanensis* leaf unigenes.

GO Type	Number of Unigenes annotated	Number of GO term
Biological process	28895	2888
Cellular component	12495	519
Molecular function	31424	1669
All	35225	2939

**Fig 3:**
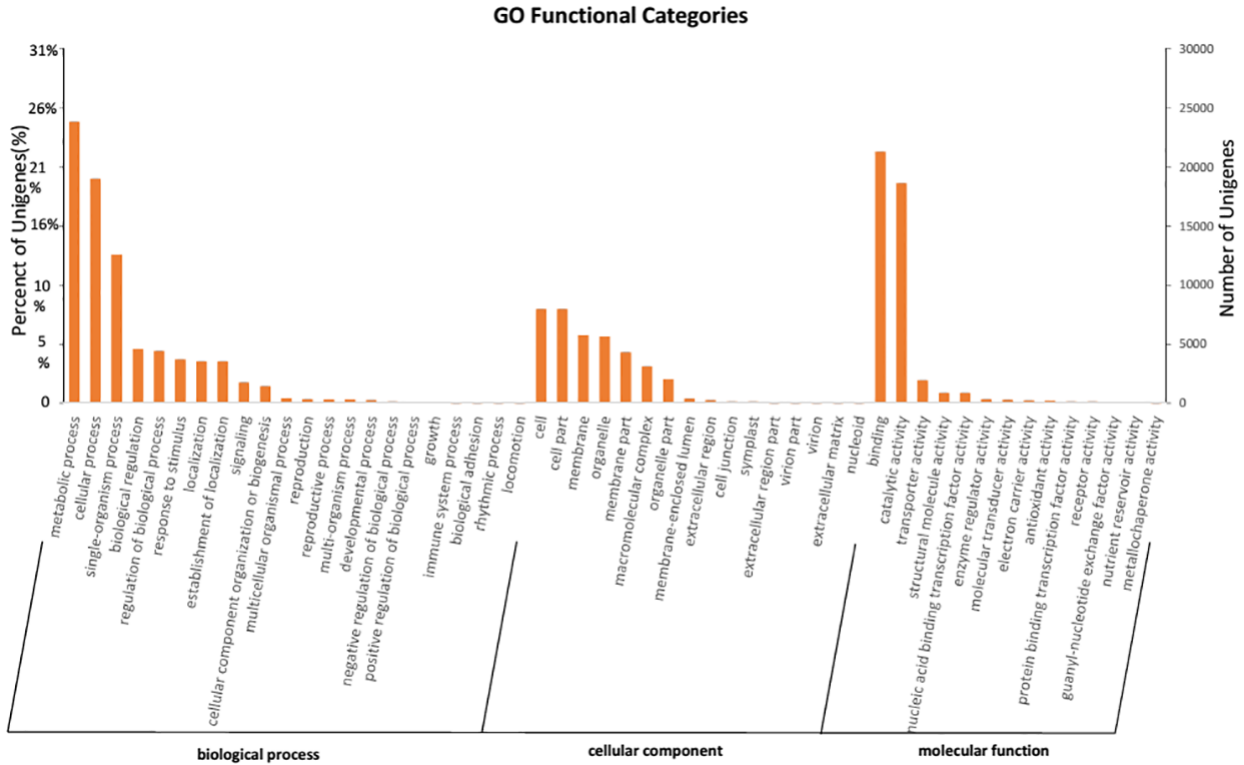
Gene Ontology (GO) classification of *Sorbus pohuashanensis* leaf unigenes. Left y-axis indicates the percentage of unigenes in subcategories of each main category. Right y-axis indicates the number of unigenes in each subcategory.

Unigenes in the three biological function categories were divided into 52 biological function subgroups using GO function classification. In the biological process category, 23,859 (67.73%) were assigned to the metabolic process subgroup, 19,036 (54.04%) to the cellular process subgroup, and 12,604 (35.78%) to single-organism process genes.In the cellular component category, 8,001 (22.71%) were assigned to the cell and cell part expression, 5,772 (16.39%) to the membrane, 5,669 (16.09%) to the organelle. In the molecular function category, 21,323 (60.53%) were assigned to the binding function, 18,652 (52.95%) to the catalytic activity (Fig 3, S3 File).

### 3.4 Protein classification and function predictions

**Fig 4:**
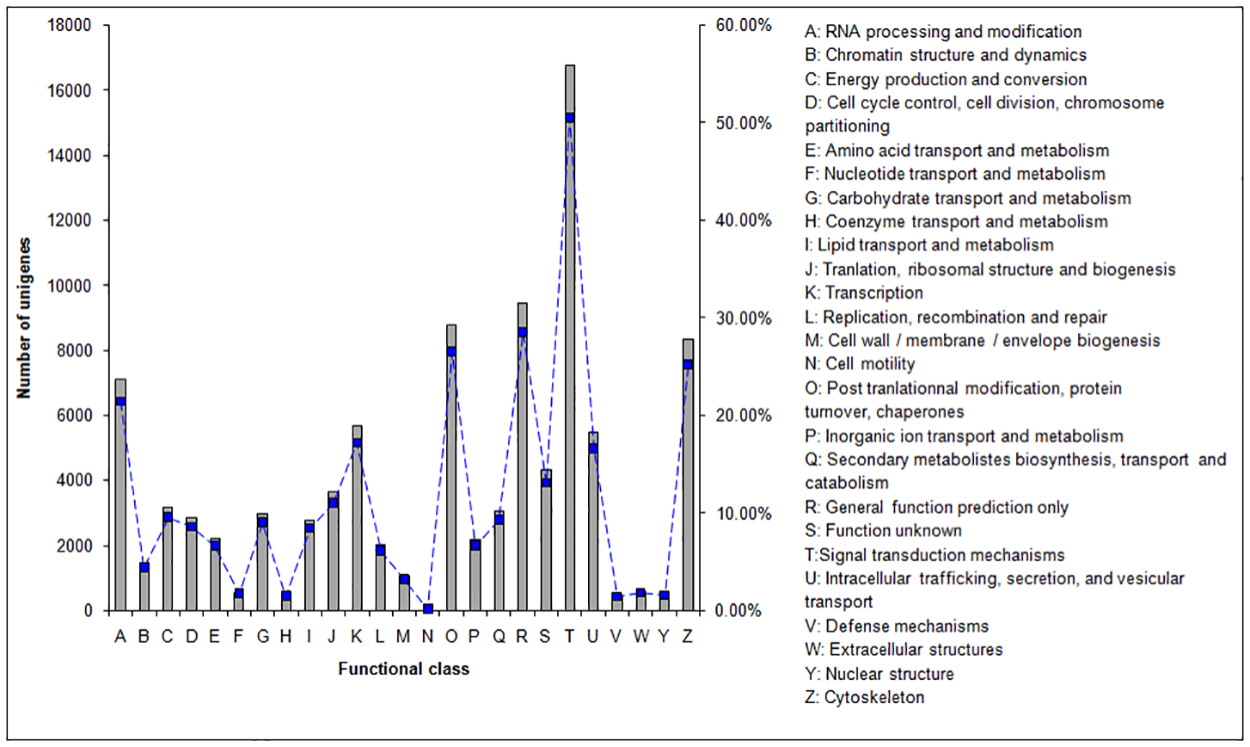
COG functional classification of *Sorbus pohuashanensis* unigenes. From the final set of 96,213 unigenes, 33,225 were annotated with significant homology in the COG database (E-value≤1.0 E^–5^) and were classified into 25 KOG categories.

The Clusters of Orthologous Groups (COG) of proteins includes data from bacteria, algae, and eukaryotes and is based on phylogenetic relationships. The final unigene set obtained here was searched against the COG database (E-value ≤ 1e^-5^), and prediction of COG function classification was performed on annotated unigenes. Of the 96,213 unigenes in the final set, 33,168 were annotated to the COG database and assigned to 25 function classifications (Table 3, Fig 4). The largest group of unigenes (16,748 members, 50.49%) was assigned to the T class (signal transduction mechanisms); R class (general function prediction) accounted for 9,449 unigenes (28.49%); O class (posttranslational modification, protein turnover, chaperones) accounted for 8,780 (26.47%); N class (cell motility) was the smallest, with only 22 unigenes (Fig 4, S4 File). We also found that 441 unigenes (1.33% of total) were involved in V class (defense mechanisms) (Fig 4). The identification of genes participating in signal transduction and defense mechanisms will be of value for future studies on abiotic stress mechanisms in *S*. *pohuashanensis*. Isolation and identification of related genes will also be of importance for future work.

### 3.5 Metabolic pathway analysis of unigenes

KEGG enables assignment of unigenes to intracellular metabolic pathways; thus, the function of gene products can be analyzed systematically and complex biological behaviors can be explored. We performed a KEGG mapping analysis of the final unigenes set and found that 13,953 unigenes were assigned to 322 metabolic pathways (S5 File). Of these 13,953 unigenes, 980 (7.02%) were involved in metabolic pathways with large numbers of genes; 435 (3.12%) were involved in biosynthesis of secondary metabolites 217 (1.56%) were involved in biosynthesis of antibiotics and 179 (1.28%) in microbial metabolism in diverse environments. Only a few unigenes (0.3%) were involved in the signaling pathway, such as AMPK, MAPK, and P53. Undoubtedly, these data will be useful for future metabolic research in *S*. *pohuashanensis*.

### 3.6 EST-SSR marker analysis of *S*. *pohuashanensis*

**Fig 5:**
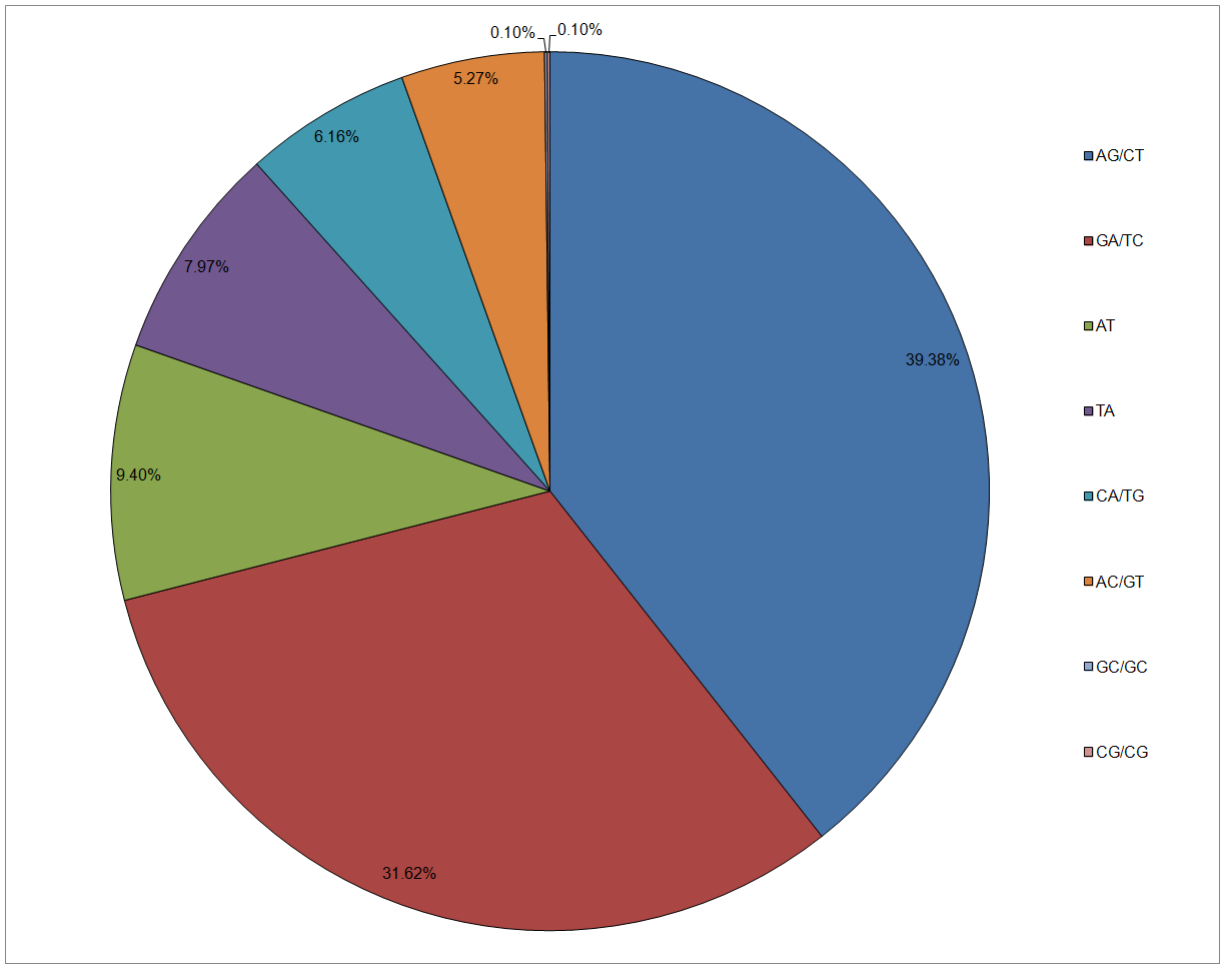
Percentage of different motifs in di-nucleotide repeats.

We identified 7,473 SSR sites in 6,604 unigenes (7.7%) of the annotated 96,213 unigenes (Table 4). Among these 6,604 unigenes, one SSR site was present on average every 9.1 kb sequence; 5,839 unigenes contained only one site, while 765 unigenes contained two or more sites (Table 4, S6 File).Di-nucleotide repeats accounted for the majority (66.49%)of the identified SSRs; tri-nucleotide repeats accounted for 31.02% of the total, whereas, tetra-nucleotide, penta-nucleotide and hexa-nucleotide repeats were rare and accounted for 2.49% (Table 4). The frequencies of AG/CT and GA/TC were highest in di-nucleotide repeats, accounting for 39.98% and 31.62% of di-nucleotide SSRs, respectively. AT and TA repeats accounted for 9.40% and 7.97% of di-nucleotide SSRs respectively. We also found a few CG/GC repeats (0.2%) in the di-nucleotide repeats (Fig 5).We identified 583 different repeat unit types in the 7,473 SSR sites; overall, we found 179 di-nucleotide, 262 tri-nucleotide, 92 tetra-nucleotide, 32 penta-nucleotide and 18 hexa-nucleotide type repeats (S5 File).

**Table 4:** Distribution of the SSR motifs in the *Sorbus pohuashanensis* transcriptome.

SSR type	SSR Number	Unigenes number	Occurrence frequency (%)	Percent (%)	Average Distributing distance (per SSR/kb)	Average length (bp)
Di-nucleotide	4969	4387	5.16%	66.49%	14.90	17
Tri-nucleotide	2318	2048	2.41%	31.02%	31.95	15
Tetra-nucleotide	132	119	0.14%	1.77%	561.05	21
Penta-nucleotide	35	32	0.04%	0.47%	2115.97	24
Hexa-nucleotide	19	18	0.02%	0.25%	3897.83	29
total	7473	6604	7.77%	100.00%	9.91	21

### 3.8 Validation of EST-SSRs

We selected 24 from the 140 SSR primer pairs that provided a clear and consistent amplification band after PCR amplification (S1 File). Using an ABI3700 DNA analyzer, the selected 24 pairs of primers (the forward primer was 5′end labeled with FAM fluorescent dye) were tested and analyzed; 15 SSRs were found to be polymorphic. The primer sequence, motif repeats, and allele number of each SSR marker are given in Table 5, and the genotype profiles of the eight 2-year-old plants are shown in the S7 File.

**Table 5:** SSR primer sequences and repeat types.

Primer NO.	Primier sequence (5'-3')		Repeat motif	Number of allele	Tm/°C
	F	R			
sorsb3	TGCATGGTCGGAGAACATAA	TACGAGTCCAACCTCCCAAC	(AAG)10	3,166\172\184	52
sorsb9	GGATAGGAGCACGACTCTGG	GCCCAGCTATCCATTTTCAA	(AAG)7	2,199\214	52
sorsc3	TTCTCGGCACTTGCTGTATG	CGAGGAACCAGAGGAGAAGA	(CTT)10	3,151\154\160	52
sorse2	GAACTTGGGCAGGTAAGCAC	CTCGGTCCCTGAATTGTGTT	(TC)10	6,237\239\241\243\245\251	52
sorse5	TATGGTTGATCGGCTTTGGT	GGTTGCACTCGAATCATCAA	(TC)10	3,206\230\240	52
sorse10	TTTCCTCCCCACATCAAAAG	AAGGCAACTTGTTGGGTACG	(TC)10	5,238\240\242\246\250	52
sorse17	GCCTTGGACTTTAGAGCTTGC	ACTCCAGCCTTTCTCGGATT	(TC)10	6,240\242\248\250\252\256	52
sorse19	CGGGATTATCTTCCCAACAA	TGTCGGAAACAGGATTGTCA	(TC)10	3,116\122\126	52
sorse50	TATGGTTGATCGGCTTTGGT	TCCTTCTTGGTTGCACTTGA	(TC)12	3,212\240\250	52
sorsg1	TCTCTCACTCCTCTCCCCAAT	GGATCTGAACCCATTGAAGC	(GAA)12	2,220\223	52
sorsg8	CAGCAGATCTCTCGGCTCTC	TCATCACTCGCGACTACCTG	(GAA)8	3,172\175\178	58
sorsh13	TCCGAATGCAGTGAAGAAGA	ATGGATGACGGATTGCTCTC	(GAG)6	3,162\165\168	52
sorsj14	GCGGAAACTTCTTCCGTGTA	TGGCGTTACAAATGGTTTGA	(TCC)6	3,251\254\266	52
sorsj16	TCTCTCCGCATTCTCCTTGT	GGGGAAAAGAGAGAGGGCTA	(TCC)6	3,144\150\156	52
sorsk10	TTTGAGGCCATTGAGTGTTG	TGGTGTTTGCGAGTTTTCTG	(TTC)6	2,242\251	52

## 4. Discussion

### 4.1 Transcriptome sequencing, assembly, and functional annotation of unigenes

*S*. *pohuashanensis* is a native tree species in China with considerable ornamental value in urban landscapes. However, the dearth of information on its genetics has limited breeding and exploitation of the species. Here, we used an RNA-seq approach to sequence the transcriptome of leaves and used these sequences to identity and characterize the genes and proteins of the *S*. *pohuashanensis* leaves. The average length of the unigenes obtained here (770 bp) was greater than reported for *P*. *orientalis* (475 bp) [13], *M*. *rubra* (531 bp) [14], bamboo (736 bp) [35], willow (652 bp), and *Salix suchowensis* (723bp) [15], although less than for *S*. *oblata* (853 bp) [16] and *C*. *praecox* (1190 bp) [11]. A total of 62,053 unigenes were identified in this study; 35.50% of these had no homologs in the NCBI database. However, the annotated unigenes showed greater similarity to sequences from *M*. *domestica* and *Pyrus* × *bretschneideri* (homology matching rate of 83.44%) than to other species. This result is in good agreement with taxonomic studies and confirms that *S*. *pohuashanensis*is more closely related to *M*. *domestica* and *Pyrus* × *bretschneideri* [1].

Annotation of the unigenes showed that the largest group annotated to the molecular function group (89.21%) (Table 2, S2 Fig). This result is different to those reported for *S*. *oblata* and *C*. *praecox*, probably because of use of different tissue samples; the transcriptomes analyzed in *S*. *oblata* and *C*. *praecox* were obtained from flowers [11, 16]. Our KEGG function annotation analysis showed that 13,953 unigenes participated in 322 metabolic pathways. The largest number of unigenes were involved in metabolic pathways, while those involved in biosynthesis of secondary metabolites were next most frequent. These data will be of value for future metabolic research in *S*. *pohuashanensis*. Overall, our sequencing of the *S*. *pohuashanensis* transcriptome has provided considerable information that will be of use to future studies on a range of practical applications of this species.

### 4.2 EST-SSR markers analysis of *S*. *pohuashanensis*

Based on their high rate of polymorphism, good reproducibility, and codominance, SSRs have been widely used for plant DNA fingerprinting, analysis of genetic diversity, gene mapping, and molecular marker-assisted breeding. However, the development of SSR marker primers is essential for their use [36, 37]. Simple sequence repeat markers for expressed sequence tags (ESTs) identify coding sequences and can be used to directly obtain information on gene expression as they are closely linked to functional genes. Additionally, the primers for SSRs have high transferability among related species. A large number of EST sequences have been identified from transcriptome data using high-throughput sequencing [38]. To improve research into genetic diversity and marker assisted selection, we can use transcriptome sequences to develop EST-SSR markers. In this study, we found 7,473 SSR sites in 6,604 of the 96,213 annotated unigenes, i.e., 7.77% of the unigenes, contained SSR sites (Table 4). This rate of SSR sites is higher than in *E*. *ulmoides* (6.54%) [12], *Ginkgo biloba* (5.97%) [39], *S*. *suchowensis* (5.4%) [15], and *Jatropha carcas* (6.51%) [40]. We also found that 5,839 unigenes contained only one SSR site, while 765 contained two or more sites (S6 File). On average, there was one SSR site every 9.1 kb DNA sequence (Table 4). These results suggested that the frequency of EST-SSRs in *S*. *pohuashanensis* was higher than *E*. *ulmoides* (one every 11.61 kb) [12], *Populus* spp. (one every 14 kb), and *G*. *biloba* (one every 12.02 kb) [39], but lower than *Camellia sinensis* (one every 3.68 kb) [41], *Liriodindron tulipifera* (one every 8.5 kb) [42], and *Heveabra siliensis* (one every 3.93 kb) [43].

Di-nucleotide repeats were the most common type (Table 4). The frequencies of AG/CT and GA/TC di-nucleotides (39.98% and 31.62%, respectively), were similar to those reported for *E*. *ulmoides* [12], *Salvia splendens* [44], *Saccharum officinarum* [45], and *Sorghum bicolor* [46]. Simultaneously, we also found a few CG/GC repeats, accounting for 0.20% of di-nucleotide SSRs (Fig 5). In addition, CTT/GAA, AAG/TTC, CTC/GAG, AGA/TCT, CCT/GGA, AGG/TCC, CCA/GGT, AAC/TTG, CAC/GTG, and ACC/TCC sequences accounted for 59.71% of the total tri-nucleotides, and were mainly tri-nucleotide repeat motif types (S6 File). This result was similar to *G*. *biloba* [39], *C*. *sinensis* [41], and *L*. *tulipifera* [42]. We believe that our analysis provides a solid foundation for the development and application of *S*. *pohuashanensis* EST-SSRs.

Finally, we designed a total of 140 pairs of EST-SSR primers and validated their accuracy using samples from eight 2-year-old *S*. *pohuashanensis* plants. We found that 130 primer pairs produced amplification products (data not shown), i.e., an effective amplification rate of 92.86%.This rate is higher than that reported for walnut (90.9%) [47], pear (64.6%) [48], and apple (63.9%) [49]. Some primer pairs did not amplify products, probably because the EST-SSR location of the primers contained introns in the genome sequences. However, only 15 EST-SSR primer pairs were polymorphic, accounting for 11.54% of primer pairs that successfully amplified products. This rate is significantly lower than in apples[49], pears[48], *Amentotaxus spp*. [19], and *C*. *lanceolata*[23]. The polymorphism levels might be related to quantity differences in the sample materials. The polymorphism results indicate that it will be feasible to use the 15 EST-SSRs for genetic research on *S*. *pohuashanensis* in the future.

## 5. Conclusions

We used RNA-seq to perform *de novo* transcriptome sequencing of *S*. *pohuashanensis*. Additionally, we also obtained information on EST-SSRs. To the best of our knowledge, this is the first comprehensive report on an *S*. *pohuashanensis* transcriptome. The unigenes were assembled without use of a reference genome. More than 80 Gbp raw reads and 96,213 unigenes (total length 74.06 Gbp) were acquired by splice and assembly; 62,053 unigenes were annotated in the Nr, Uniprot, GO, COG, and KEGG databases. In addition, we identified 7,743 EST-SSRs and discovered 15 EST-SSRs with polymorphism through experimental verification. These findings will provide a valuable information resource for use in research on genetic improvement, genetic engineering, and other biochemical studies in *S*. *pohuashanensis*.

## Supporting information

S1 Fig Appearance of *Sorbus pohuashanensis*. a: Spring; b: Summer; c: early Autumn; d: middle or late Autumn.
(TIF)

S2 Fig Distribution of GO functional level. (TIF)

S1 File The repeat motif types and the primer sequences of the 140 SSRs for *Sorbus pohuashanensis*. (XLSX)

S2 File Species-based distribution of BLASTX matches for unigenes against NCBI Nr database. (XLSX)

S3 File Summary of GO term assignment for the *Sorbus pohuashanensis* leaf transcriptome. (Level2).
(XLSX)

S4 File COG annotation of *Sorbus pohuashanensis* leaf unigenes. (XLSX)

S5 File Summary of KEGG pathways involved in the *Sorbus pohuashanensis* leaf transcriptome. (XLSX)

S6 File Summary of the simple sequence repeats (SSRs) of *Sorbus pohuashanensis*. (XLSX)

S7 File Genotype profiles of 15 SSR loci in eight 2-year-old *Sorbus pohuashanensis* plants. (PDF)
