# Supplementary material for: De novo transcriptomic analysis and development of EST-SSRs for Sorbus pohuashanensis (Hance) Hedl
Source: PLoS One. 2017 Jun 14;12(6):e0179219. doi: 10.1371/journal.pone.0179219 (PMC5470691; doi:10.1371/journal.pone.0179219)
Supplement: S7 File — (PDF) [file pone.0179219.s009.pdf]

Sample 1: sorsb3-01.A01.fsa Run date and time: 03/19/2017 - 18:17:56 -> 03/19/2017 - 19:01:55

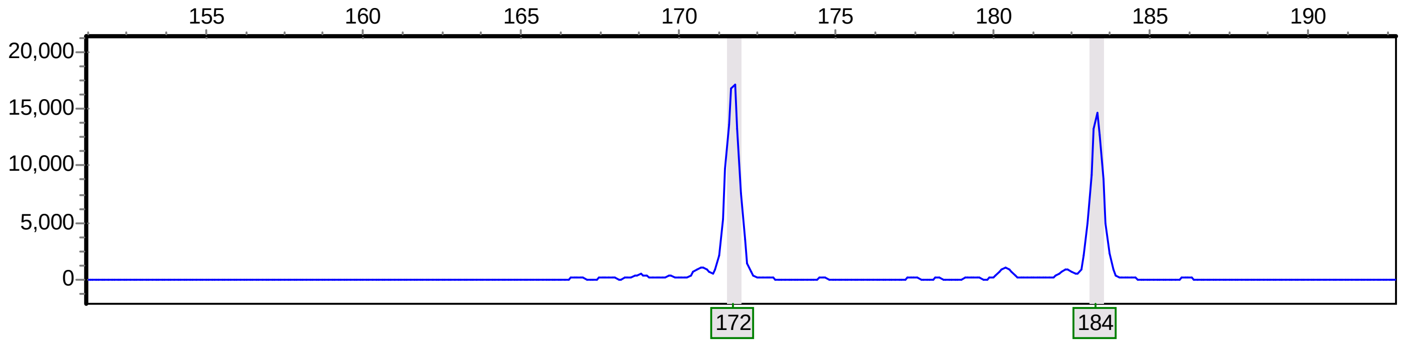

Sample 2 sorsb3-04.B01.fsa Run date and time: 03/19/2017 - 18:17:56 -> 03/19/2017 - 19:01:55

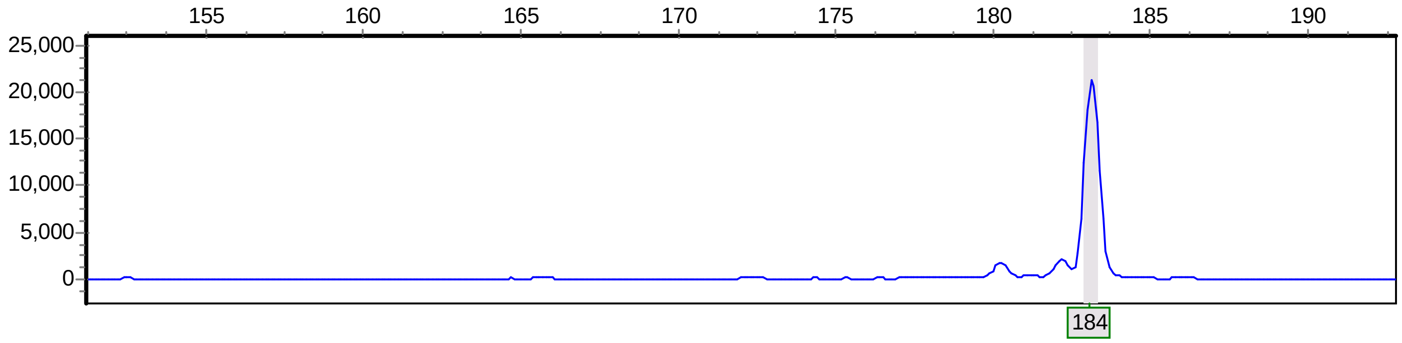

Sample 3 sorsb3-05.A01.fsa Run date and time: 03/21/2017 - 13:32:38 -> 03/21/2017 - 14:23:07

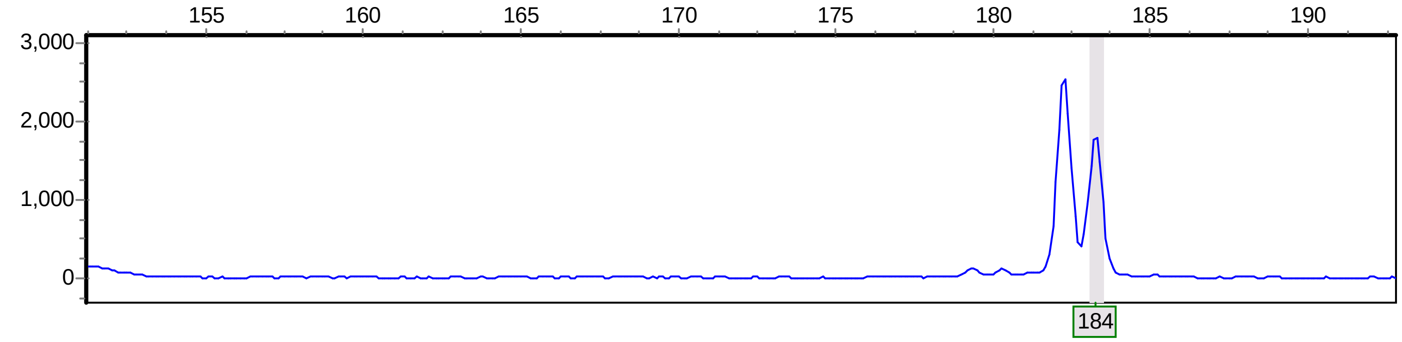

Sample 4 sorsb3-06.B01.fsa Run date and time: 03/21/2017 - 13:32:38 -> 03/21/2017 - 14:23:07

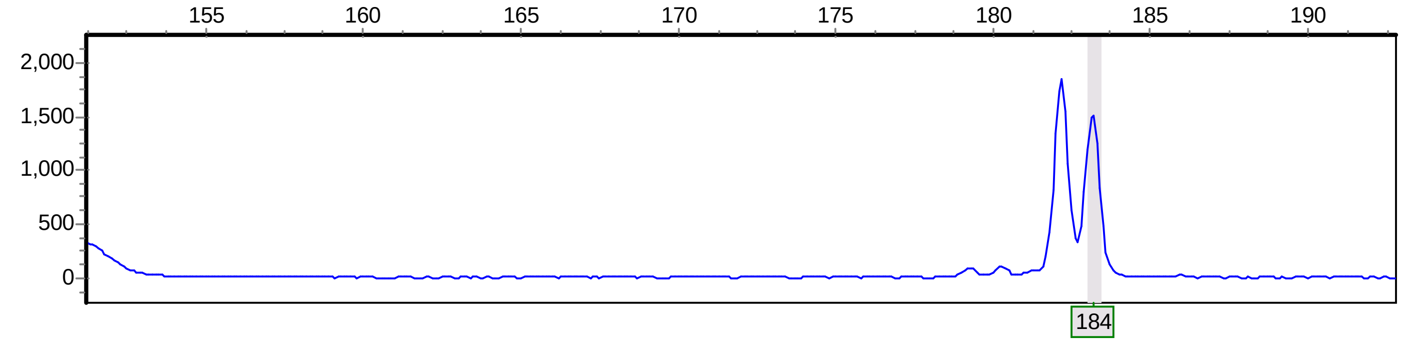

PDF 檔案使用 "pdfFactory Pro" 試用版本建立 www.pdffactory.com

Sample 5: sorsb3-07.C01.fsa Run date and time: 03/21/2017 - 13:32:38 -> 03/21/2017 - 14:23:07

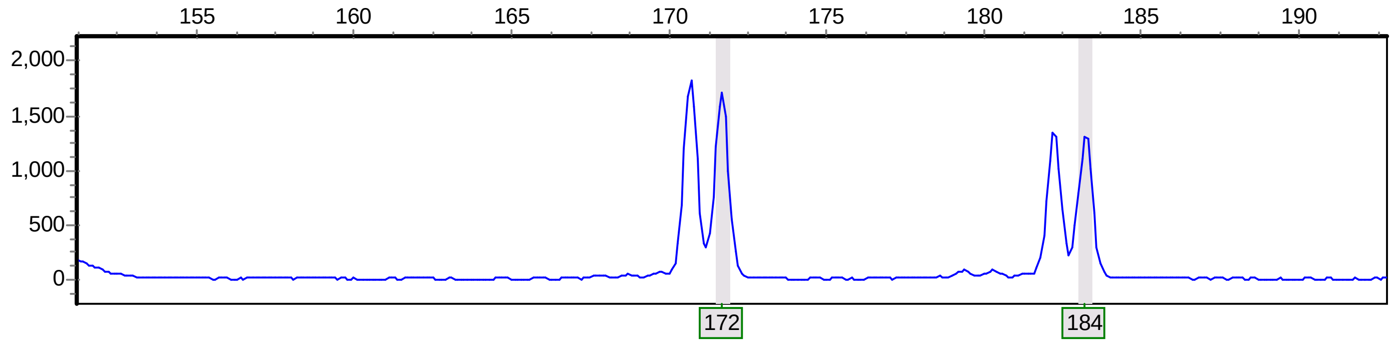

Sample 6 sorsb3-08-1.E01.fsa Run date and time: 03/20/2017 - 21:37:15 -> 03/20/2017 - 22:21:17

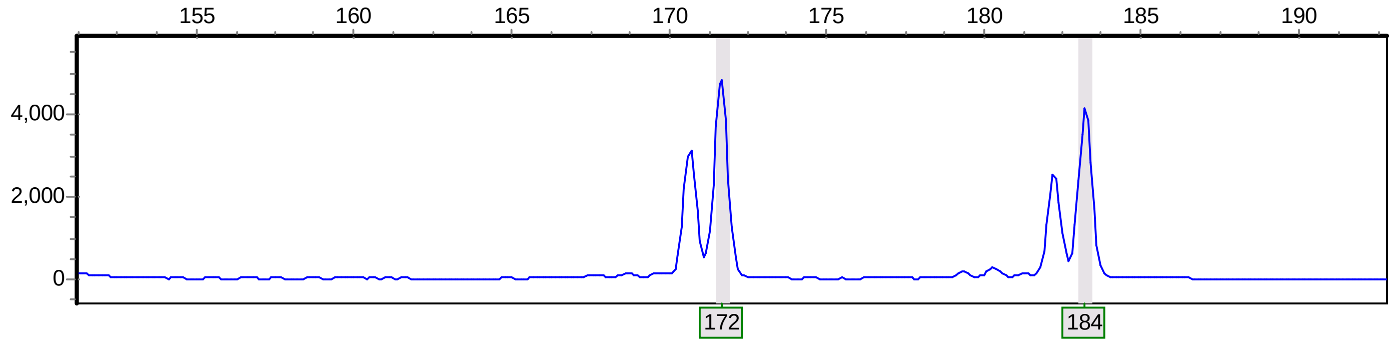

Sample 7 sorsb3-08.D01.fsa Run date and time: 03/20/2017 - 21:37:15 -> 03/20/2017 - 22:21:17

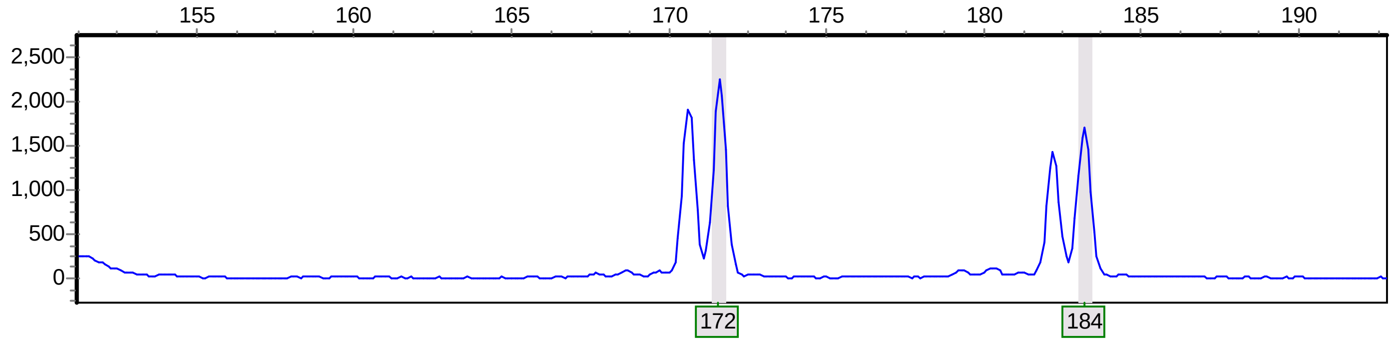

Sample 8 sorsb3-09.F01.fsa Run date and time: 03/20/2017 - 21:37:15 -> 03/20/2017 - 22:21:17

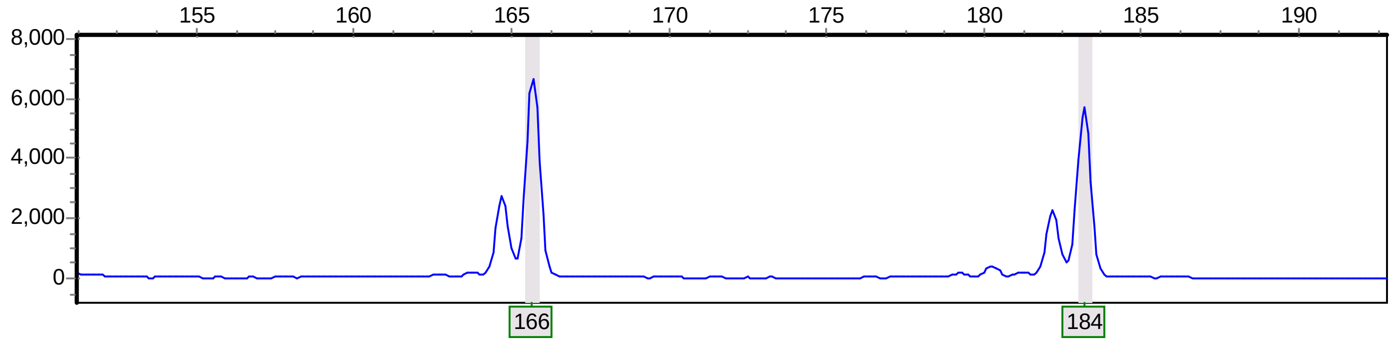

PDF 檔案使用 "pdfFactory Pro" 試用版本建立 www.pdffactory.com

Sample 1: sorsb9-01.C01.fsa Run date and time: 03/19/2017 - 18:17:56 -> 03/19/2017 - 19:01:55

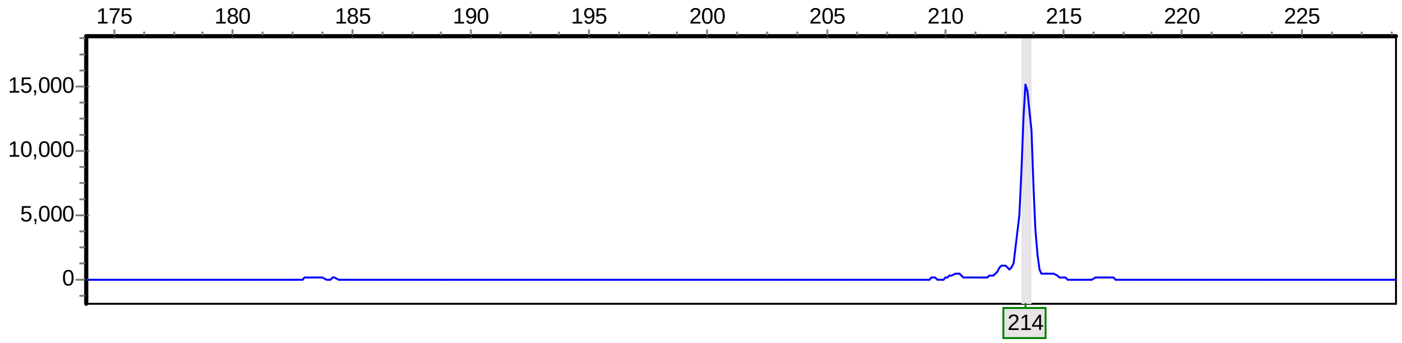

Sample 2 sorsb9-04.D01.fsa Run date and time: 03/21/2017 - 13:32:38 -> 03/21/2017 - 14:23:07

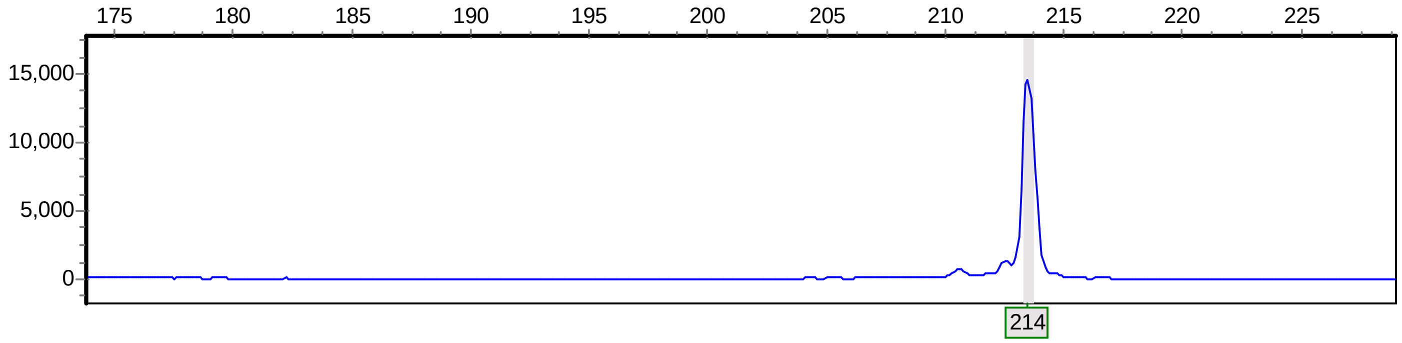

Sample 3 sorsb9-05.A02.fsa Run date and time: 03/20/2017 - 21:37:15 -> 03/20/2017 - 22:21:17

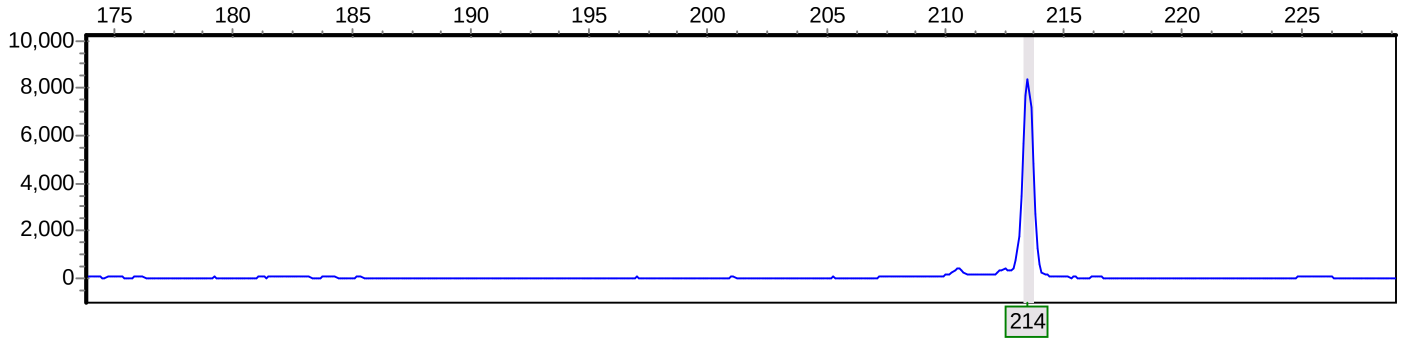

Sample 4 sorsb9-06.B02.fsa Run date and time: 03/20/2017 - 21:37:15 -> 03/20/2017 - 22:21:17

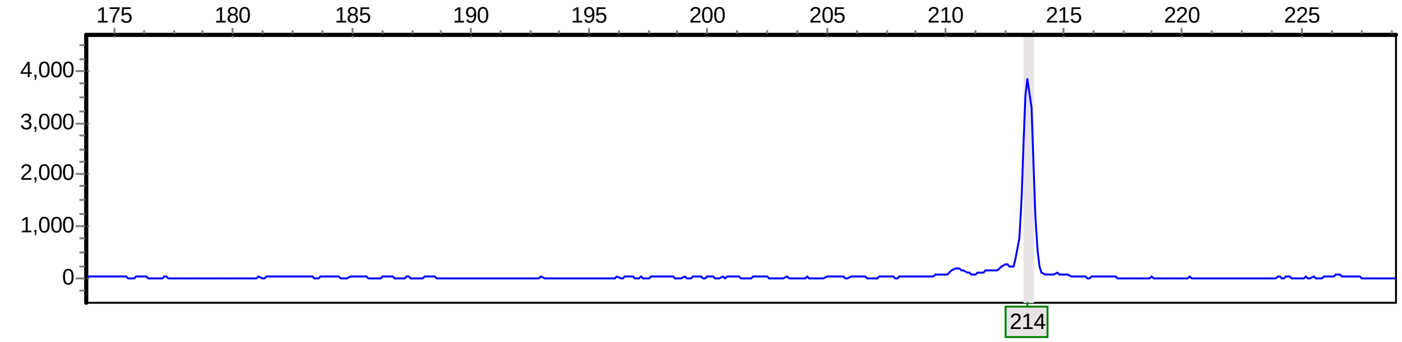

PDF 檔案使用 "pdfFactory Pro" 試用版本建立 www.pdffactory.com

Allele Report

Sample 5: sorsb9-07.C02.fsa Run date and time: 03/20/2017 - 21:37:15 -> 03/20/2017 - 22:21:17

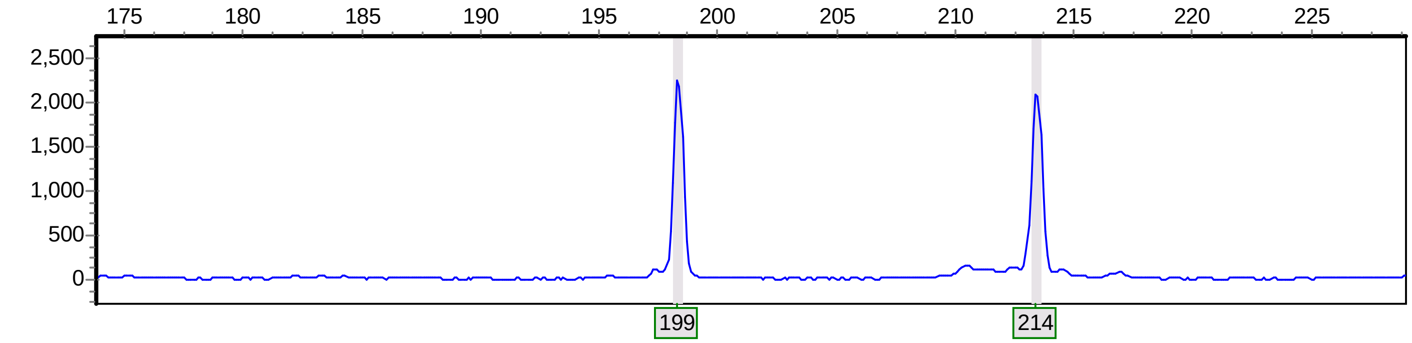

Sample 6 sorsb9-08-1.E02.fsa Run date and time: 03/20/2017 - 21:37:15 -> 03/20/2017 - 22:21:17

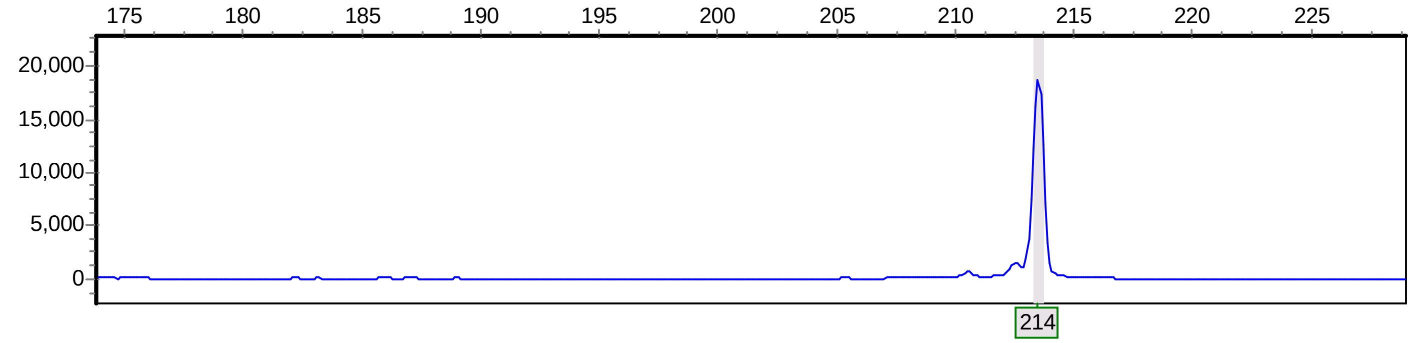

Sample 7 sorsb9-08.D02.fsa Run date and time: 03/20/2017 - 21:37:15 -> 03/20/2017 - 22:21:17

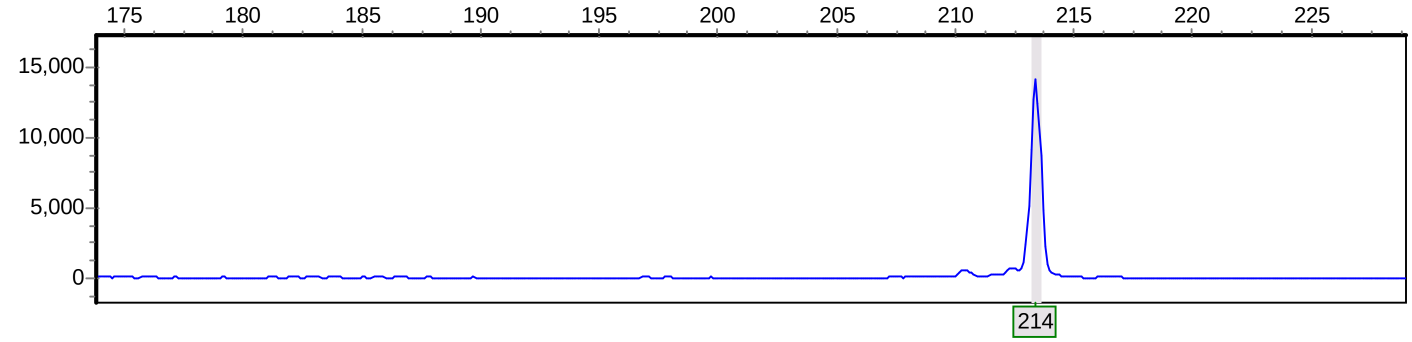

Sample 8 sorsb9-09.F02.fsa Run date and time: 03/20/2017 - 21:37:15 -> 03/20/2017 - 22:21:17

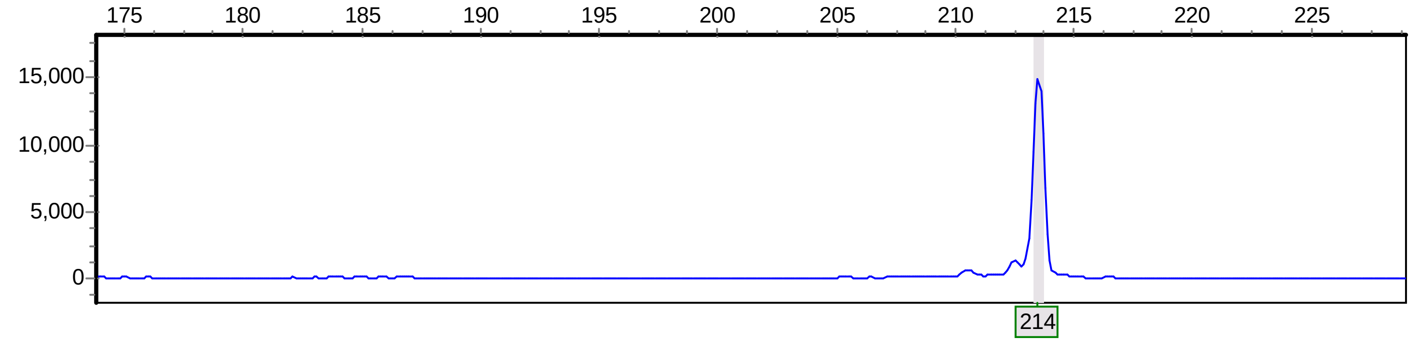

PDF 檔案使用 "pdfFactory Pro" 試用版本建立 www.pdffactory.com

SoftGenetics
GeneMarker V2.2.0

Allele Report

2017-4-13 18:02:39

Sample 1: sorsc3-01.G01.fsa Run date and time: 03/19/2017 - 18:17:56 -> 03/19/2017 - 19:01:55

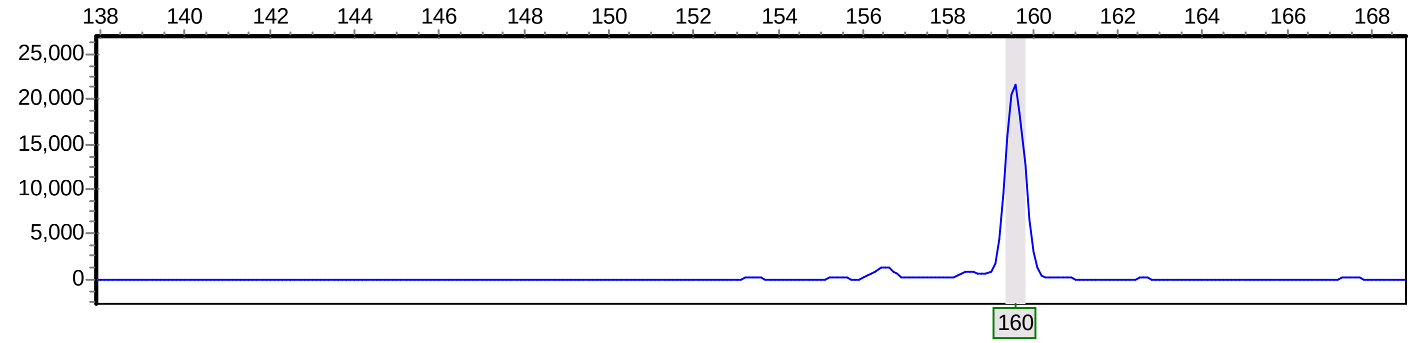

Sample 2 sorsc3-04.H01.fsa Run date and time: 03/19/2017 - 18:17:56 -> 03/19/2017 - 19:01:55

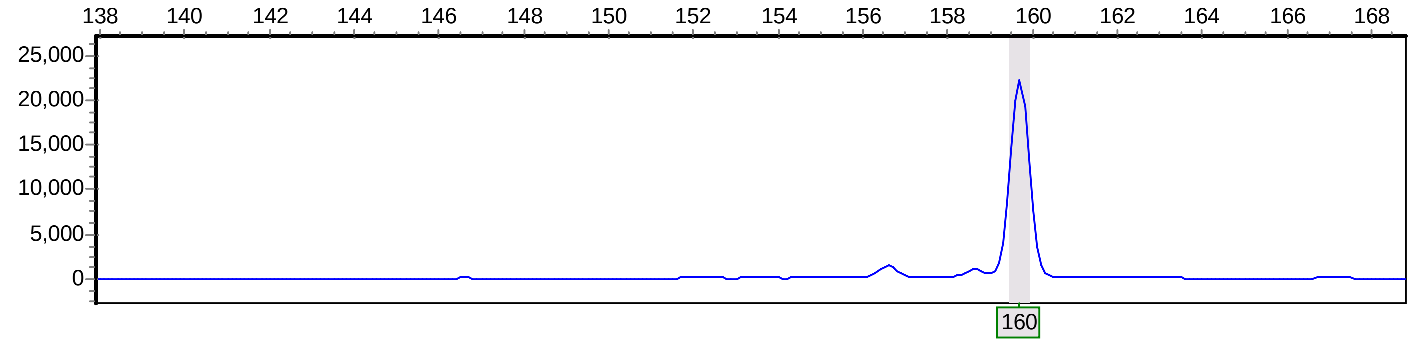

Sample 3 sorsc3-05.A04.fsa Run date and time: 03/20/2017 - 21:37:15 -> 03/20/2017 - 22:21:17

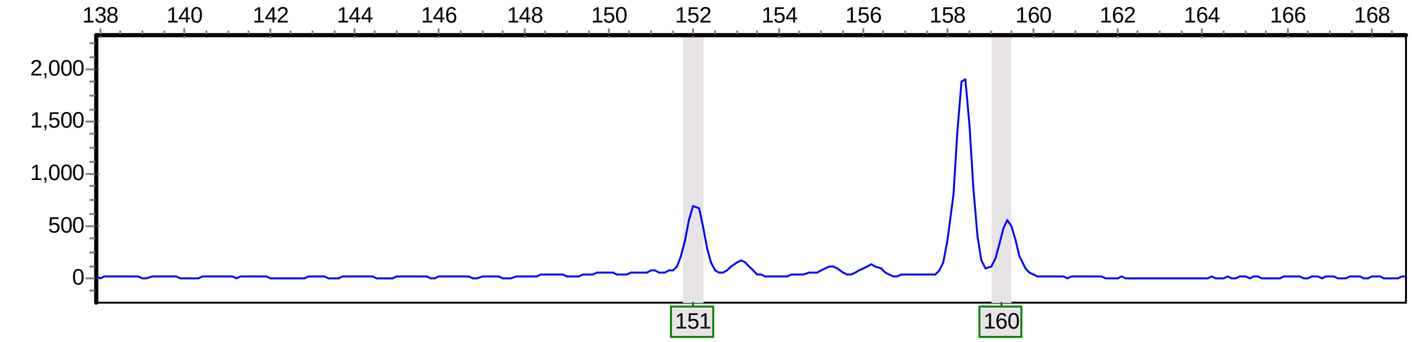

Sample 4 sorsc3-06.B04.fsa Run date and time: 03/21/2017 - 13:32:38 -> 03/21/2017 - 14:23:07

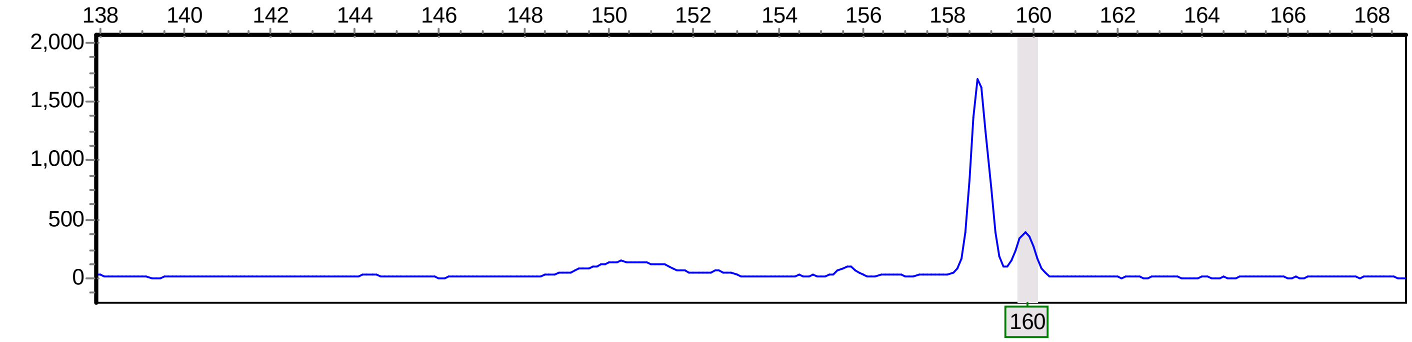

PDF 檔案使用 "pdfFactory Pro" 試用版本建立 www.pdffactory.com

Allele Report

Sample 5: sorsc3-07.C04.fsa Run date and time: 03/20/2017 - 21:37:15 -> 03/20/2017 - 22:21:17

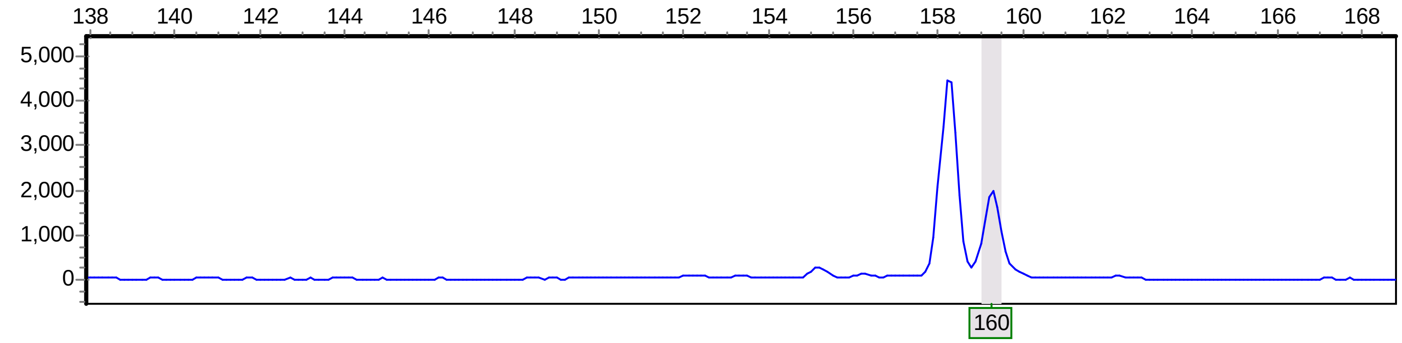

Sample 6 sorsc3-08-1.E04.fsa Run date and time: 03/20/2017 - 21:37:15 -> 03/20/2017 - 22:21:17

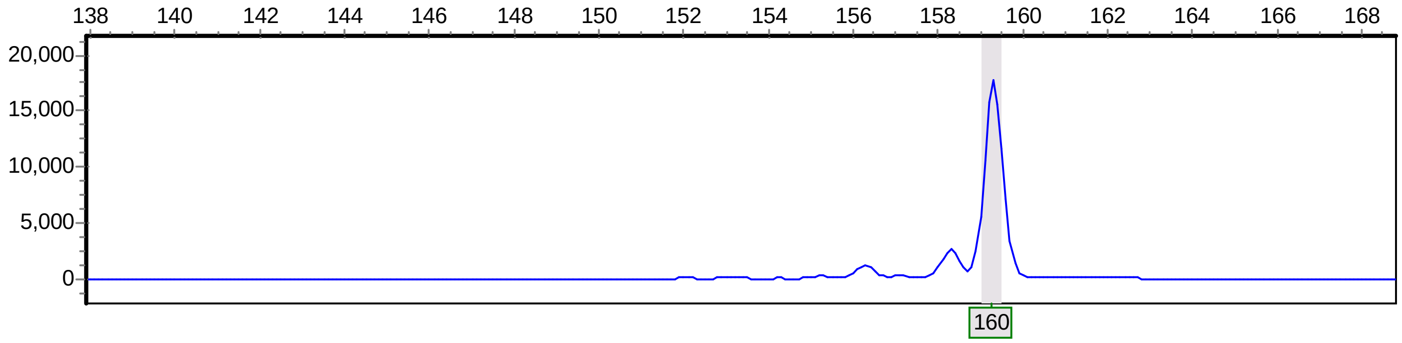

Sample 7 sorsc3-08.D04.fsa Run date and time: 03/20/2017 - 21:37:15 -> 03/20/2017 - 22:21:17

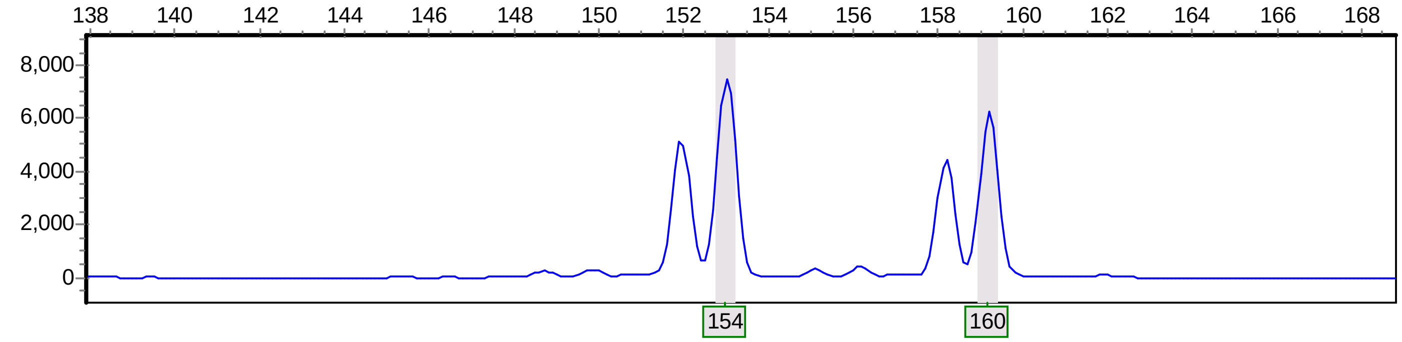

Sample 8 sorsc3-09.F04.fsa Run date and time: 03/20/2017 - 21:37:15 -> 03/20/2017 - 22:21:17

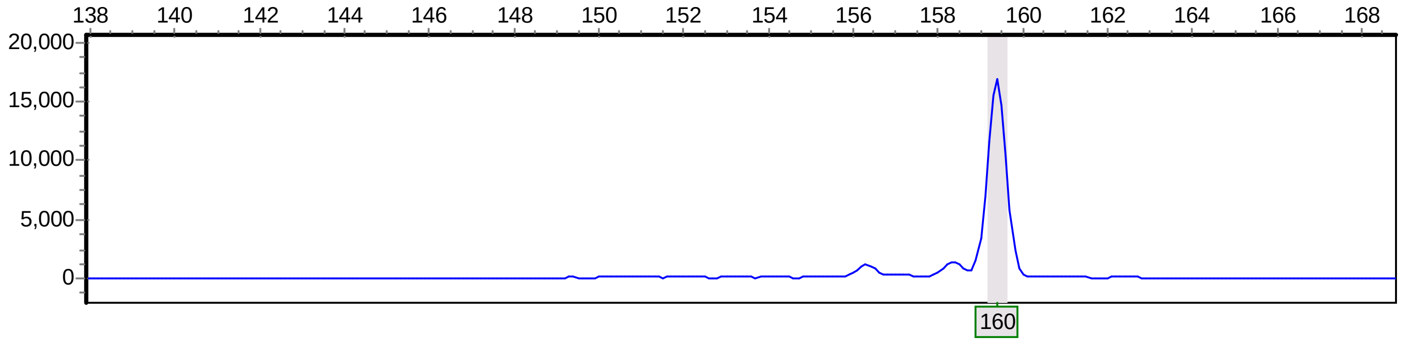

PDF 檔案使用 "pdfFactory Pro" 試用版本建立 www.pdffactory.com

SoftGenetics

GeneMarker V2.2.0

Allele Report

Sample 1: sorse2-01.A02.fsa Run date and time: 03/19/2017 - 18:17:56 -> 03/19/2017 - 19:01:55

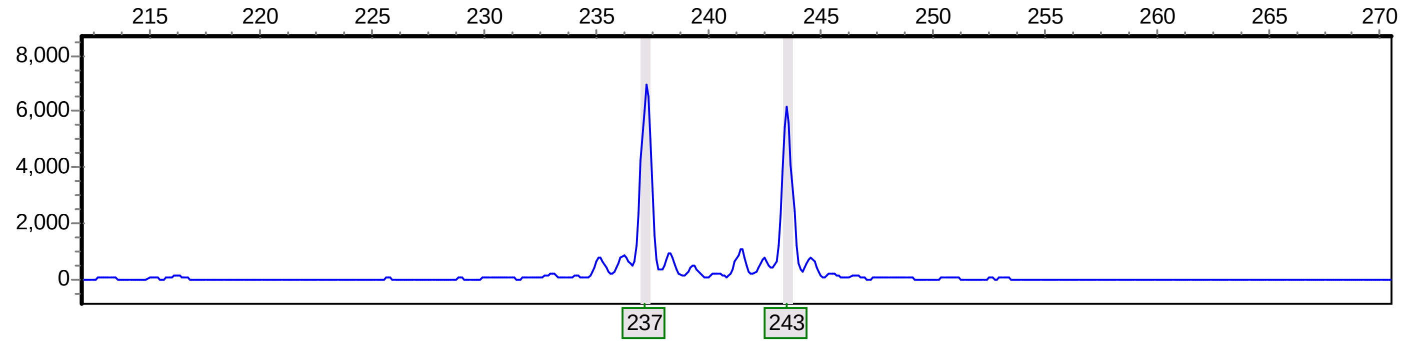

Sample 2 sorse2-04.B02.fsa Run date and time: 03/19/2017 - 18:17:56 -> 03/19/2017 - 19:01:55

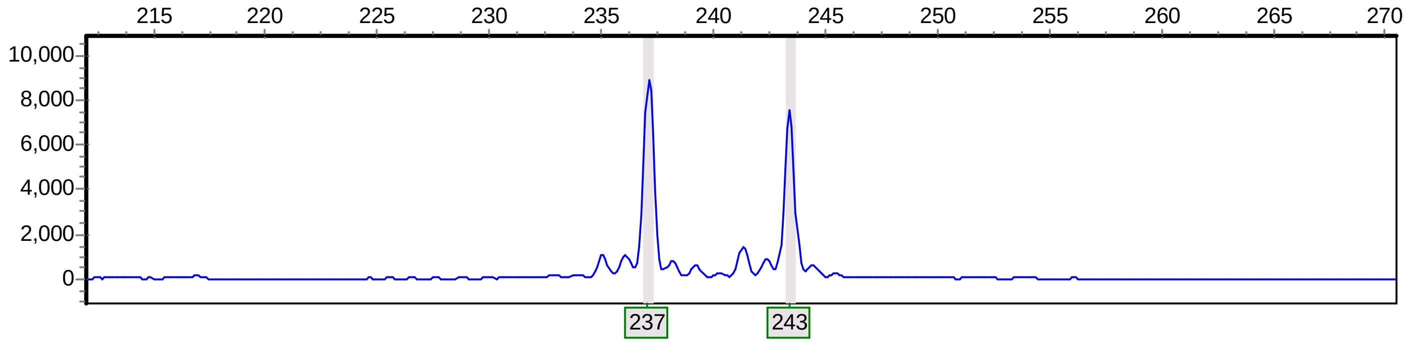

Sample 3 sorse2-05.A05.fsa Run date and time: 03/20/2017 - 21:37:15 -> 03/20/2017 - 22:21:17

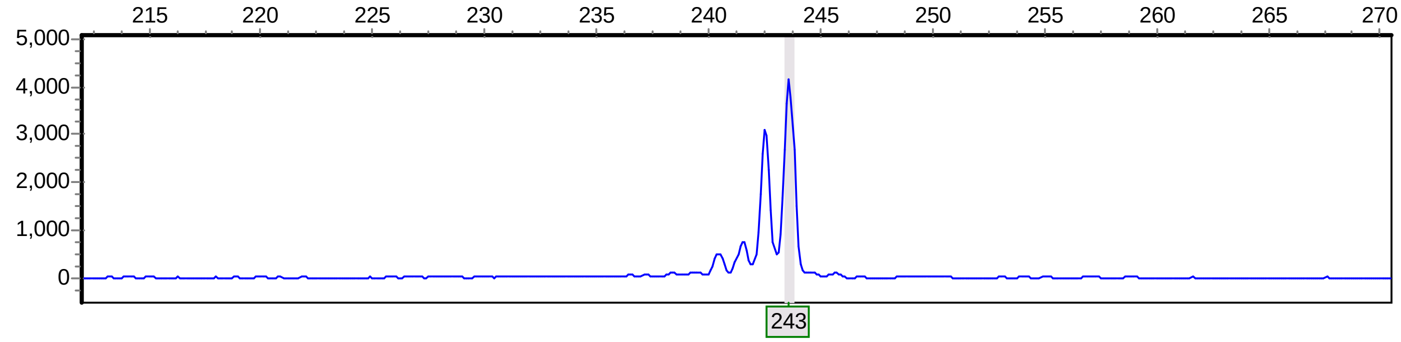

Sample 4 sorse2-06.B05.fsa Run date and time: 03/20/2017 - 21:37:15 -> 03/20/2017 - 22:21:17

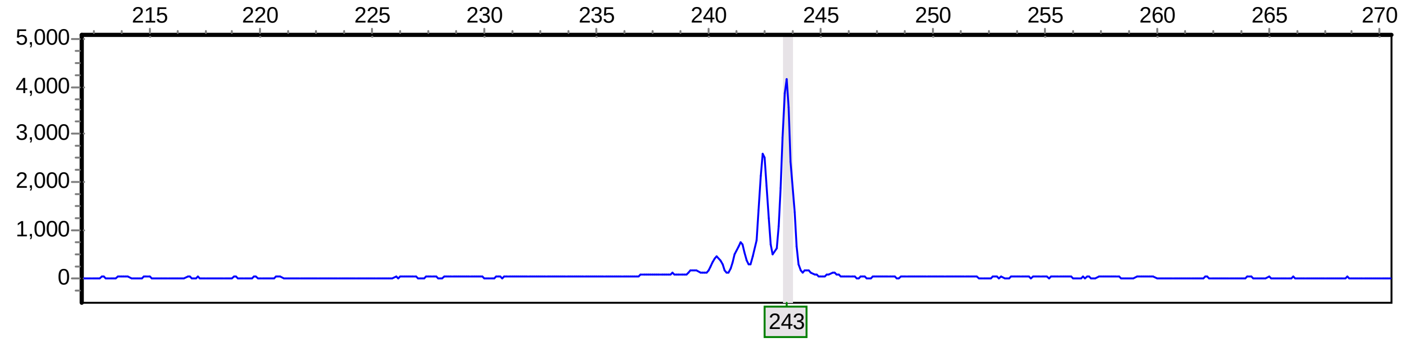

PDF 檔案使用 "pdfFactory Pro" 試用版本建立 www.pdffactory.com

Allele Report

Sample 5: sorse2-07.C05.fsa Run date and time: 03/20/2017 - 21:37:15 -> 03/20/2017 - 22:21:17

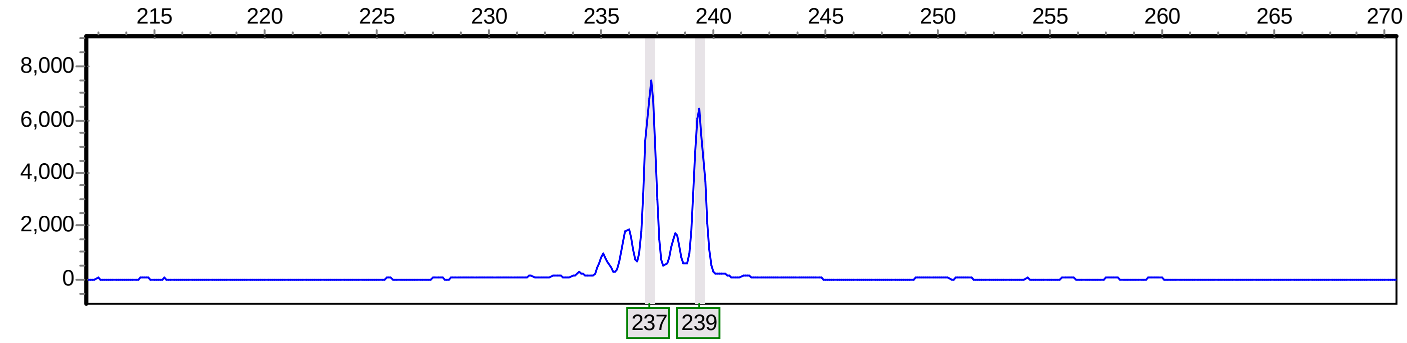

Sample 6 sorse2-08-1.E05.fsa Run date and time: 03/21/2017 - 13:32:38 -> 03/21/2017 - 14:23:07

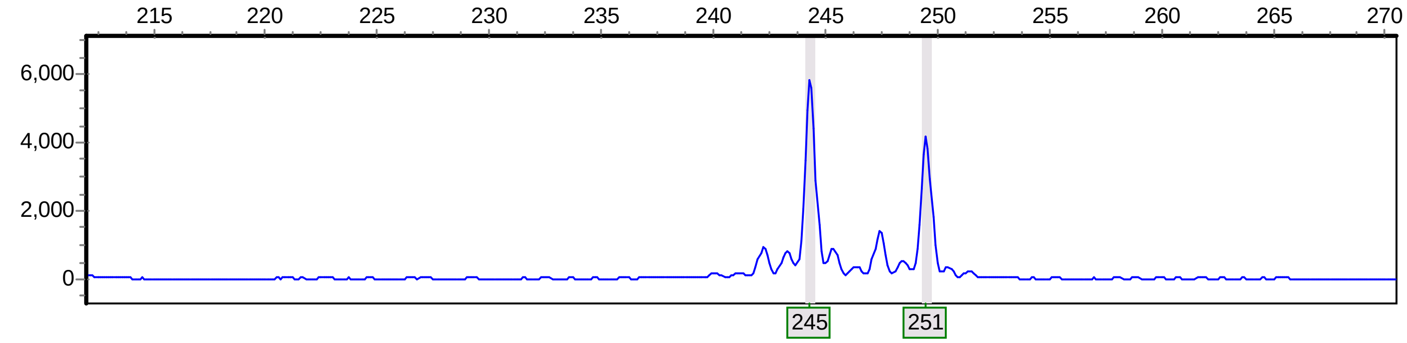

Sample 7 sorse2-08.D05.fsa Run date and time: 03/20/2017 - 21:37:15 -> 03/20/2017 - 22:21:17

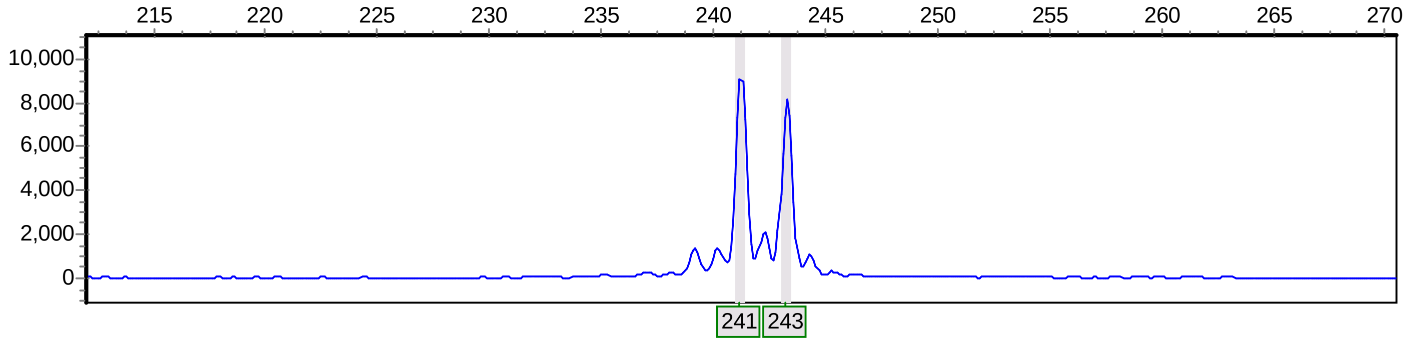

Sample 8 sorse2-09.F05.fsa Run date and time: 03/21/2017 - 13:32:38 -> 03/21/2017 - 14:23:07

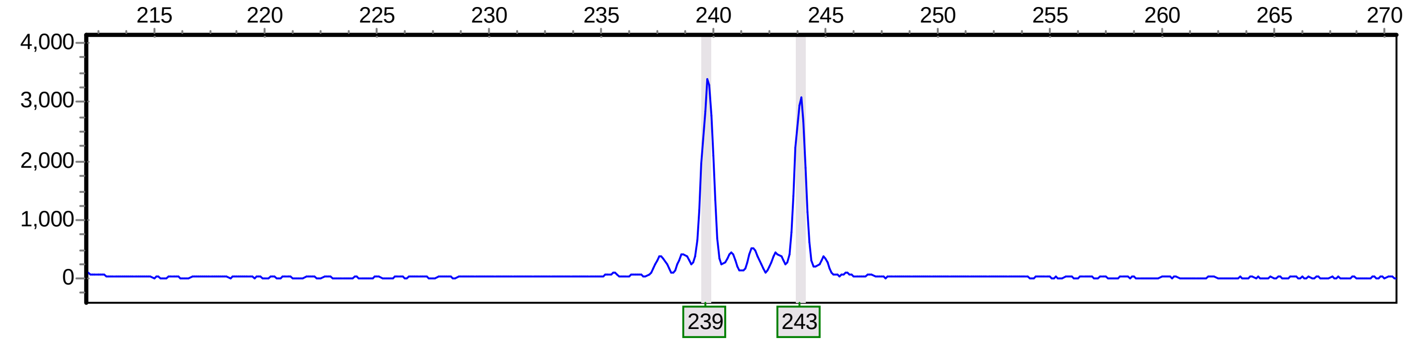

PDF 檔案使用 "pdfFactory Pro" 試用版本建立 www.pdffactory.com

Allele Report

Sample 1: sorse5-01.A07.fsa Run date and time: 03/20/2017 - 21:37:15 -> 03/20/2017 - 22:21:17

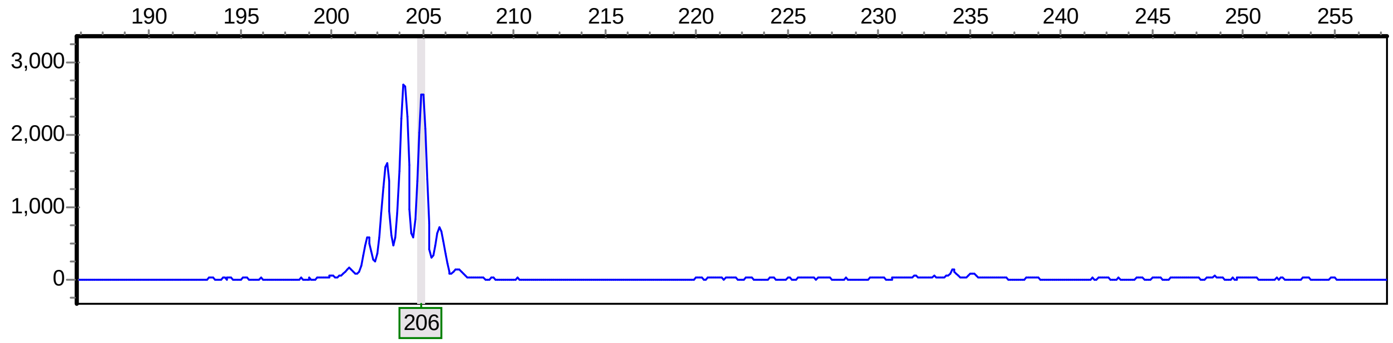

Sample 2 sorse5-04.B07.fsa Run date and time: 03/21/2017 - 13:32:38 -> 03/21/2017 - 14:23:07

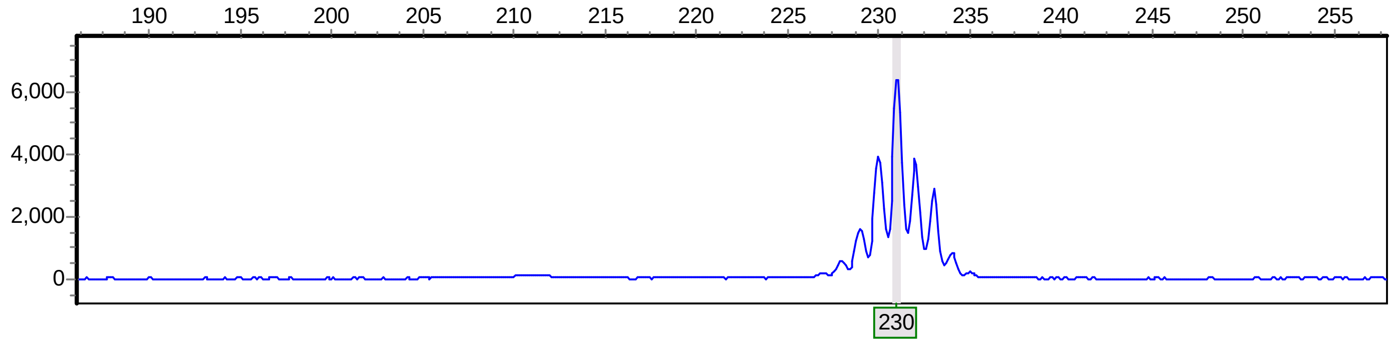

Sample 3 sorse5-05.C03.fsa Run date and time: 03/19/2017 - 18:17:56 -> 03/19/2017 - 19:01:55

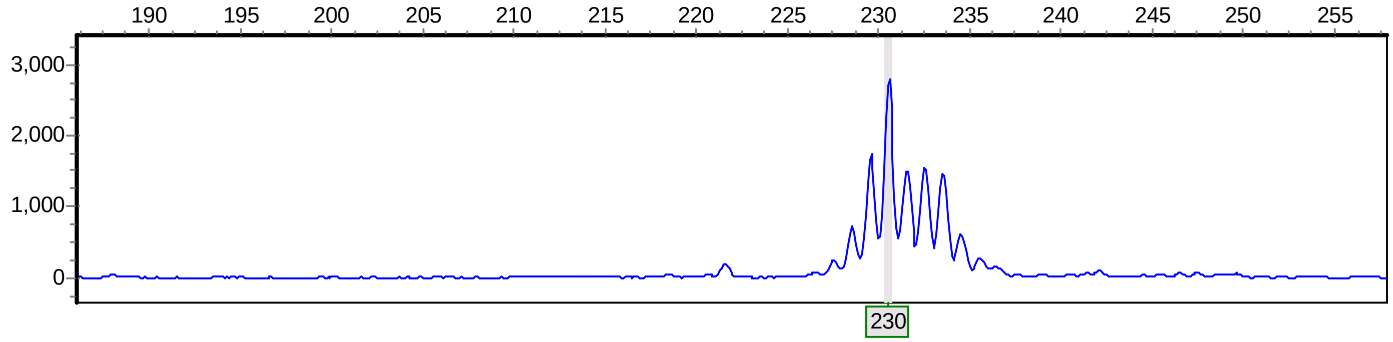

Sample 4 sorse5-06.D03.fsa Run date and time: 03/19/2017 - 18:17:56 -> 03/19/2017 - 19:01:55

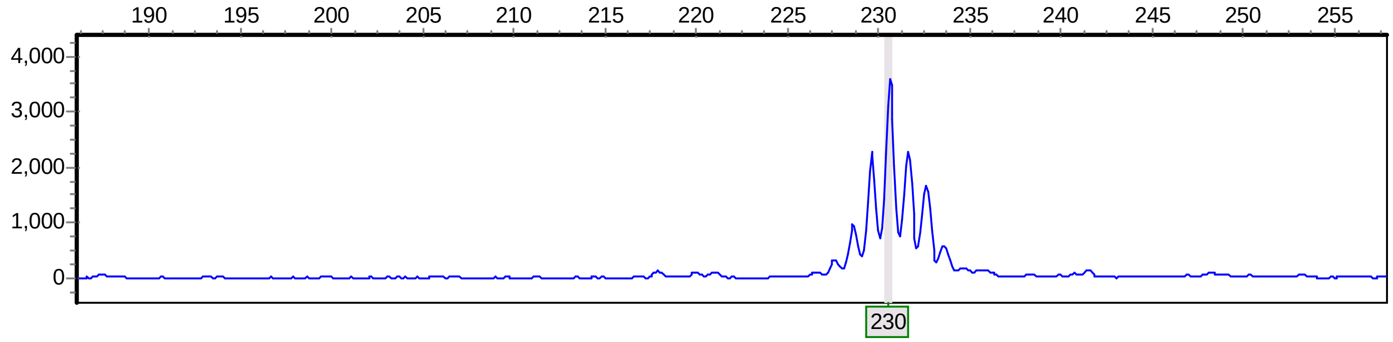

PDF 檔案使用 "pdfFactory Pro" 試用版本建立 www.pdffactory.com

Allele Report

Sample 5: sorse5-07.C07.fsa Run date and time: 03/20/2017 - 21:37:15 -> 03/20/2017 - 22:21:17

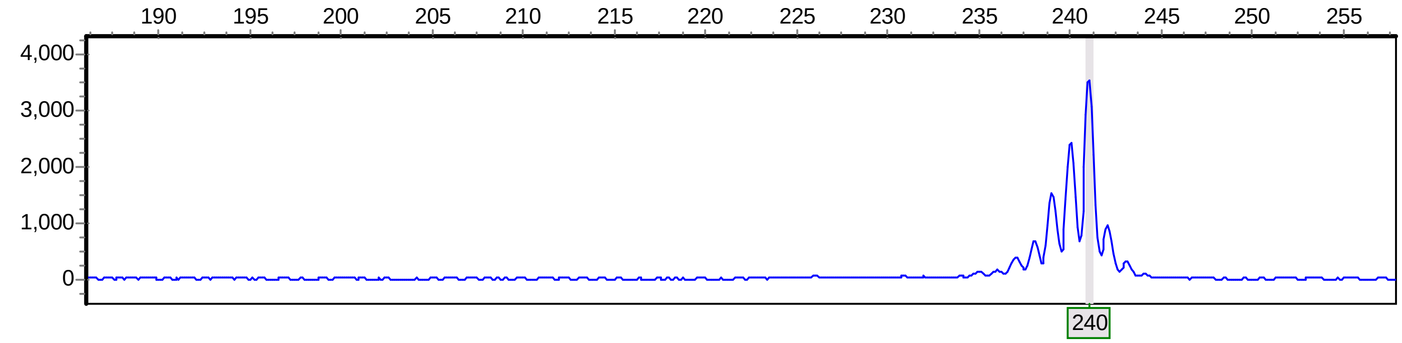

Sample 6 sorse5-08-1.E07.fsa Run date and time: 03/20/2017 - 21:37:15 -> 03/20/2017 - 22:21:17

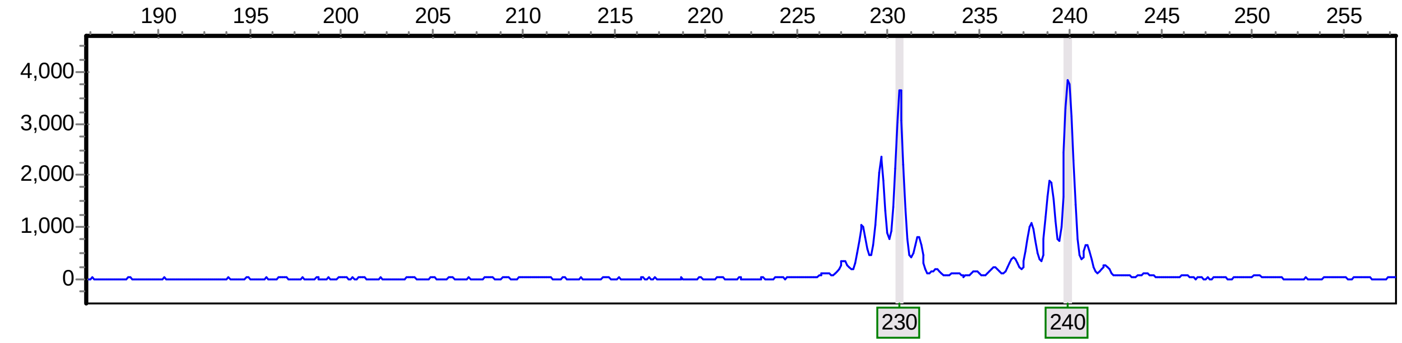

Sample 7 sorse5-08.D07.fsa Run date and time: 03/20/2017 - 21:37:15 -> 03/20/2017 - 22:21:17

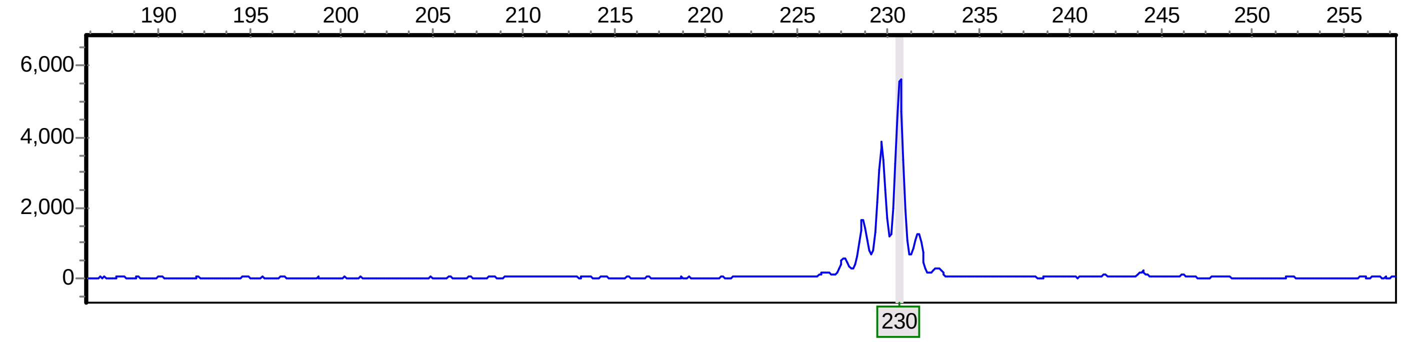

Sample 8 sorse5-09.F07.fsa Run date and time: 03/20/2017 - 21:37:15 -> 03/20/2017 - 22:21:17

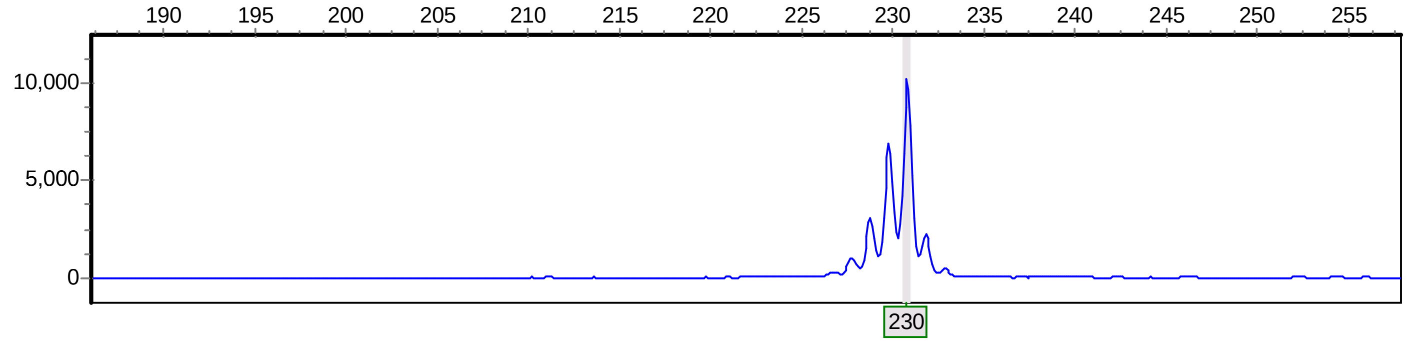

PDF 檔案使用 "pdfFactory Pro" 試用版本建立 www.pdffactory.com

Allele Report

Sample 1: sorse10-01.A08.fsa Run date and time: 03/20/2017 - 21:37:15 -> 03/20/2017 - 22:21:17

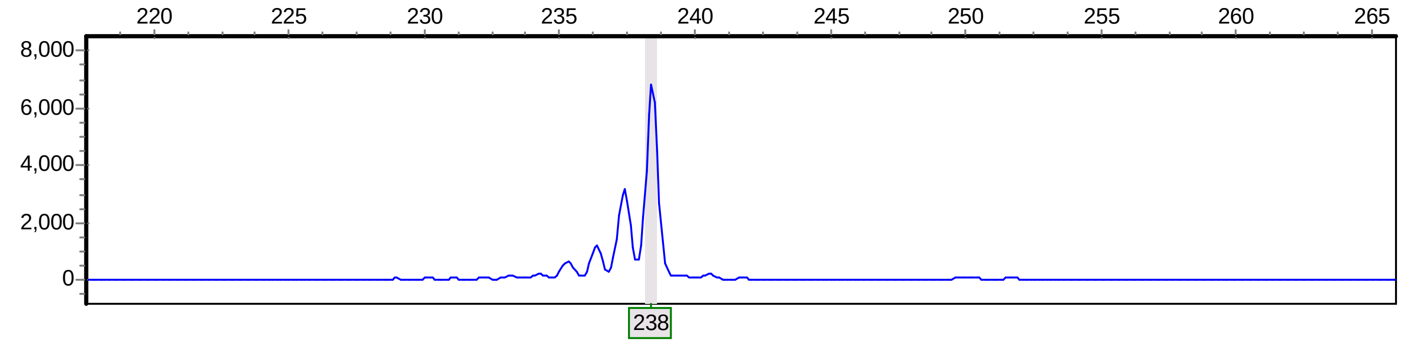

Sample 2 sorse10-04.B08.fsa Run date and time: 03/20/2017 - 21:37:15 -> 03/20/2017 - 22:21:17

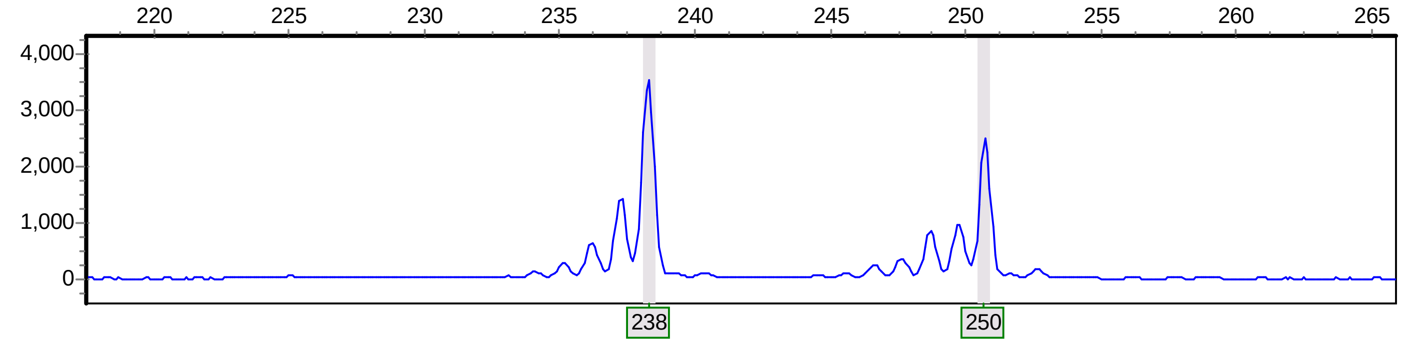

Sample 3 sorse10-05.E03.fsa Run date and time: 03/19/2017 - 18:17:56 -> 03/19/2017 - 19:01:55

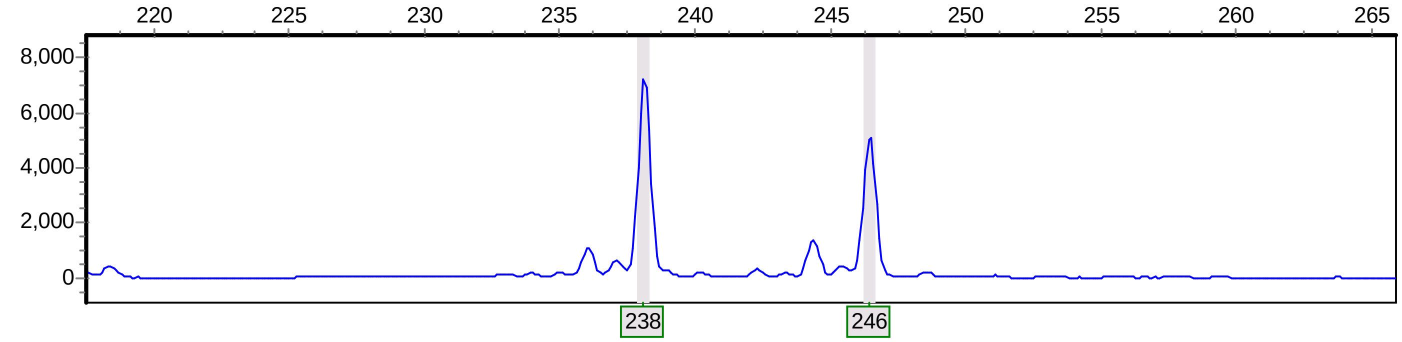

Sample 4 sorse10-06.F03.fsa Run date and time: 03/19/2017 - 18:17:56 -> 03/19/2017 - 19:01:55

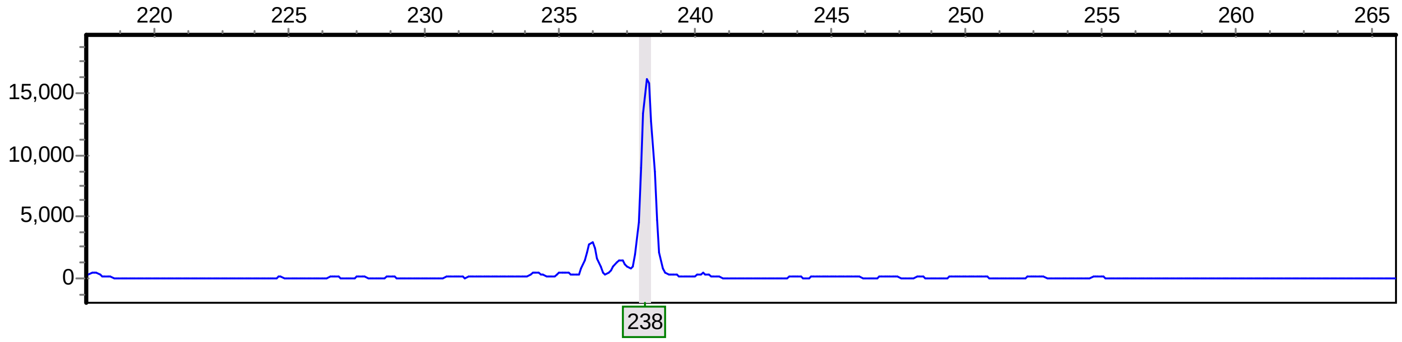

PDF 檔案使用 "pdfFactory Pro" 試用版本建立 www.pdffactory.com

Sample 5: sorse10-07.C08.fsa Run date and time: 03/20/2017 - 21:37:15 -> 03/20/2017 - 22:21:17

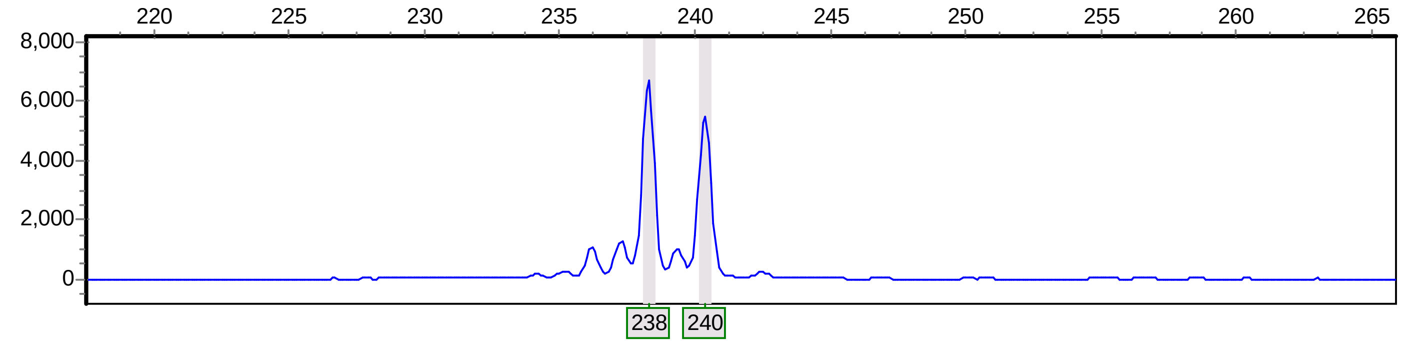

Sample 6 sorse10-08-1.E08.fsa Run date and time: 03/20/2017 - 21:37:15 -> 03/20/2017 - 22:21:17

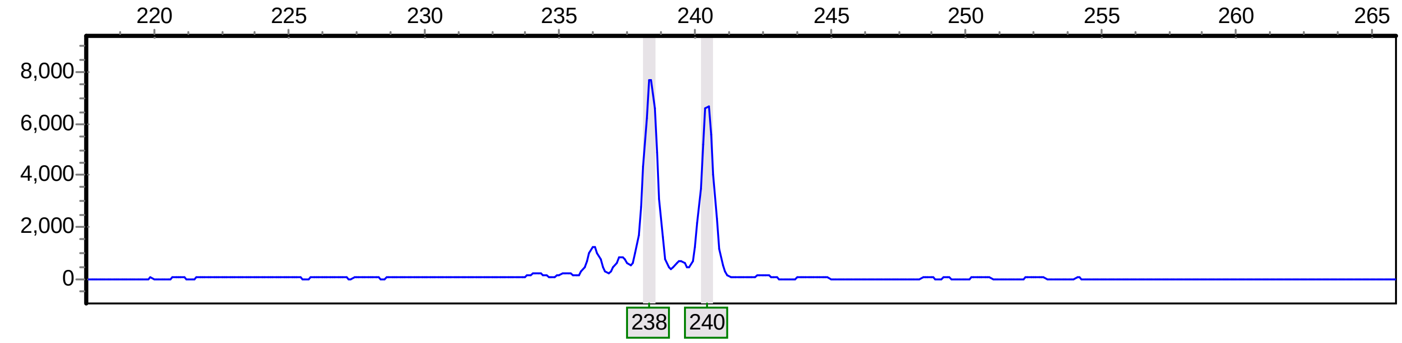

Sample 7 sorse10-08.D08.fsa Run date and time: 03/20/2017 - 21:37:15 -> 03/20/2017 - 22:21:17

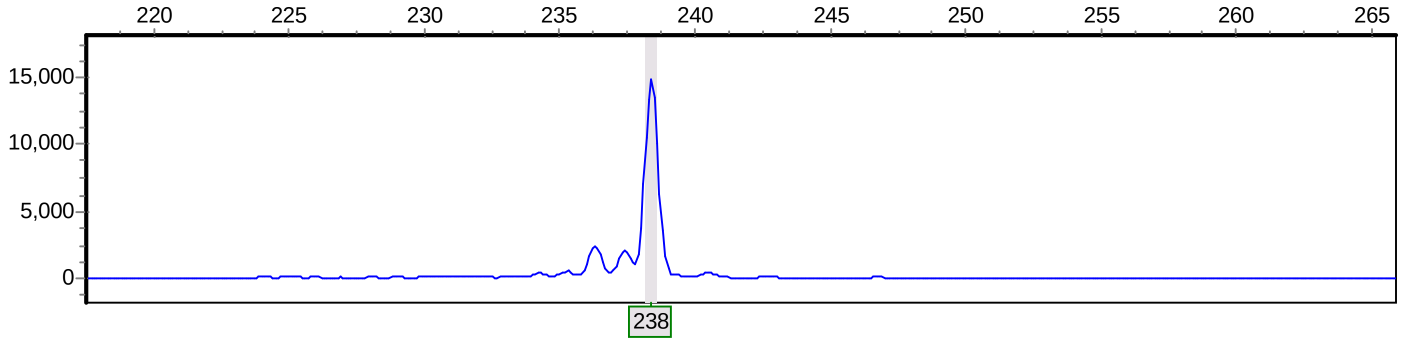

Sample 8 sorse10-09.F08.fsa Run date and time: 03/20/2017 - 21:37:15 -> 03/20/2017 - 22:21:17

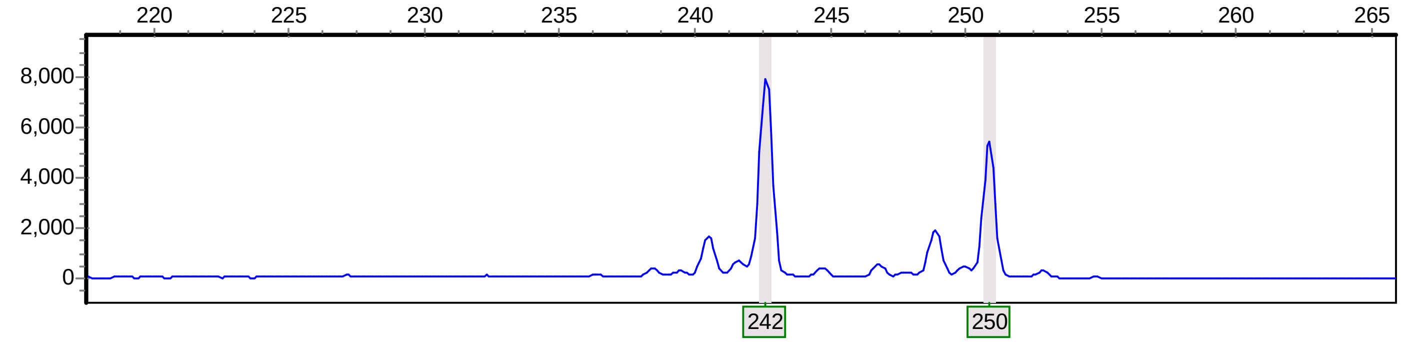

PDF 檔案使用 "pdfFactory Pro" 試用版本建立 www.pdffactory.com

Allele Report

Sample 1: sorse17-01.A09.fsa Run date and time: 03/20/2017 - 21:37:15 -> 03/20/2017 - 22:21:17

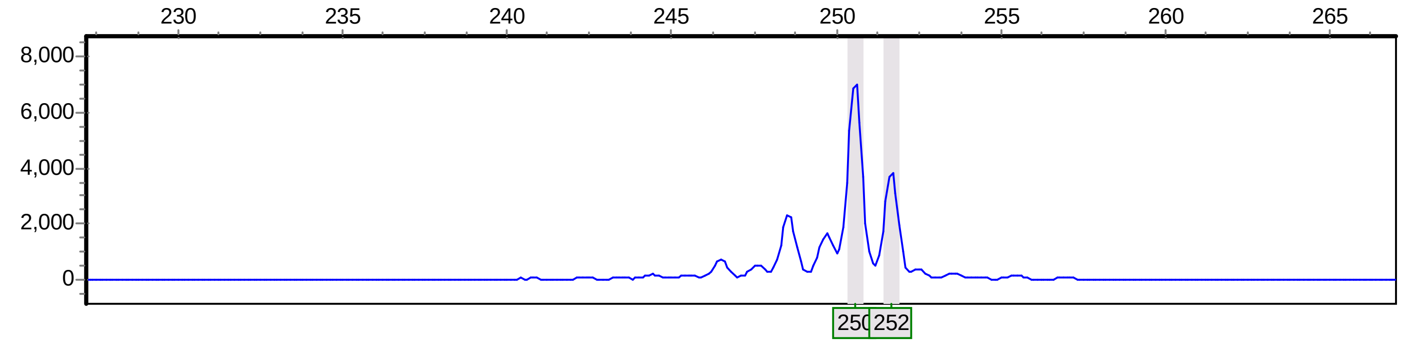

Sample 2 sorse17-04.B09.fsa Run date and time: 03/20/2017 - 21:37:15 -> 03/20/2017 - 22:21:17

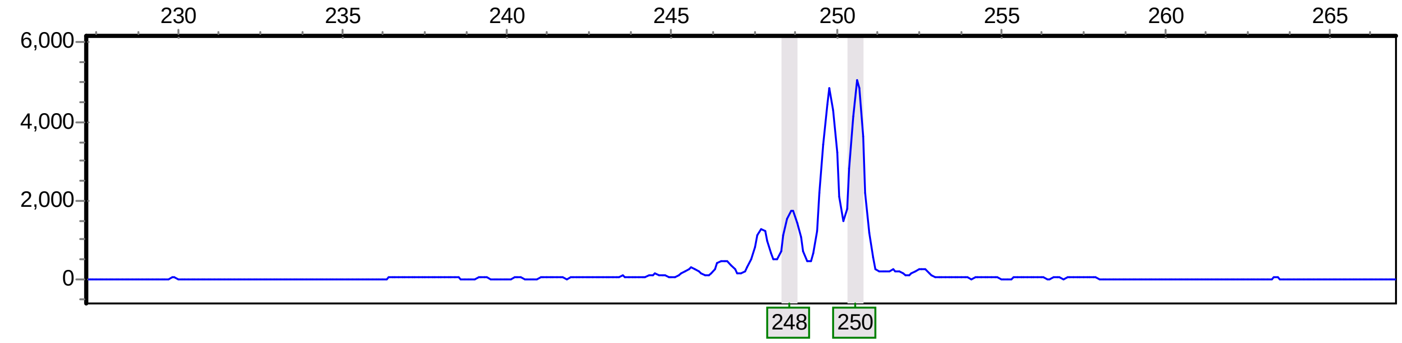

Sample 3 sorse17-05.G03.fsa Run date and time: 03/19/2017 - 18:17:56 -> 03/19/2017 - 19:01:55

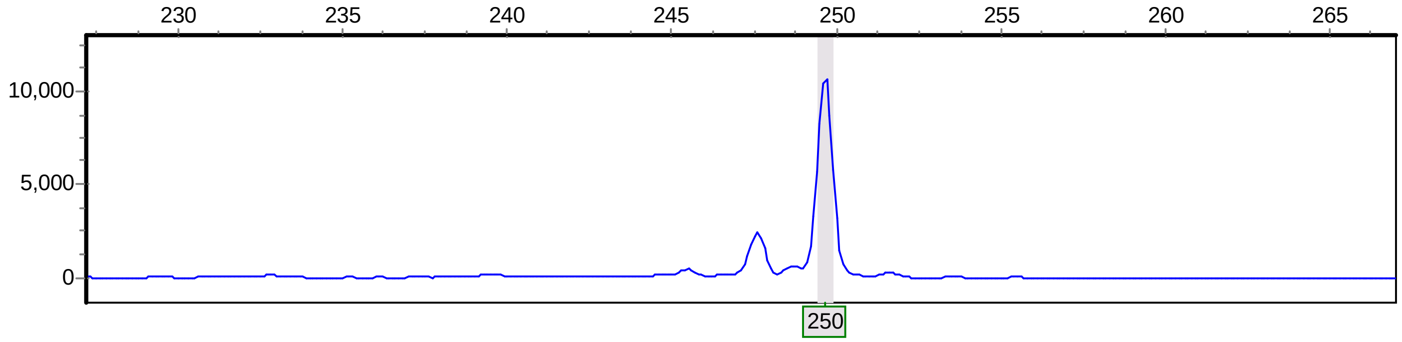

Sample 4 sorse17-06.H03.fsa Run date and time: 03/19/2017 - 18:17:56 -> 03/19/2017 - 19:01:55

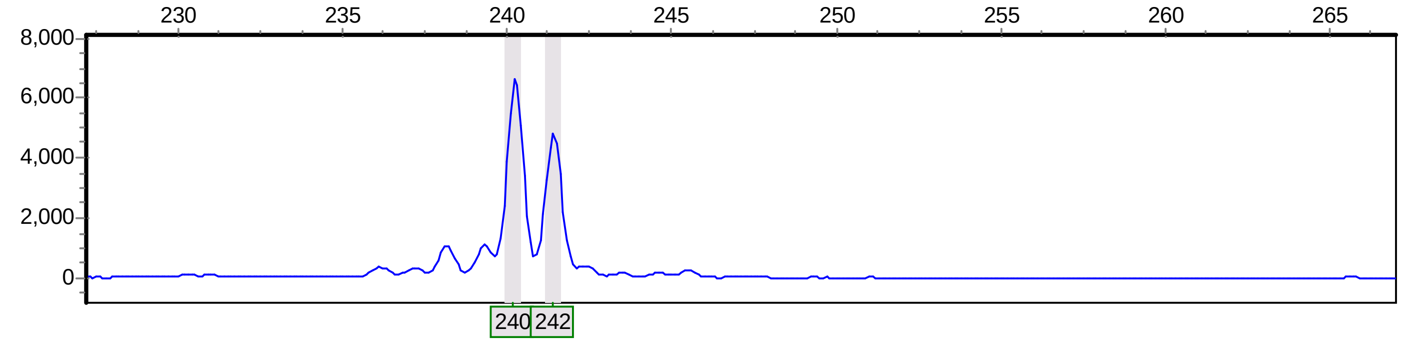

PDF 檔案使用 "pdfFactory Pro" 試用版本建立 www.pdffactory.com

Allele Report

Sample 5: sorse17-07.C09.fsa Run date and time: 03/20/2017 - 21:37:15 -> 03/20/2017 - 22:21:17

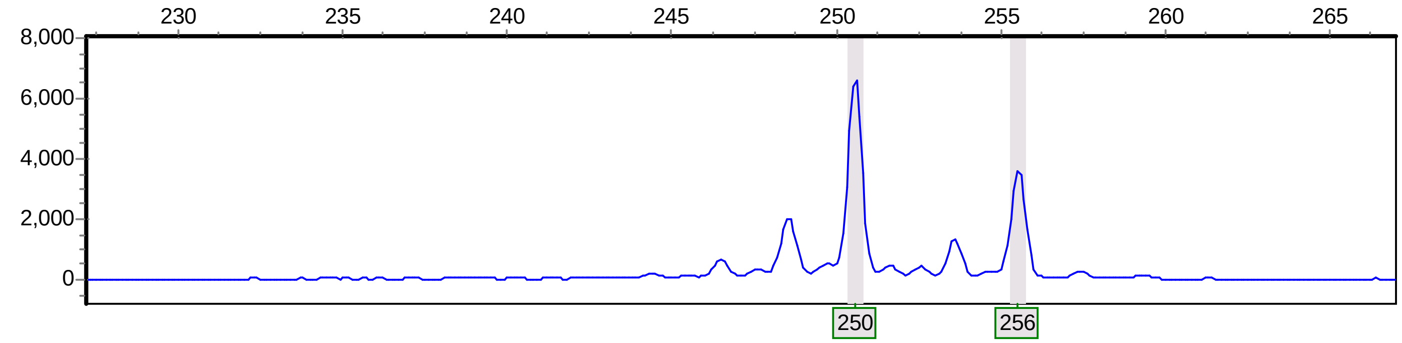

Sample 6 sorse17-08-1.E09.fsa Run date and time: 03/20/2017 - 21:37:15 -> 03/20/2017 - 22:21:17

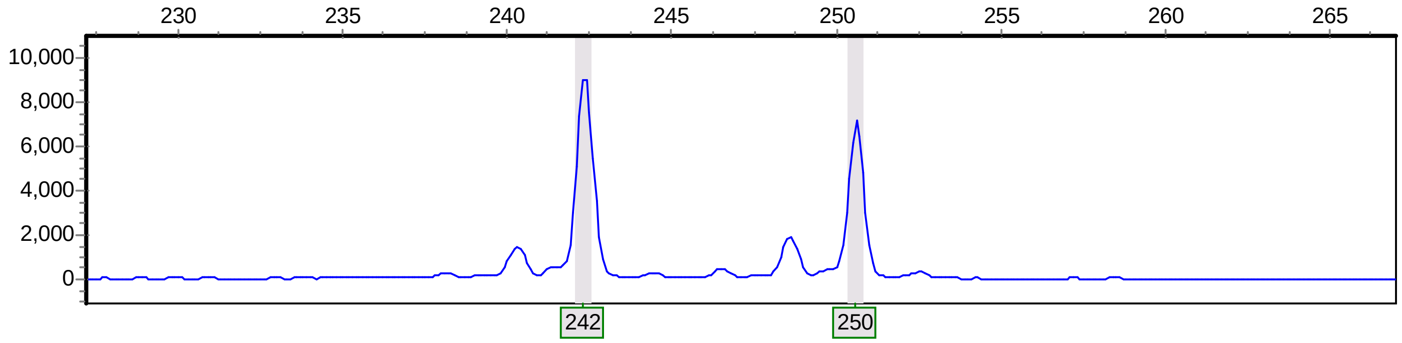

Sample 7 sorse17-08.D09.fsa Run date and time: 03/20/2017 - 21:37:15 -> 03/20/2017 - 22:21:17

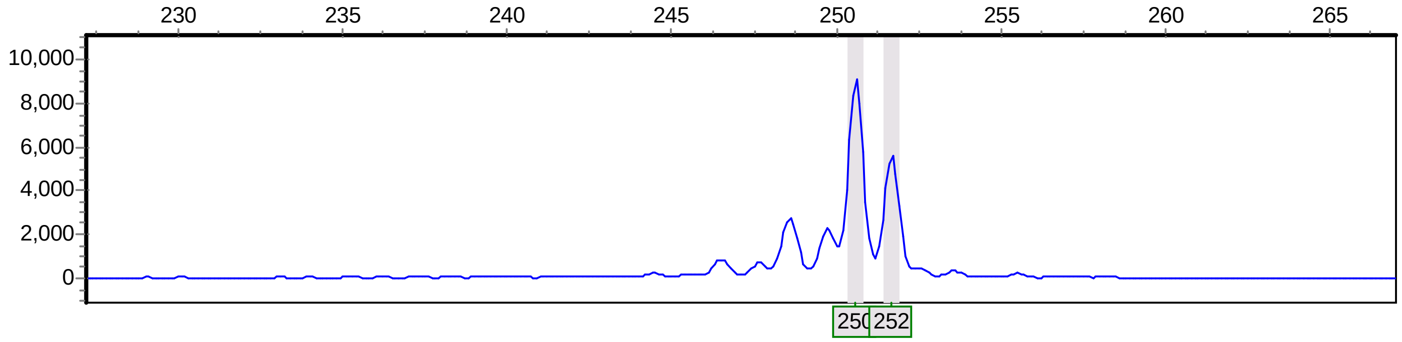

Sample 8 sorse17-09.F09.fsa Run date and time: 03/20/2017 - 21:37:15 -> 03/20/2017 - 22:21:17

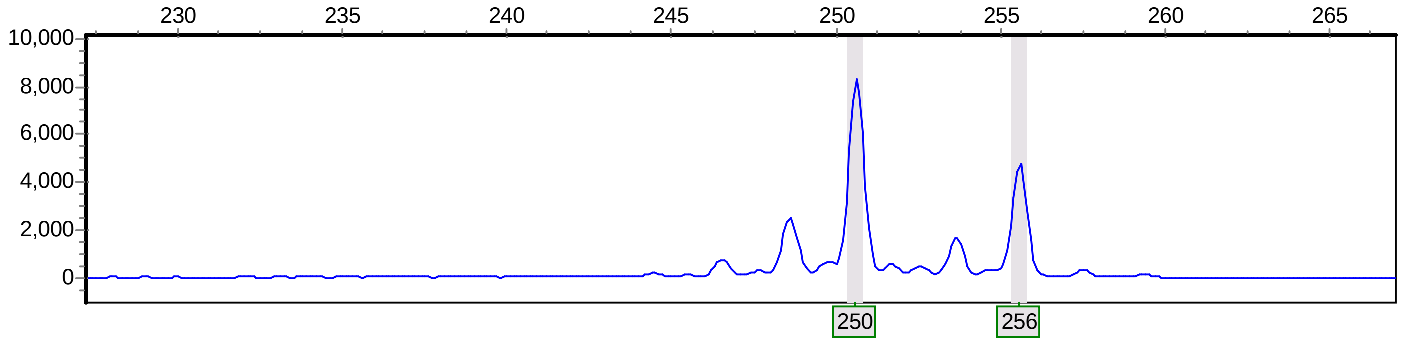

PDF 檔案使用 "pdfFactory Pro" 試用版本建立 www.pdffactory.com

SoftGenetics
GeneMarker V2.2.0

Allele Report

Sample 1: sorse19-01.A10.fsa Run date and time: 03/20/2017 - 21:37:15 -> 03/20/2017 - 22:21:17

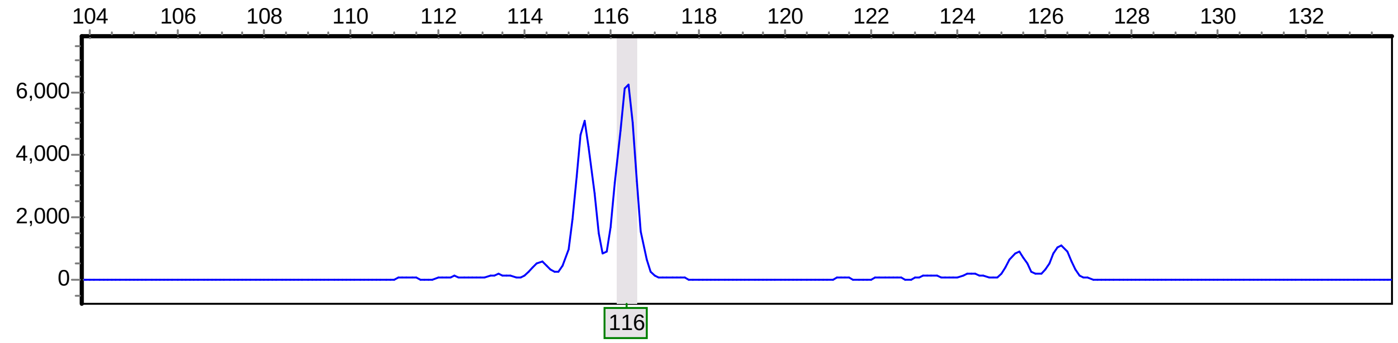

Sample 2 sorse19-04.B10.fsa Run date and time: 03/20/2017 - 21:37:15 -> 03/20/2017 - 22:21:17

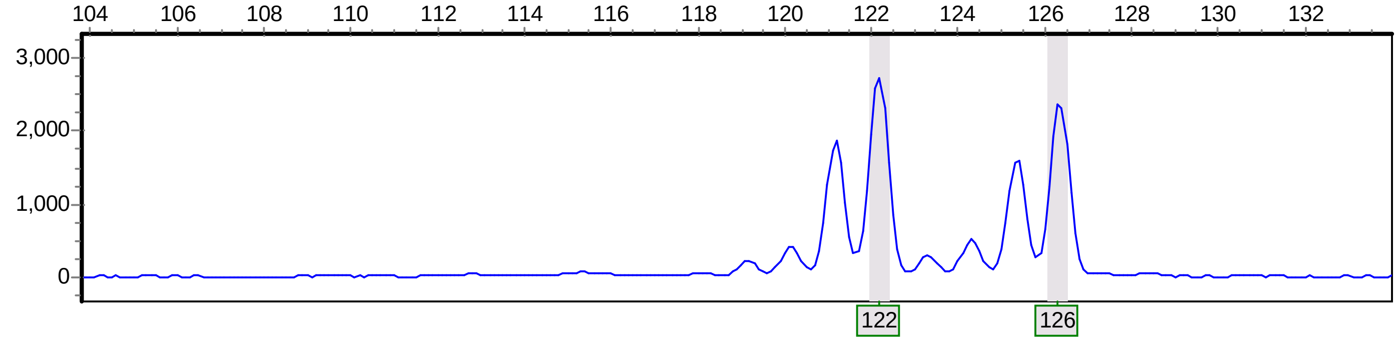

Sample 3 sorse19-05.C02.fsa Run date and time: 03/19/2017 - 18:17:56 -> 03/19/2017 - 19:01:55

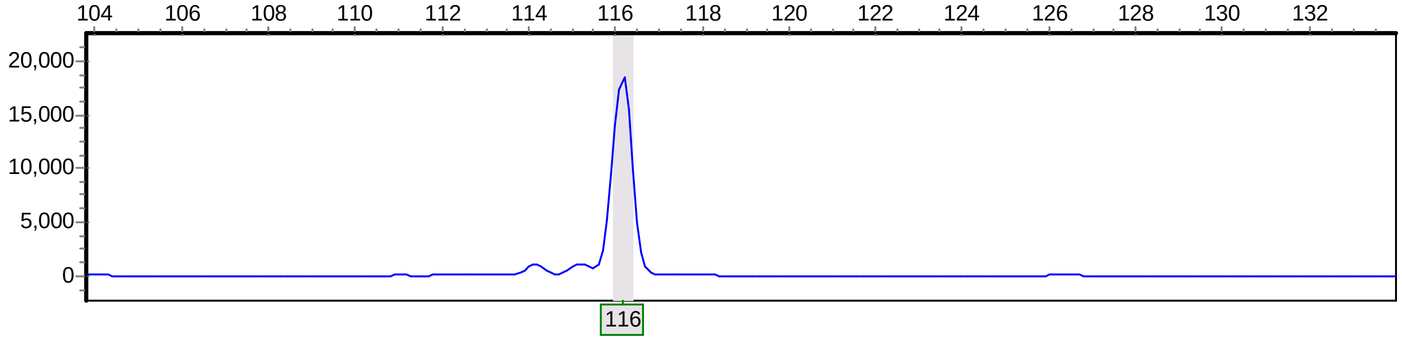

Sample 4 sorse19-06.D02.fsa Run date and time: 03/19/2017 - 18:17:56 -> 03/19/2017 - 19:01:55

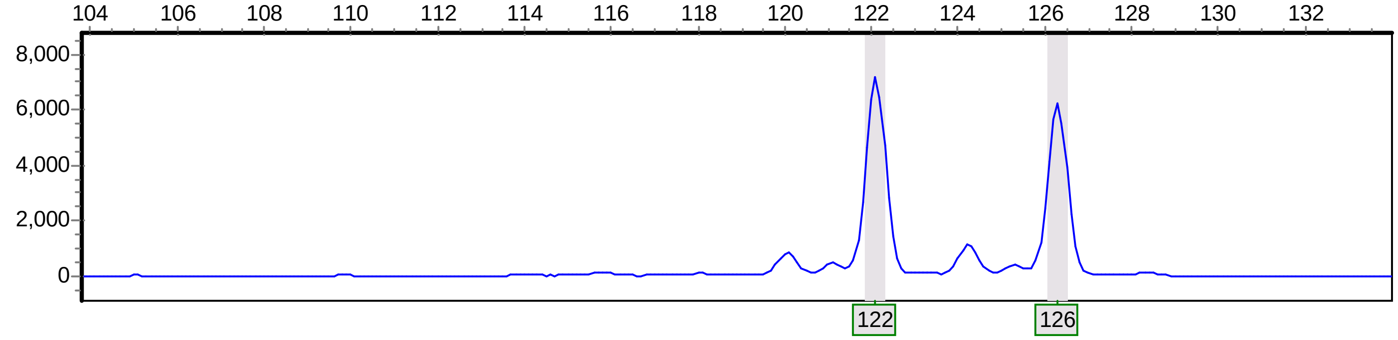

PDF 檔案使用 "pdfFactory Pro" 試用版本建立 www.pdffactory.com

Sample 5: sorse19-07.C10.fsa Run date and time: 03/20/2017 - 21:37:15 -> 03/20/2017 - 22:21:17

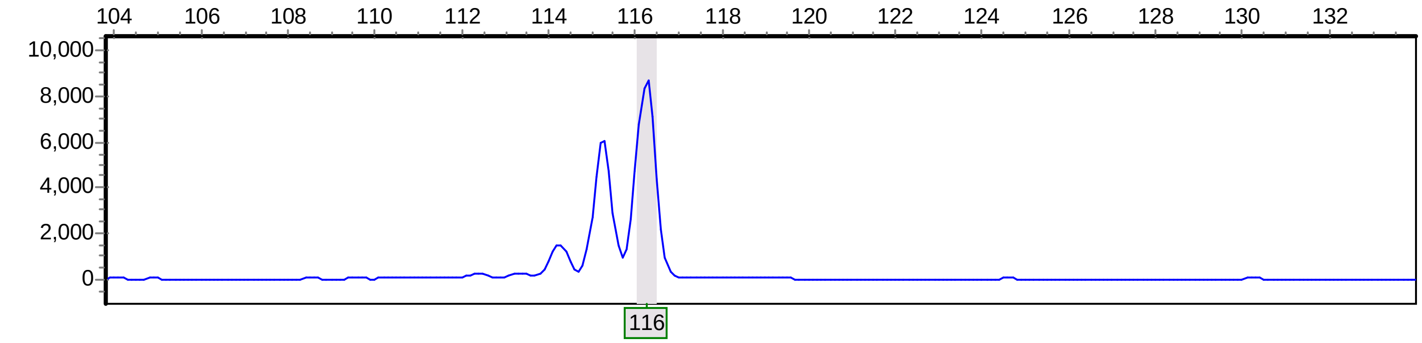

Sample 6 sorse19-08-1.E10.fsa Run date and time: 03/20/2017 - 21:37:15 -> 03/20/2017 - 22:21:17

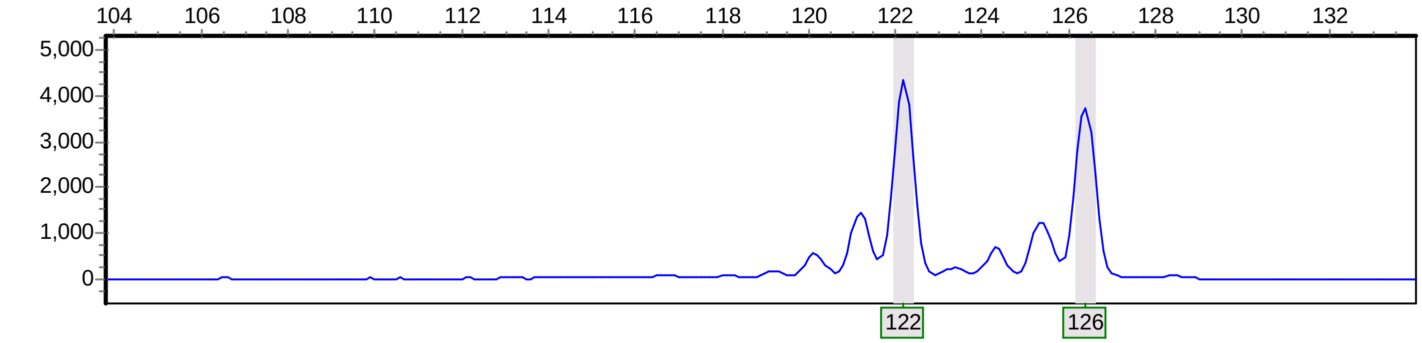

Sample 7 sorse19-08.D10.fsa Run date and time: 03/20/2017 - 21:37:15 -> 03/20/2017 - 22:21:17

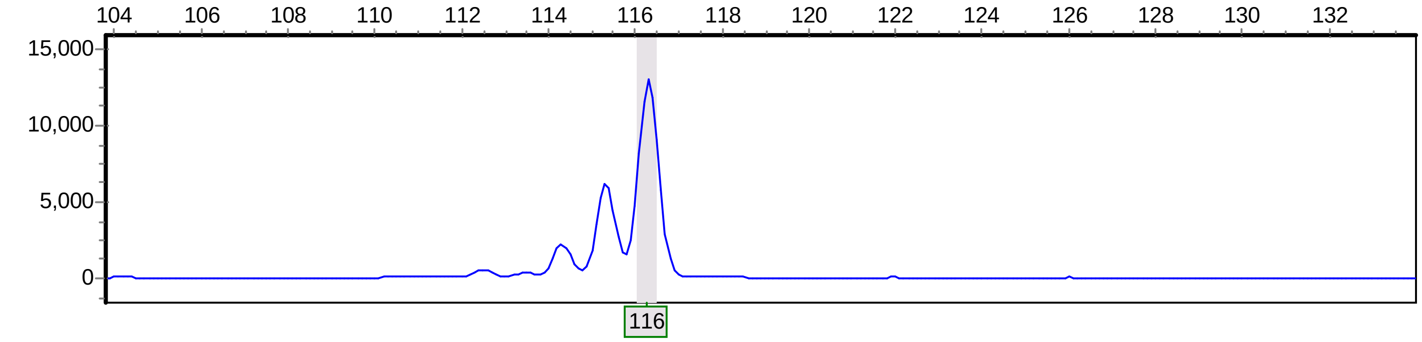

Sample 8 sorse19-09.F10.fsa Run date and time: 03/20/2017 - 21:37:15 -> 03/20/2017 - 22:21:17

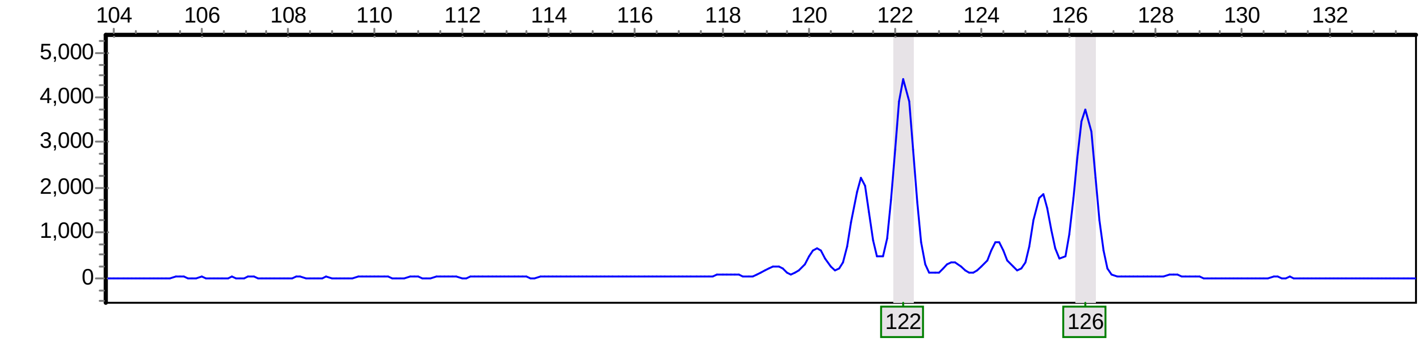

PDF 檔案使用 "pdfFactory Pro" 試用版本建立 www.pdffactory.com

Allele Report

Sample 1: sorse50-01.A12.fsa Run date and time: 03/21/2017 - 13:32:38 -> 03/21/2017 - 14:23:07

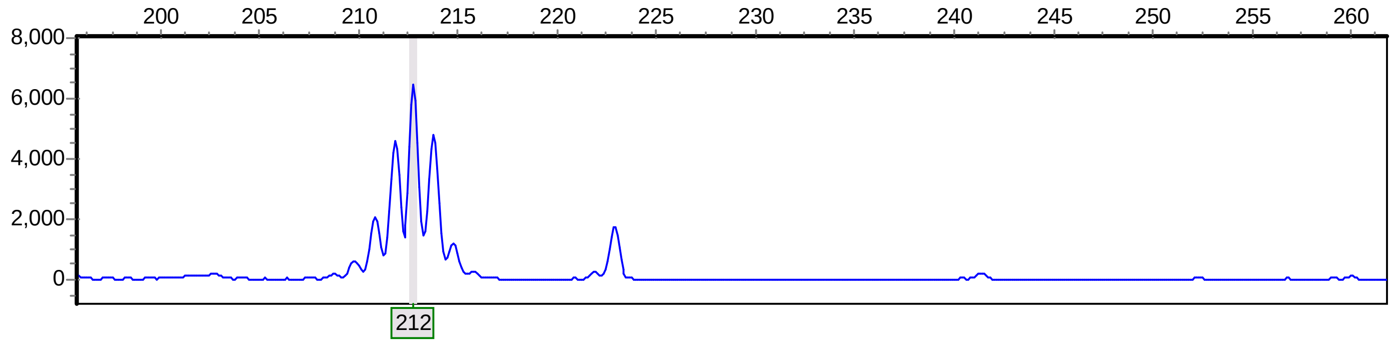

Sample 2 sorse50-04.B12.fsa Run date and time: 03/20/2017 - 21:37:15 -> 03/20/2017 - 22:21:17

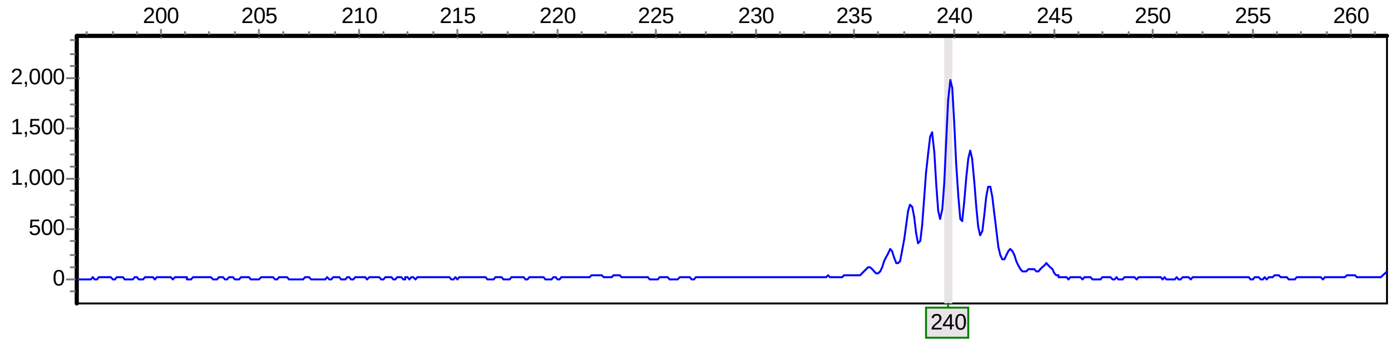

Sample 3 sorse50-05.C12.fsa Run date and time: 03/20/2017 - 21:37:15 -> 03/20/2017 - 22:21:17

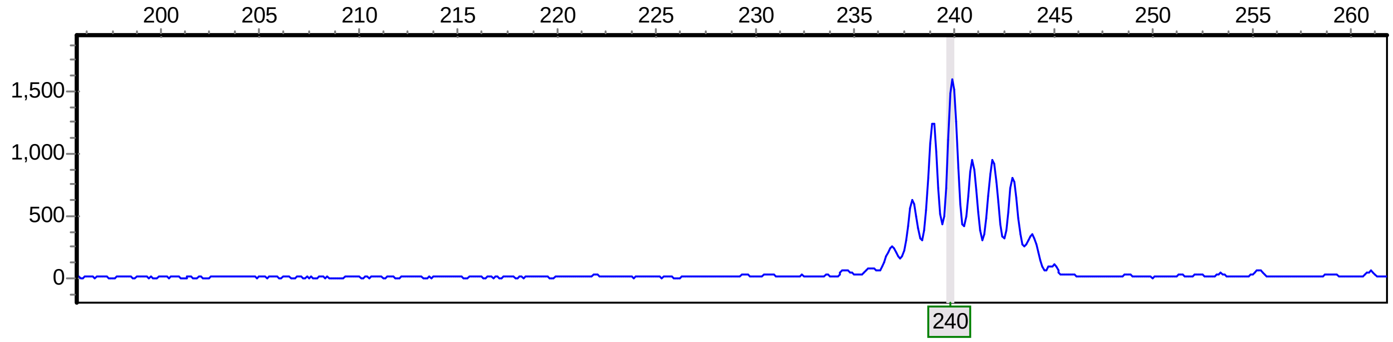

Sample 4 sorse50-06.D12.fsa Run date and time: 03/20/2017 - 21:37:15 -> 03/20/2017 - 22:21:17

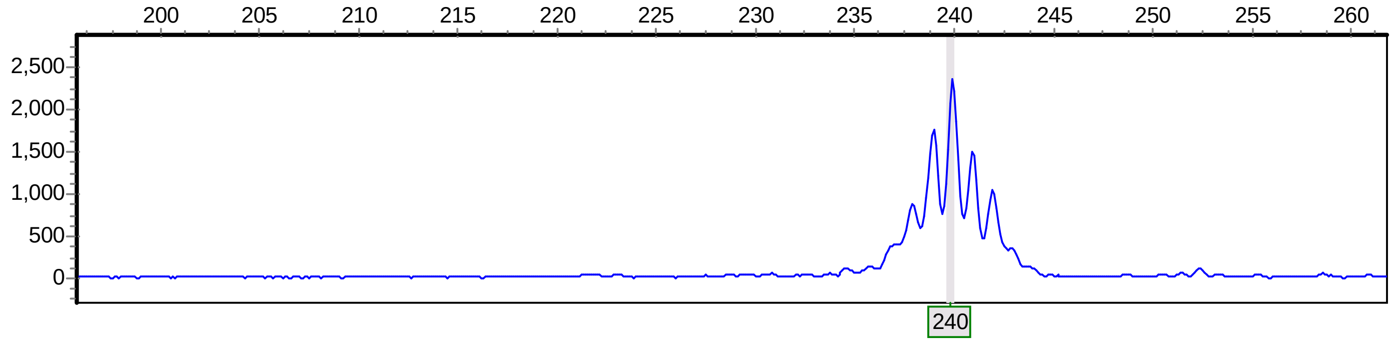

PDF 檔案使用 "pdfFactory Pro" 試用版本建立 www.pdffactory.com

Sample 5: sorse50-07.C04.fsa Run date and time: 03/19/2017 - 18:17:56 -> 03/19/2017 - 19:01:55

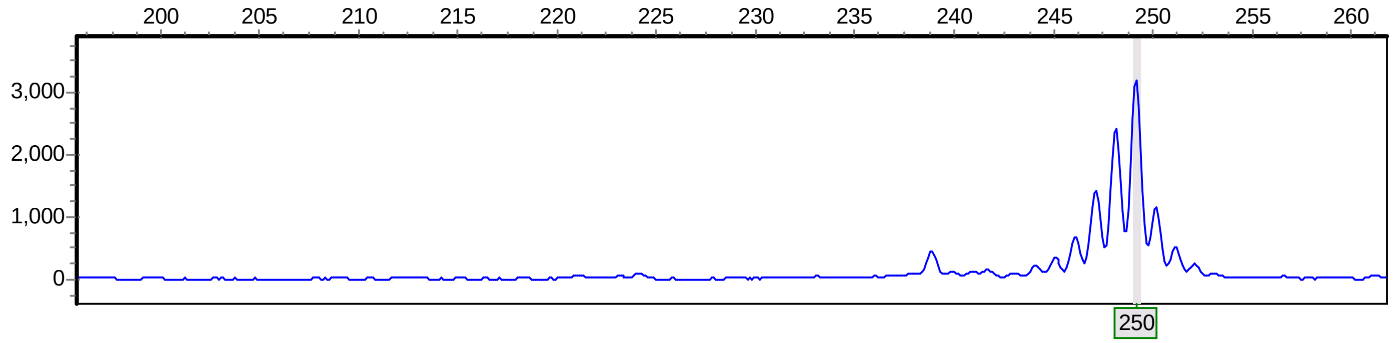

Sample 6 sorse50-08-1.E12.fsa Run date and time: 03/20/2017 - 21:37:15 -> 03/20/2017 - 22:21:17

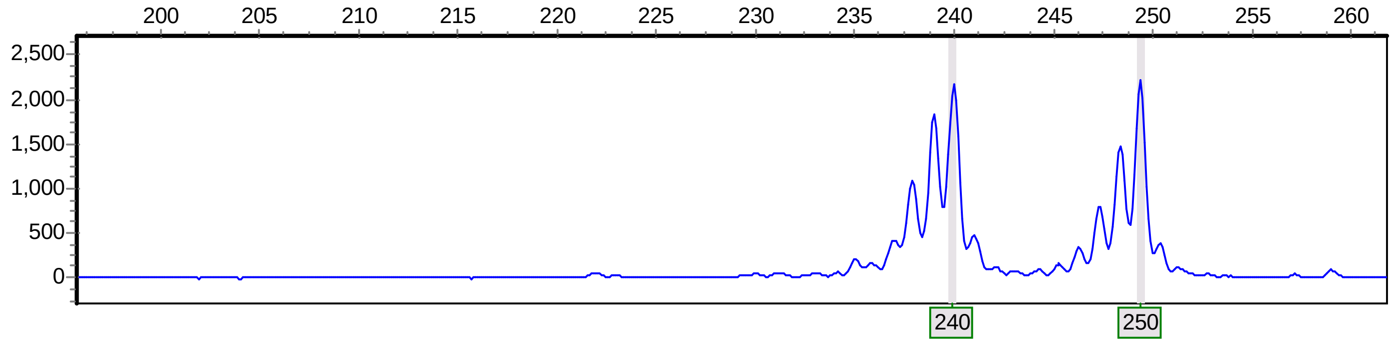

Sample 7 sorse50-08.D04.fsa Run date and time: 03/19/2017 - 18:17:56 -> 03/19/2017 - 19:01:55

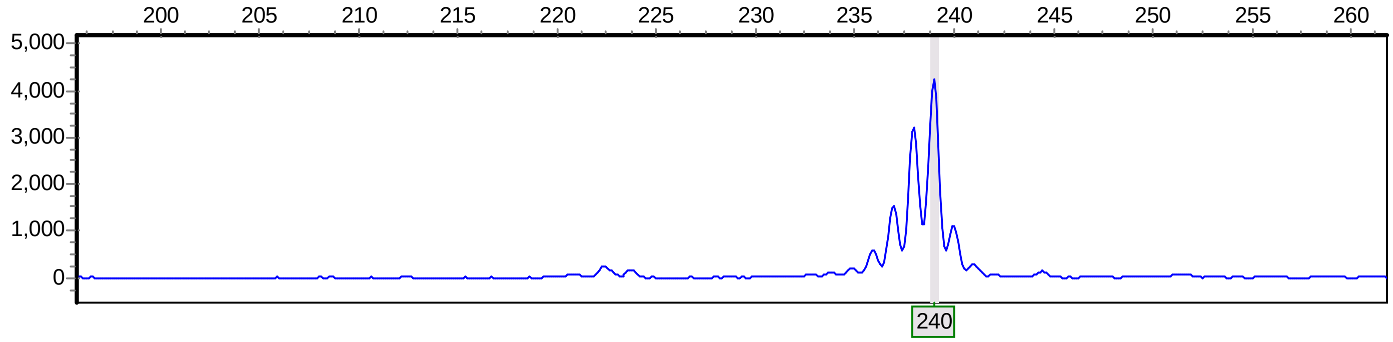

Sample 8 sorse50-09.F12.fsa Run date and time: 03/20/2017 - 21:37:15 -> 03/20/2017 - 22:21:17

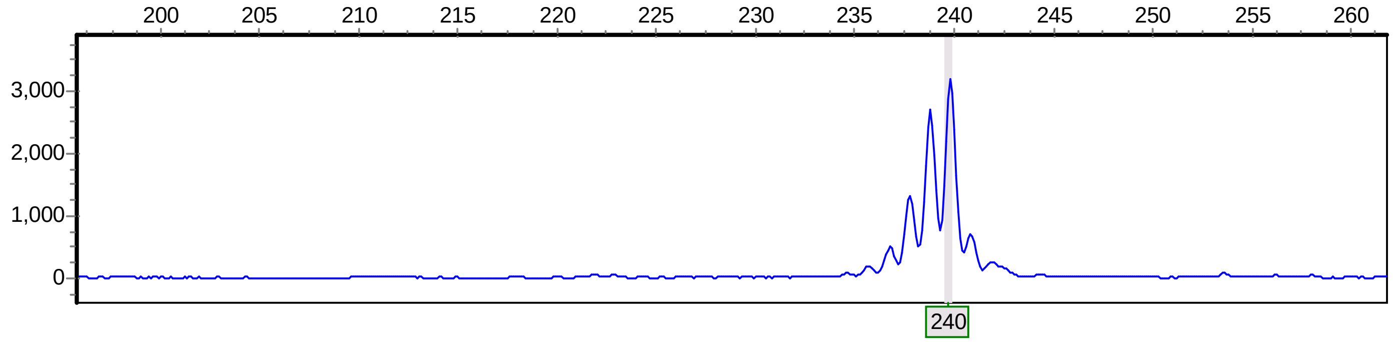

PDF 檔案使用 "pdfFactory Pro" 試用版本建立 www.pdffactory.com

Allele Report

Sample 1: sorsg1-01.A01.fsa Run date and time: 03/20/2017 - 22:22:03 -> 03/20/2017 - 23:06:41

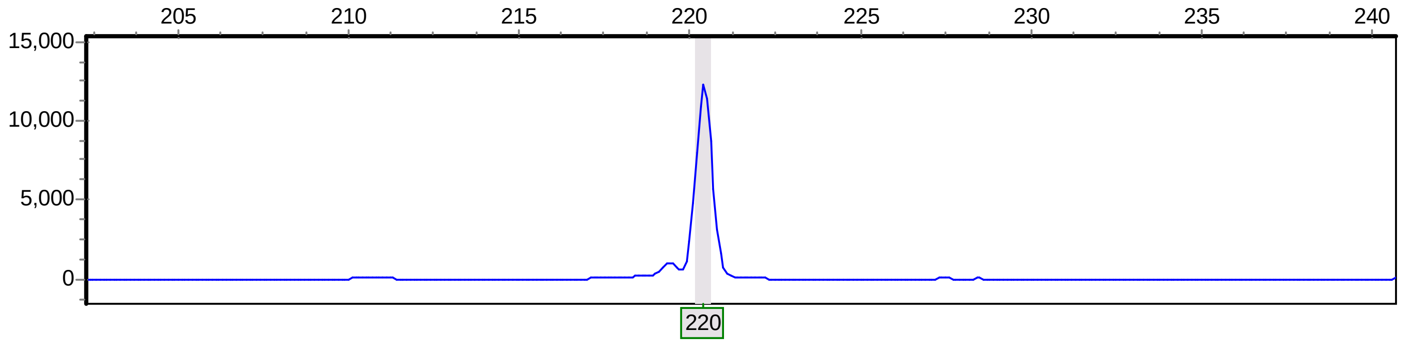

Sample 2 sorsg1-04.B01.fsa Run date and time: 03/20/2017 - 22:22:03 -> 03/20/2017 - 23:06:41

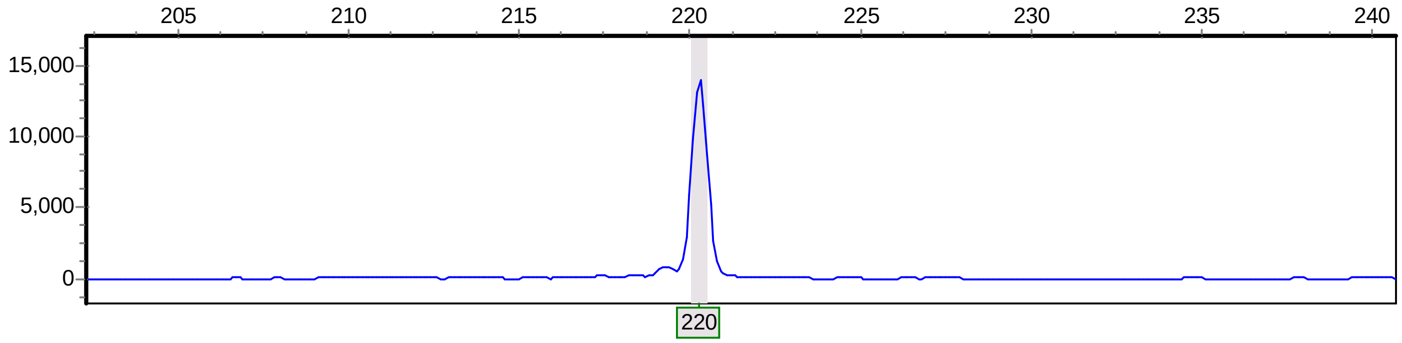

Sample 3 sorsg1-05.C01.fsa Run date and time: 03/20/2017 - 22:22:03 -> 03/20/2017 - 23:06:41

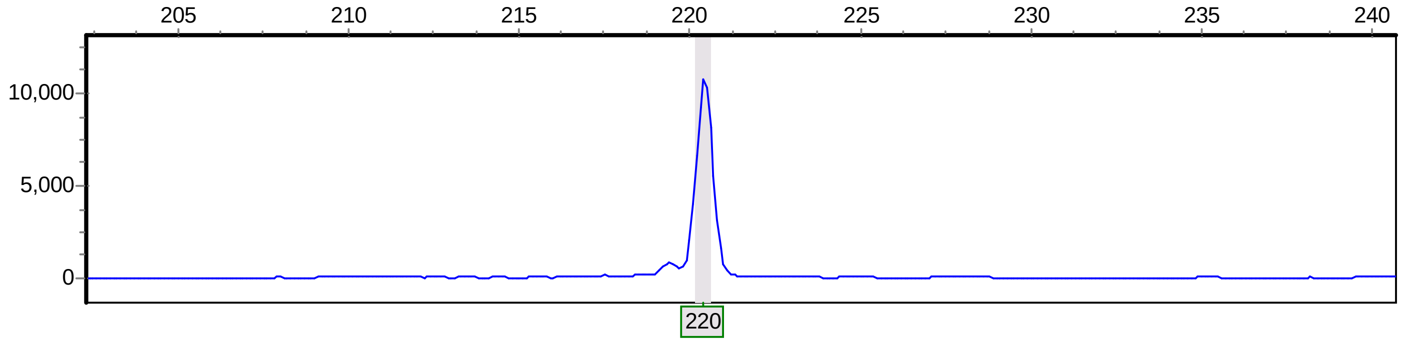

Sample 4 sorsg1-06.D01.fsa Run date and time: 03/20/2017 - 22:22:03 -> 03/20/2017 - 23:06:41

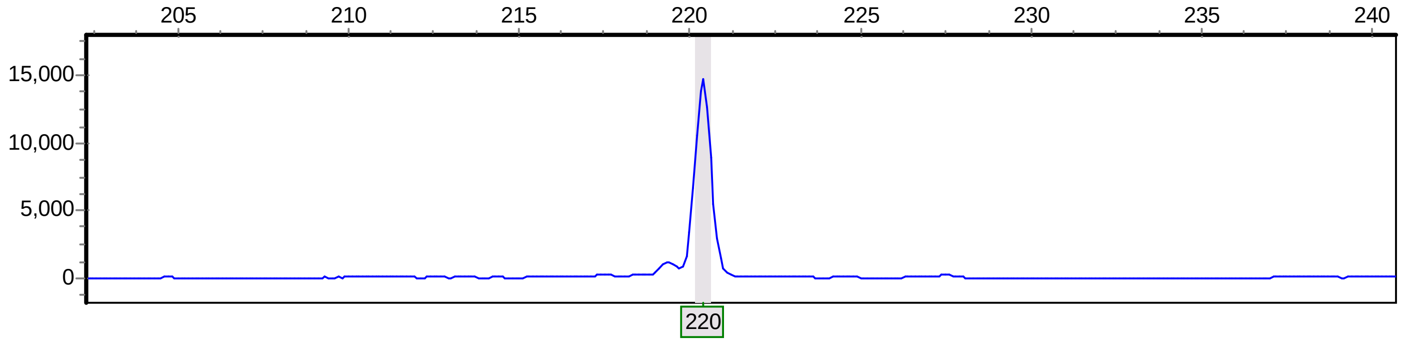

PDF 檔案使用 "pdfFactory Pro" 試用版本建立 www.pdffactory.com

Sample 5: sorsg1-07.E04.fsa Run date and time: 03/19/2017 - 18:17:56 -> 03/19/2017 - 19:01:55

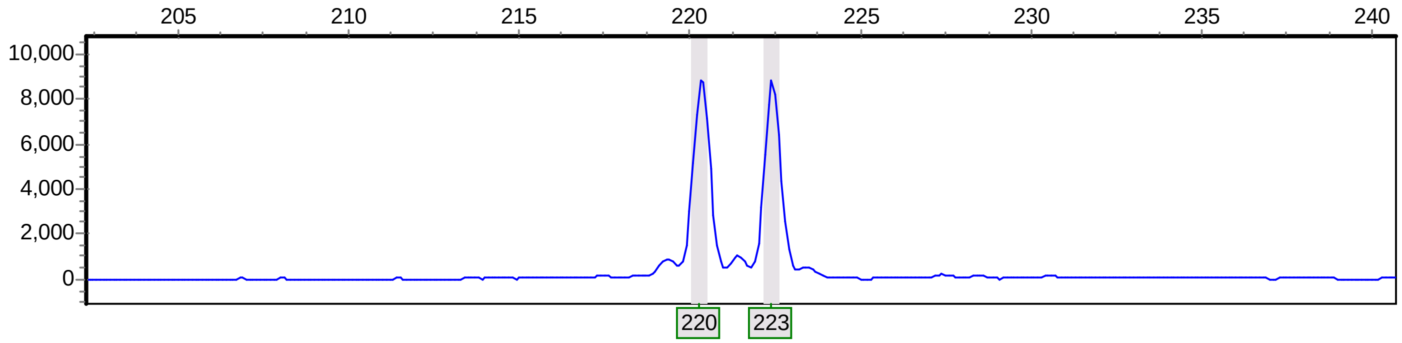

Sample 6 sorsg1-08-1.E01.fsa Run date and time: 03/20/2017 - 22:22:03 -> 03/20/2017 - 23:06:41

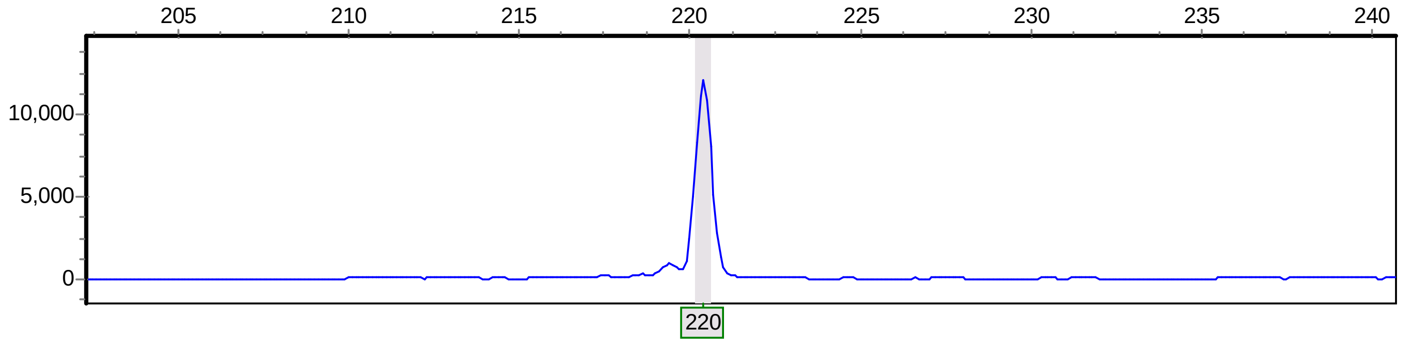

Sample 7 sorsg1-08.F04.fsa Run date and time: 03/19/2017 - 18:17:56 -> 03/19/2017 - 19:01:55

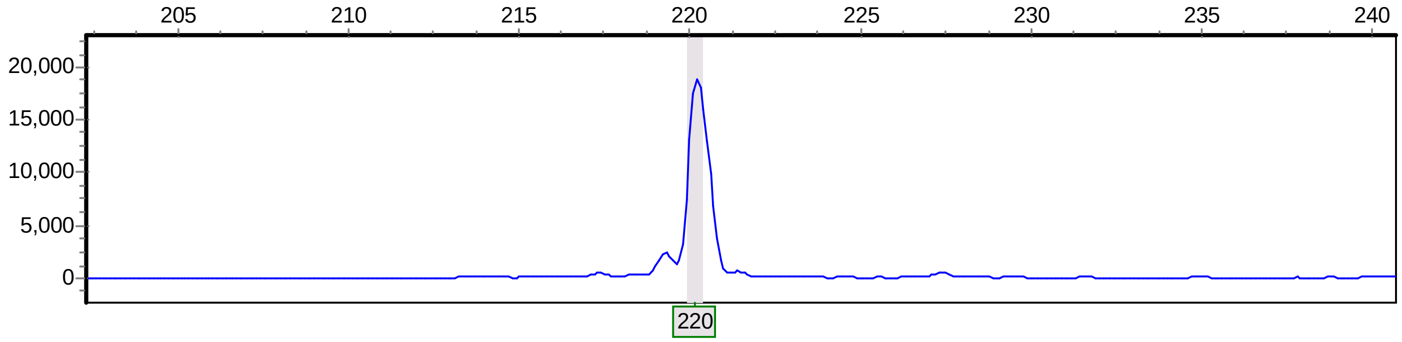

Sample 8 sorsg1-09.F01.fsa Run date and time: 03/20/2017 - 22:22:03 -> 03/20/2017 - 23:06:41

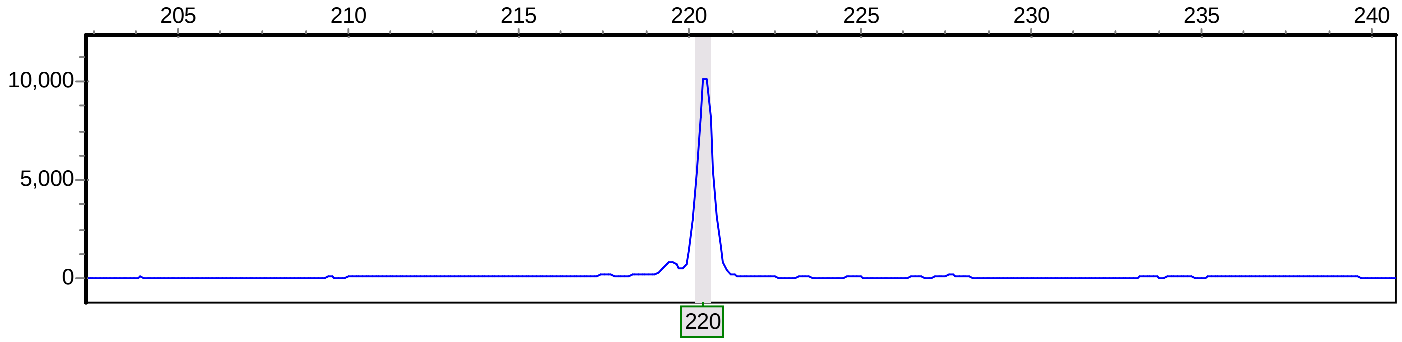

PDF 檔案使用 "pdfFactory Pro" 試用版本建立 www.pdffactory.com

Allele Report

Sample 1: sorsg8-01.A11.fsa Run date and time: 03/21/2017 - 13:32:38 -> 03/21/2017 - 14:23:07

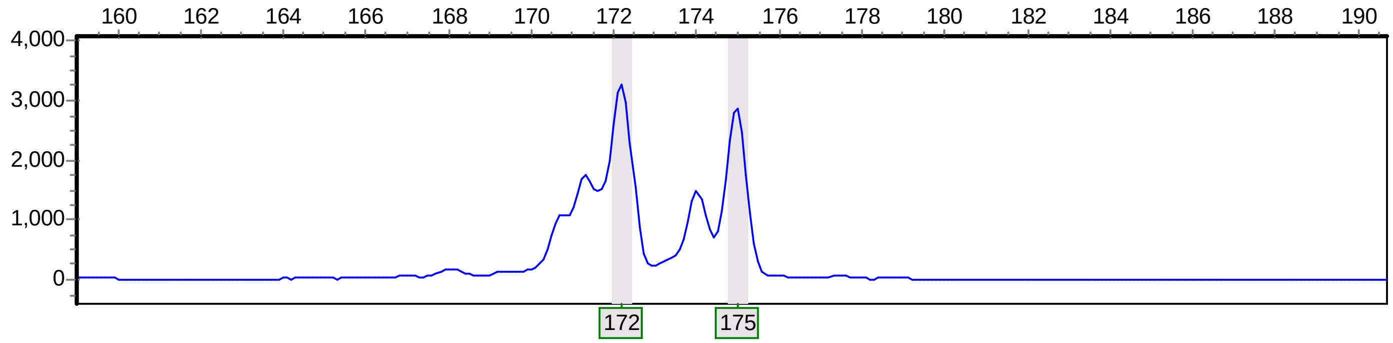

Sample 2 sorsg8-04.B11.fsa Run date and time: 03/21/2017 - 13:32:38 -> 03/21/2017 - 14:23:07

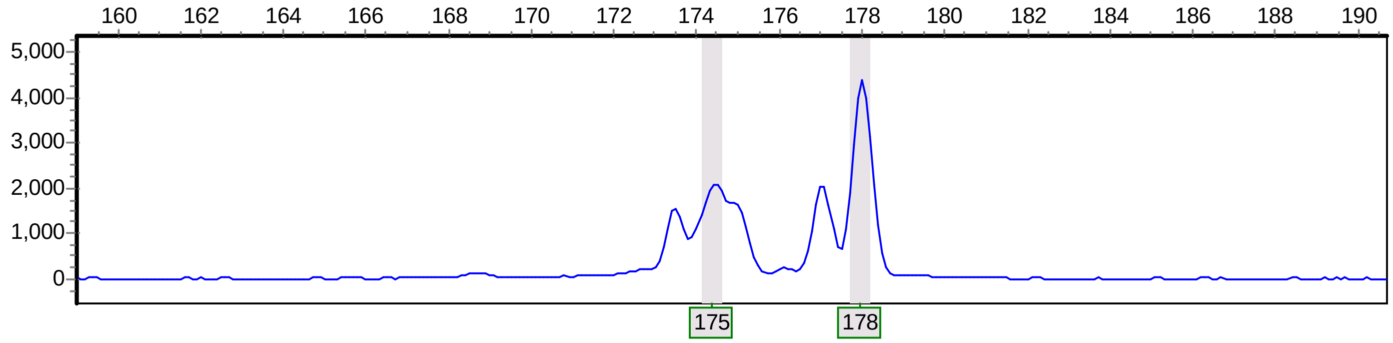

Sample 3 sorsg8-05.C11.fsa Run date and time: 03/21/2017 - 13:32:38 -> 03/21/2017 - 14:23:07

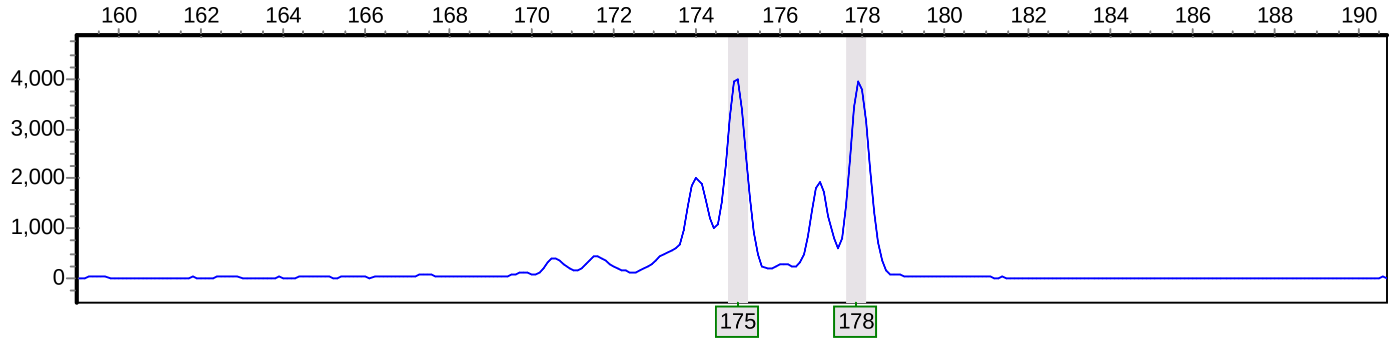

Sample 4 sorsg8-06.D11.fsa Run date and time: 03/21/2017 - 13:32:38 -> 03/21/2017 - 14:23:07

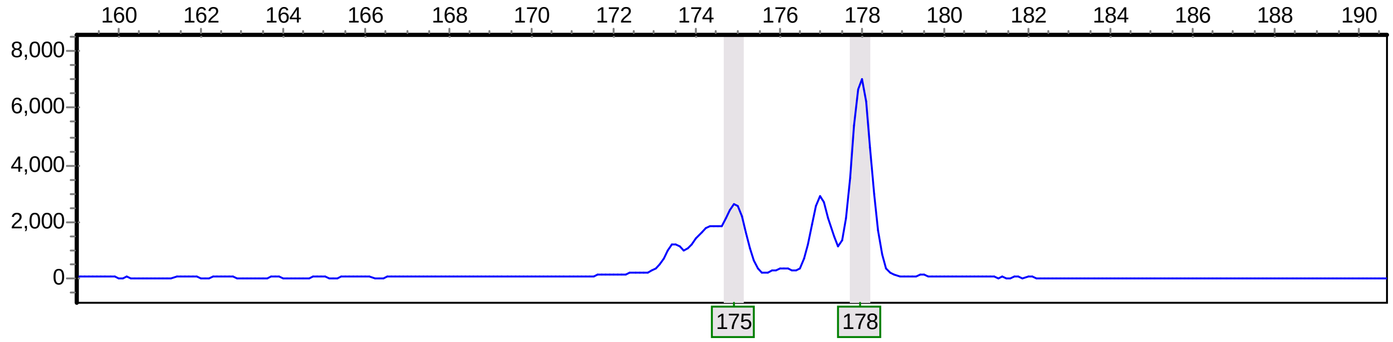

PDF 檔案使用 "pdfFactory Pro" 試用版本建立 www.pdffactory.com

Allele Report

Sample 5: sorsg8-07.E11.fsa Run date and time: 03/21/2017 - 13:32:38 -> 03/21/2017 - 14:23:07

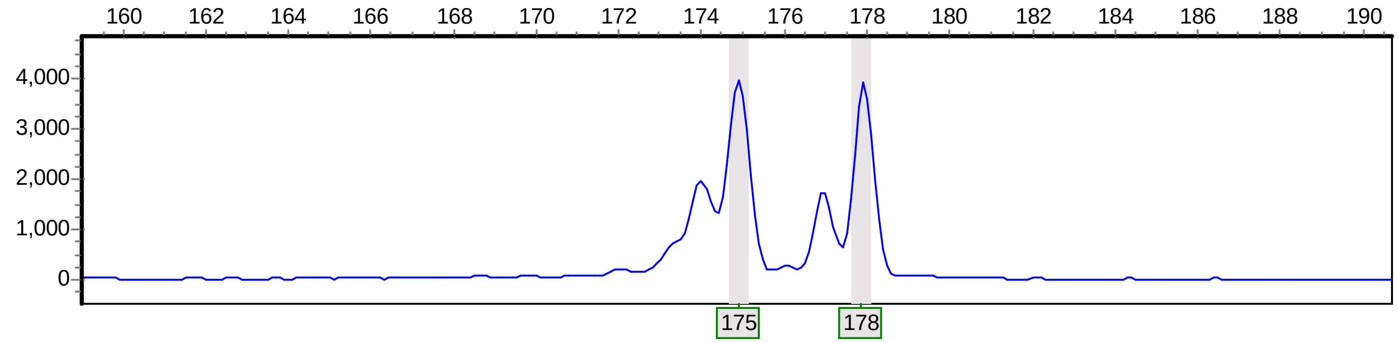

Sample 6 sorsg8-08-1.G02.fsa Run date and time: 03/19/2017 - 18:17:56 -> 03/19/2017 - 19:01:55

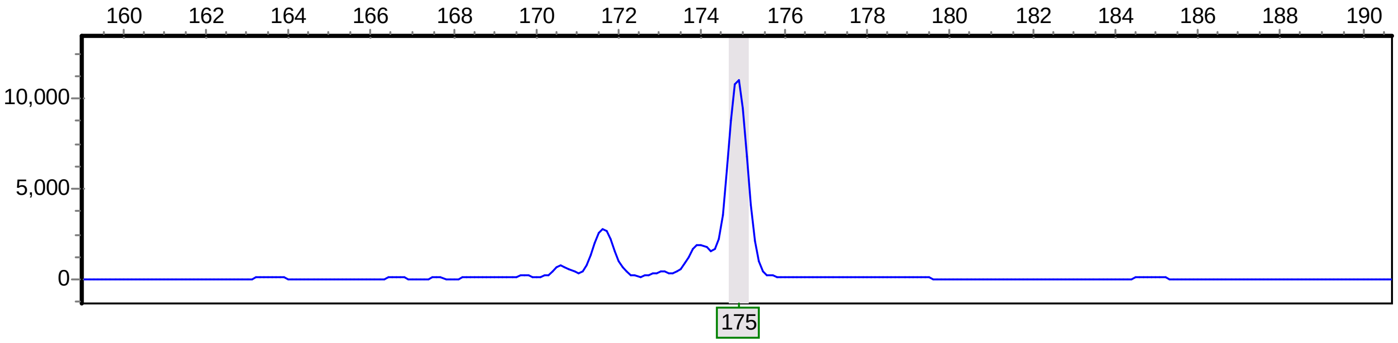

Sample 7 sorsg8-08.F11.fsa Run date and time: 03/21/2017 - 13:32:38 -> 03/21/2017 - 14:23:07

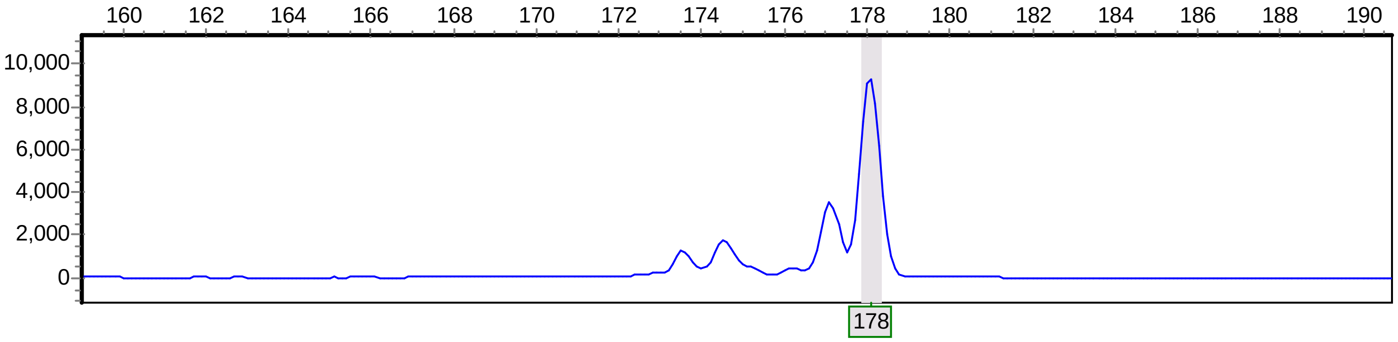

Sample 8 sorsg8-09.H02.fsa Run date and time: 03/19/2017 - 18:17:56 -> 03/19/2017 - 19:01:55

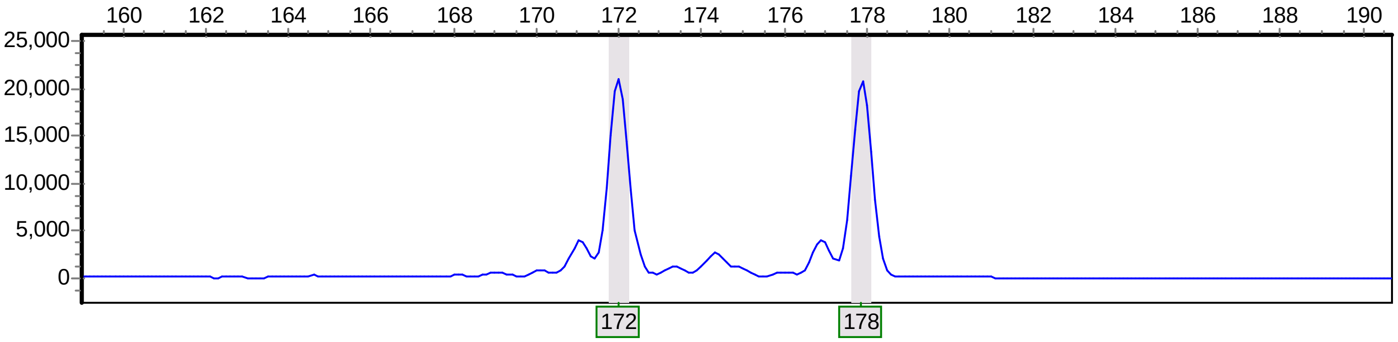

PDF 檔案使用 "pdfFactory Pro" 試用版本建立 www.pdffactory.com

SoftGenetics | # Allele Report | 2017-4-13 18:07:34

GeneMarker V2.2.0

Sample 1: sorsh13-01.A05.fsa Run date and time: 03/21/2017 - 13:32:38 -> 03/21/2017 - 14:23:07

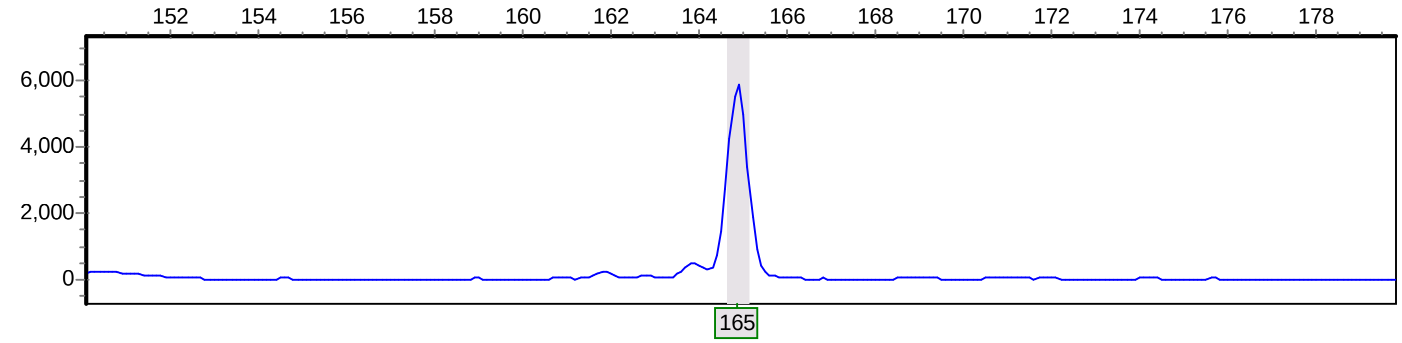

Sample 2 sorsh13-04.B05.fsa Run date and time: 03/20/2017 - 22:22:03 -> 03/20/2017 - 23:06:41

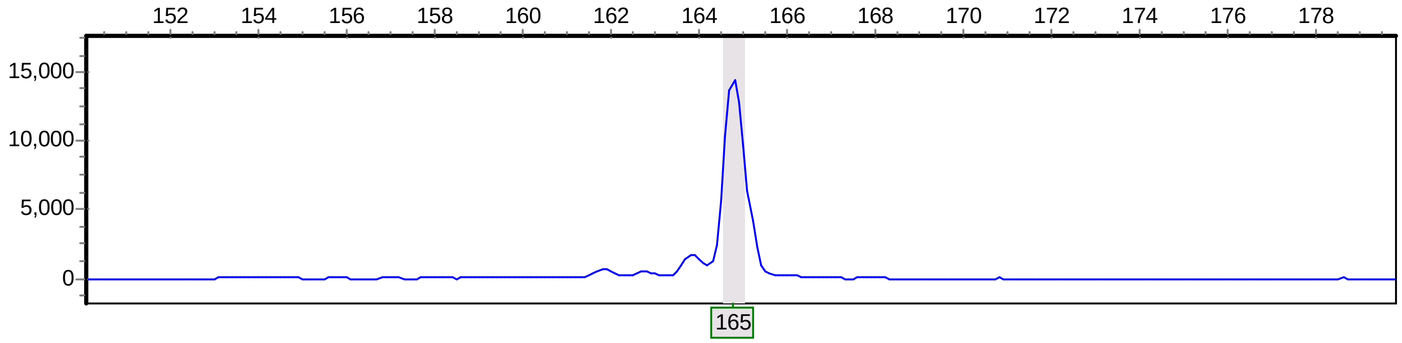

Sample 3 sorsh13-05.C05.fsa Run date and time: 03/20/2017 - 22:22:03 -> 03/20/2017 - 23:06:41

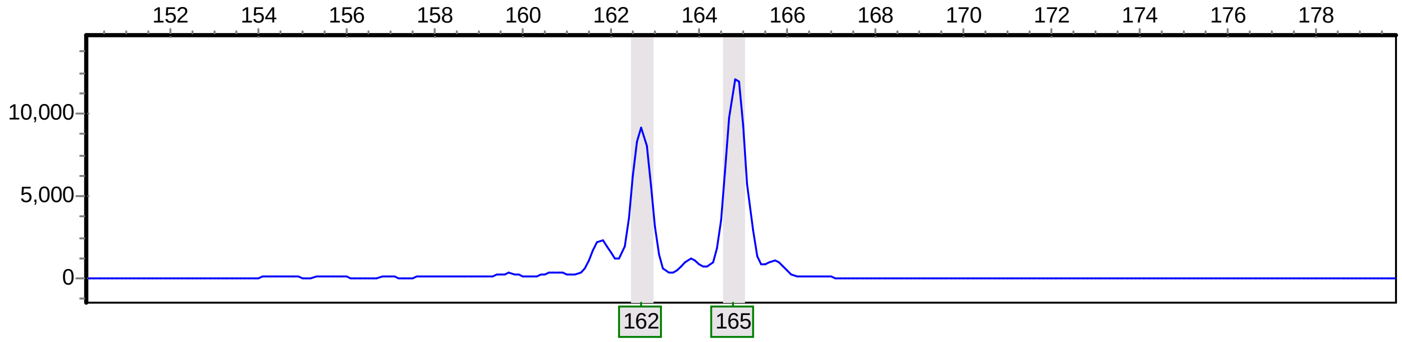

Sample 4 sorsh13-06.D05.fsa Run date and time: 03/20/2017 - 22:22:03 -> 03/20/2017 - 23:06:41

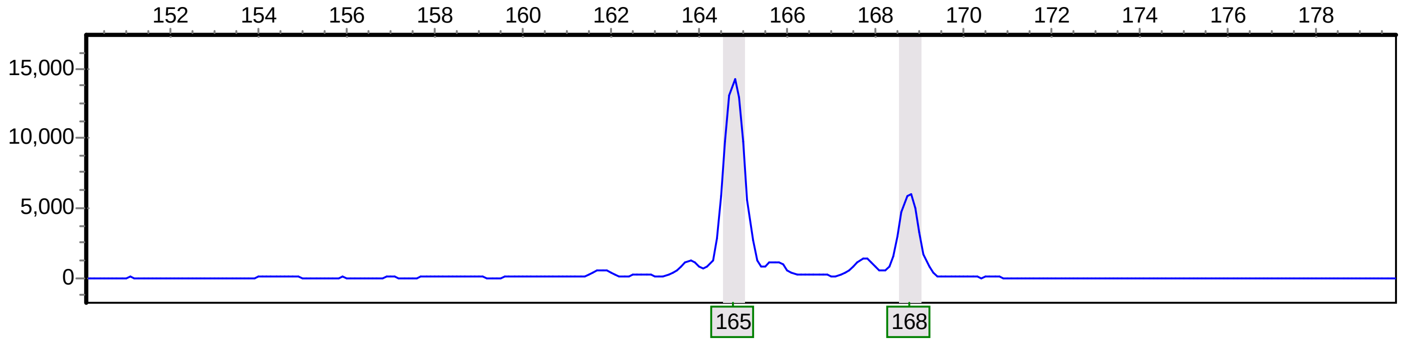

PDF 檔案使用 "pdfFactory Pro" 試用版本建立 www.pdffactory.com

Allele Report

Sample 5: sorsh13-07.E05.fsa Run date and time: 03/20/2017 - 22:22:03 -> 03/20/2017 - 23:06:41

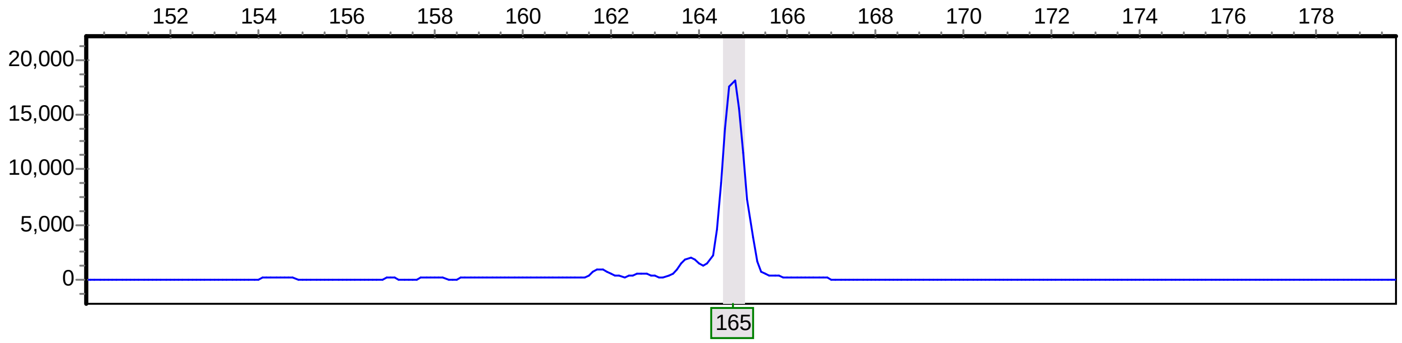

Sample 6 sorsh13-08-1.C05.fsa Run date and time: 03/19/2017 - 18:17:56 -> 03/19/2017 - 19:01:55

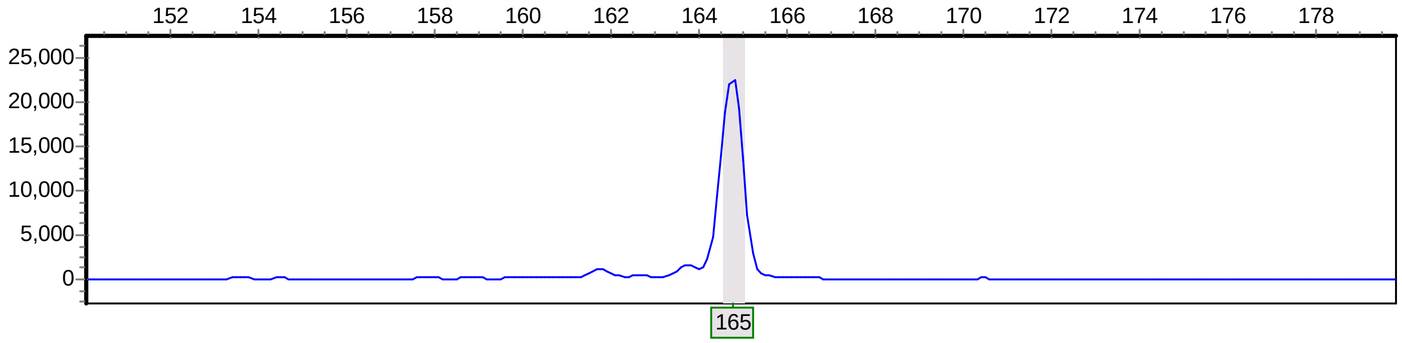

Sample 7 sorsh13-08.F05.fsa Run date and time: 03/21/2017 - 13:32:38 -> 03/21/2017 - 14:23:07

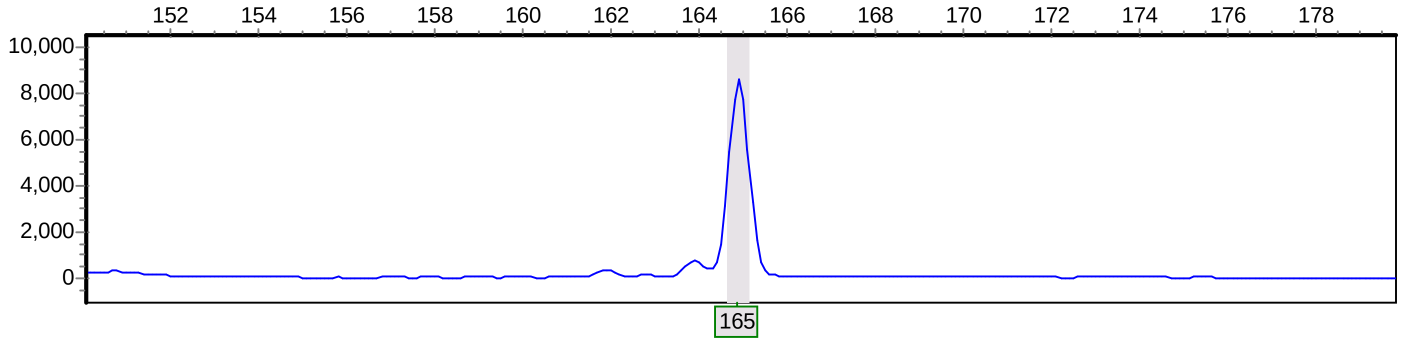

Sample 8 sorsh13-09.D05.fsa Run date and time: 03/19/2017 - 18:17:56 -> 03/19/2017 - 19:01:55

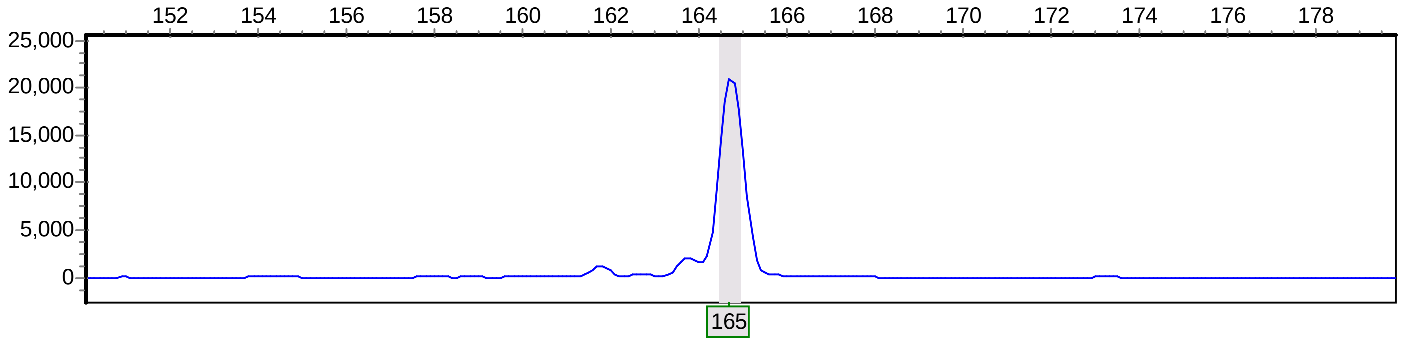

PDF 檔案使用 "pdfFactory Pro" 試用版本建立 www.pdffactory.com

Allele Report

Sample 1: sorsj14-01.A07.fsa Run date and time: 03/20/2017 - 22:22:03 -> 03/20/2017 - 23:06:41

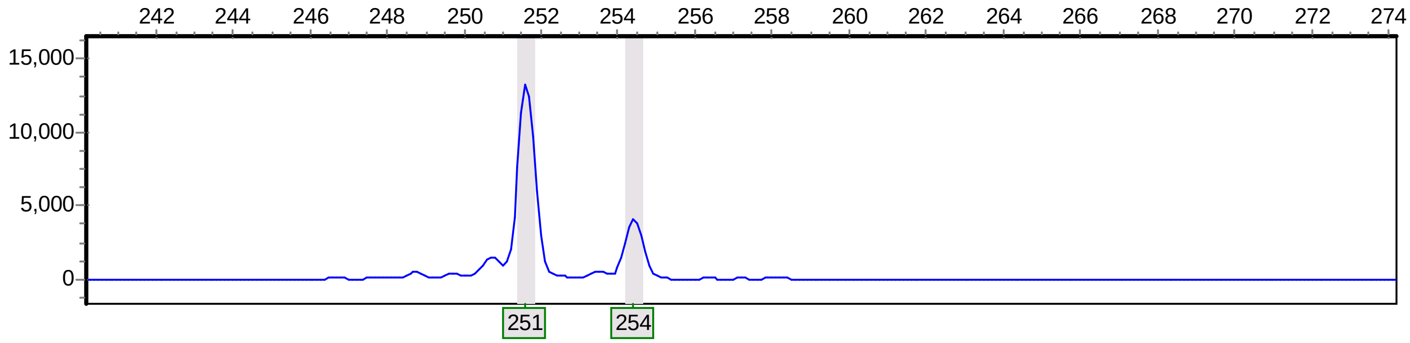

Sample 2 sorsj14-04.B07.fsa Run date and time: 03/20/2017 - 22:22:03 -> 03/20/2017 - 23:06:41

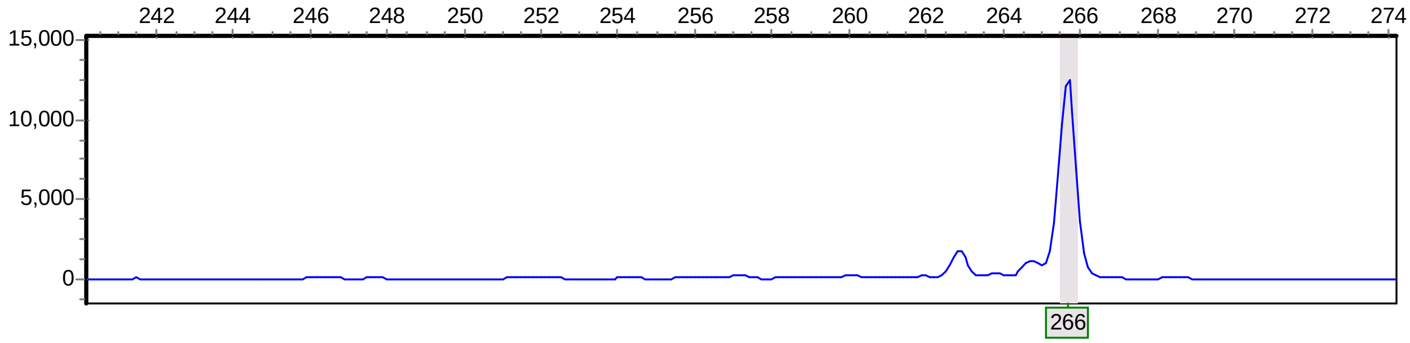

Sample 3 sorsj14-05.C07.fsa Run date and time: 03/20/2017 - 22:22:03 -> 03/20/2017 - 23:06:41

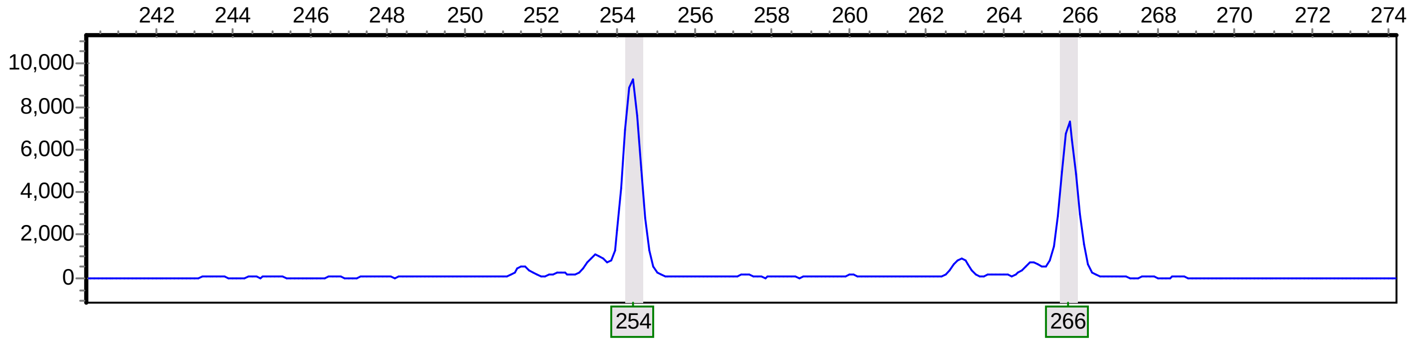

Sample 4 sorsj14-06.D07.fsa Run date and time: 03/20/2017 - 22:22:03 -> 03/20/2017 - 23:06:41

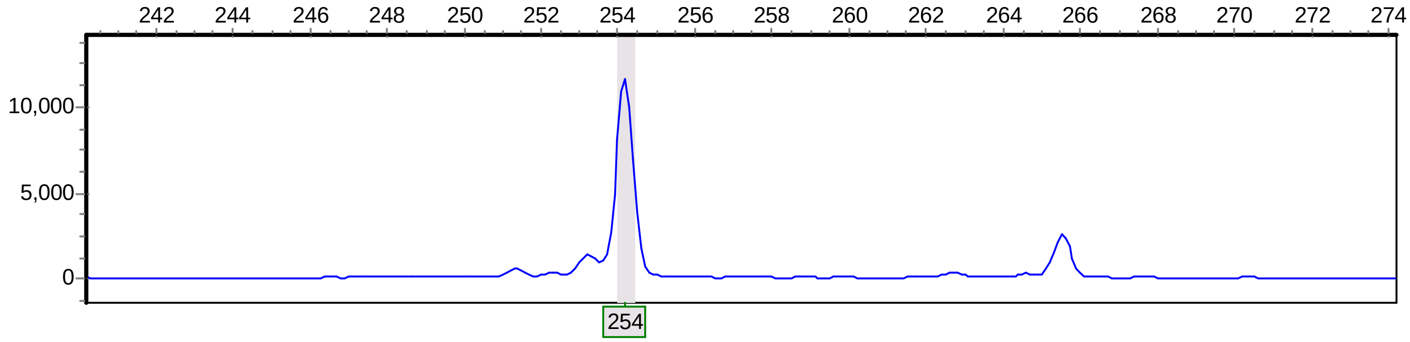

PDF 檔案使用 "pdfFactory Pro" 試用版本建立 www.pdffactory.com

Sample 5: sorsj14-07.E07.fsa Run date and time: 03/20/2017 - 22:22:03 -> 03/20/2017 - 23:06:41

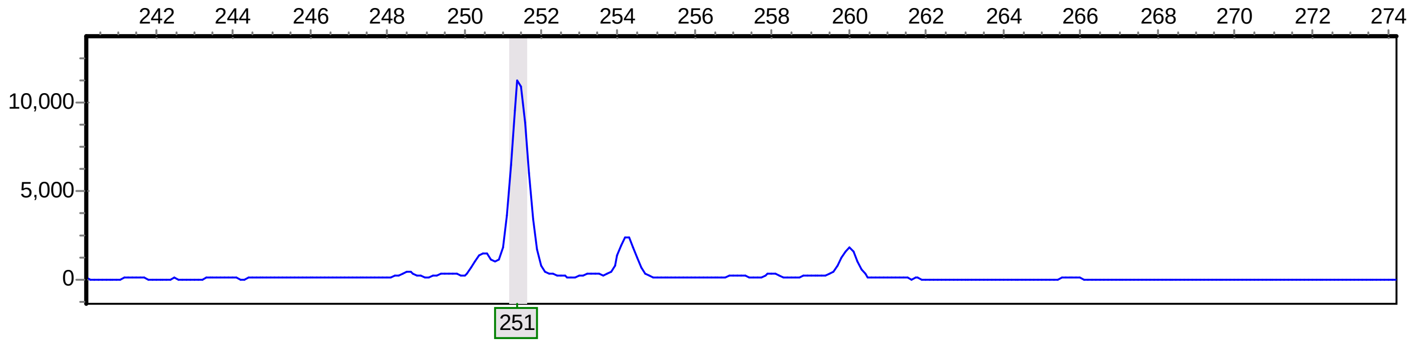

Sample 6 sorsj14-08-1.G05.fsa Run date and time: 03/19/2017 - 18:17:56 -> 03/19/2017 - 19:01:55

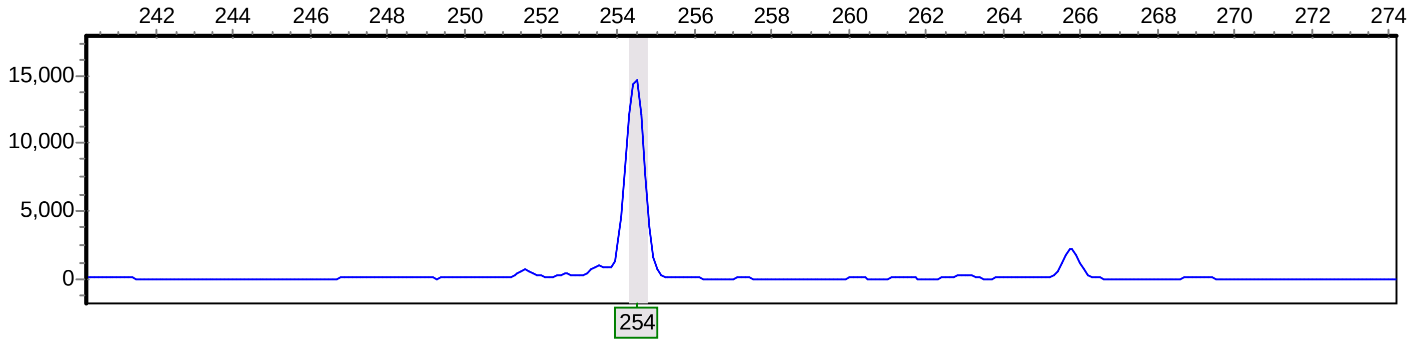

Sample 7 sorsj14-08.F07.fsa Run date and time: 03/20/2017 - 22:22:03 -> 03/20/2017 - 23:06:41

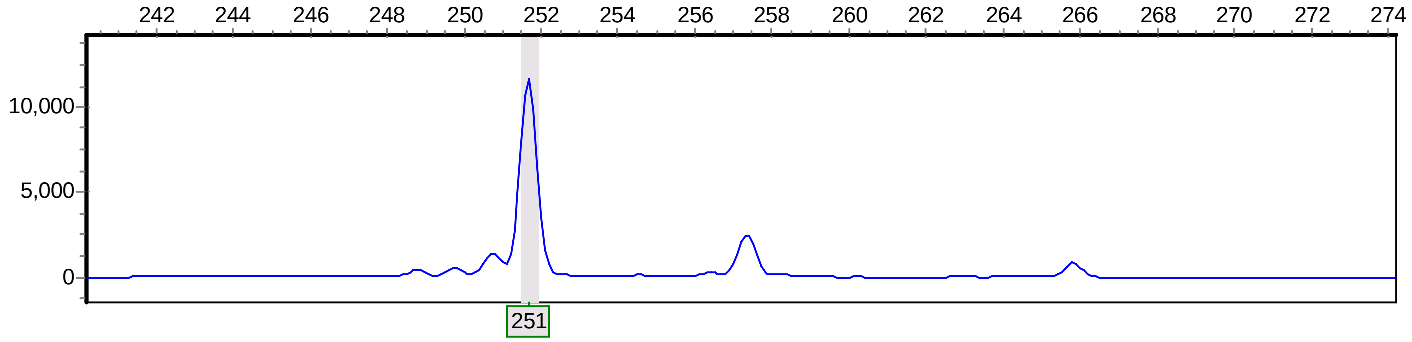

Sample 8 sorsj14-09.H05.fsa Run date and time: 03/19/2017 - 18:17:56 -> 03/19/2017 - 19:01:55

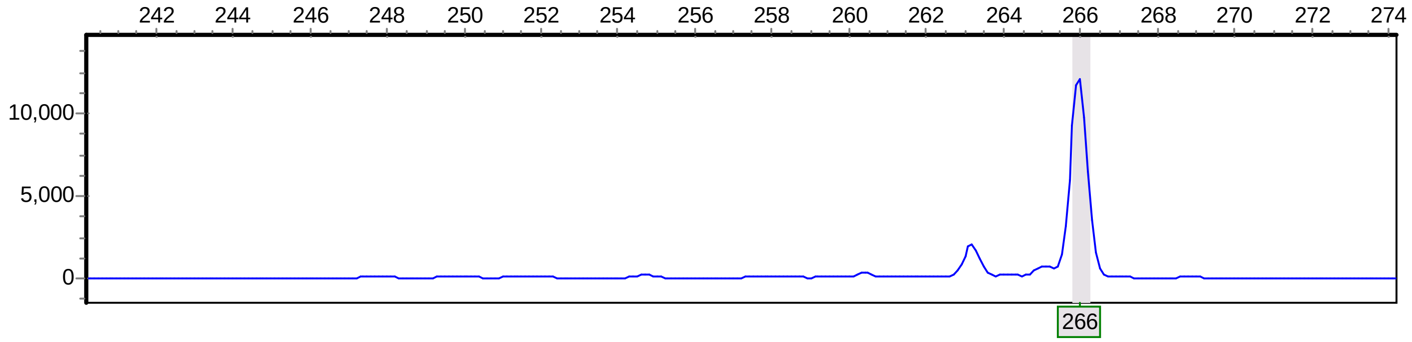

PDF 檔案使用 "pdfFactory Pro" 試用版本建立 www.pdffactory.com

Allele Report

Sample 1: sorsj16-01.A08.fsa Run date and time: 03/20/2017 - 22:22:03 -> 03/20/2017 - 23:06:41

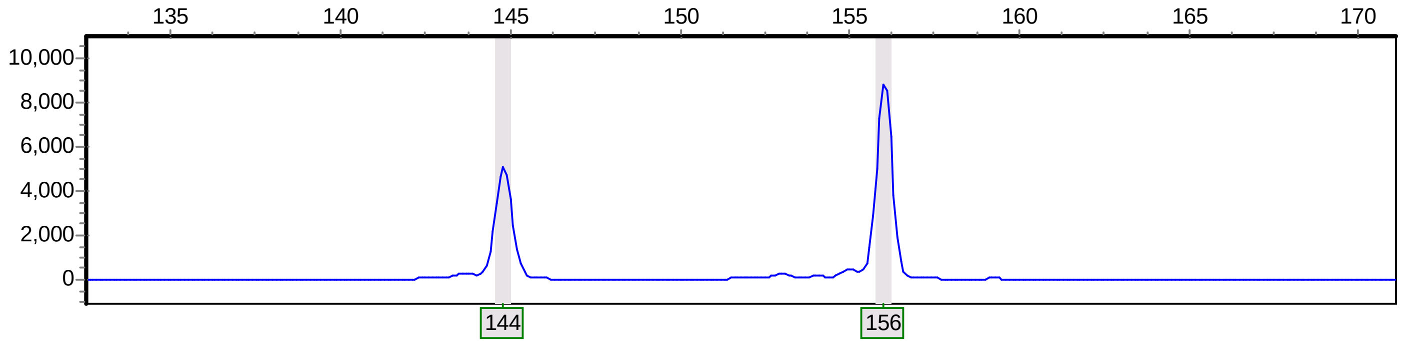

Sample 2 sorsj16-04.B08.fsa Run date and time: 03/20/2017 - 22:22:03 -> 03/20/2017 - 23:06:41

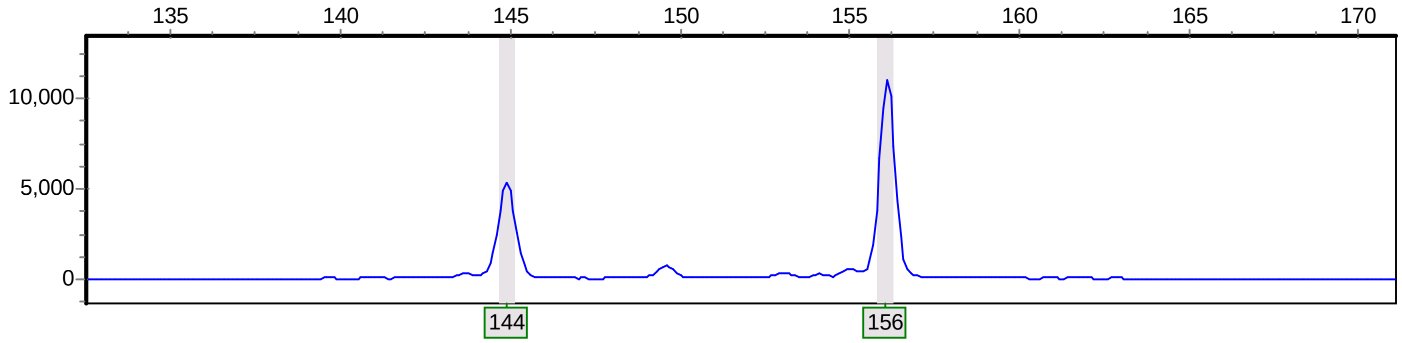

Sample 3 sorsj16-05.C08.fsa Run date and time: 03/20/2017 - 22:22:03 -> 03/20/2017 - 23:06:41

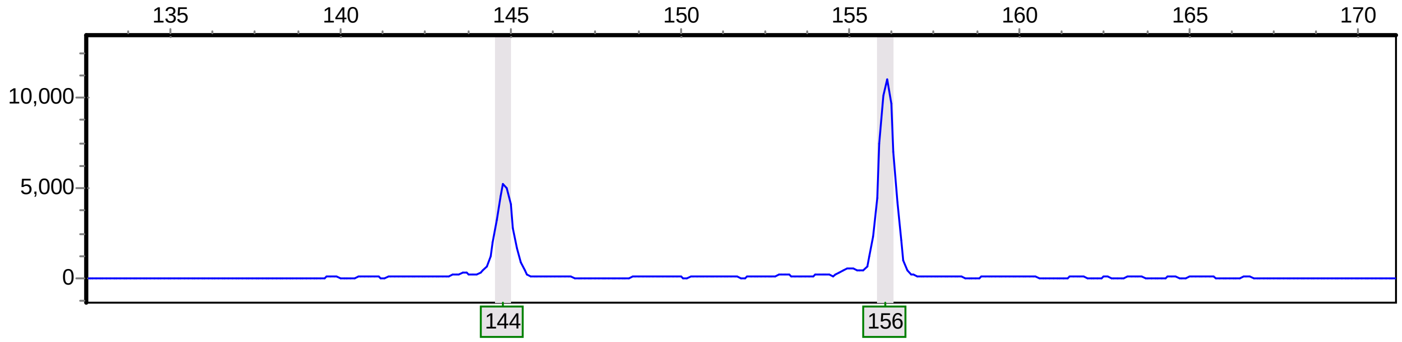

Sample 4 sorsj16-06.D08.fsa Run date and time: 03/20/2017 - 22:22:03 -> 03/20/2017 - 23:06:41

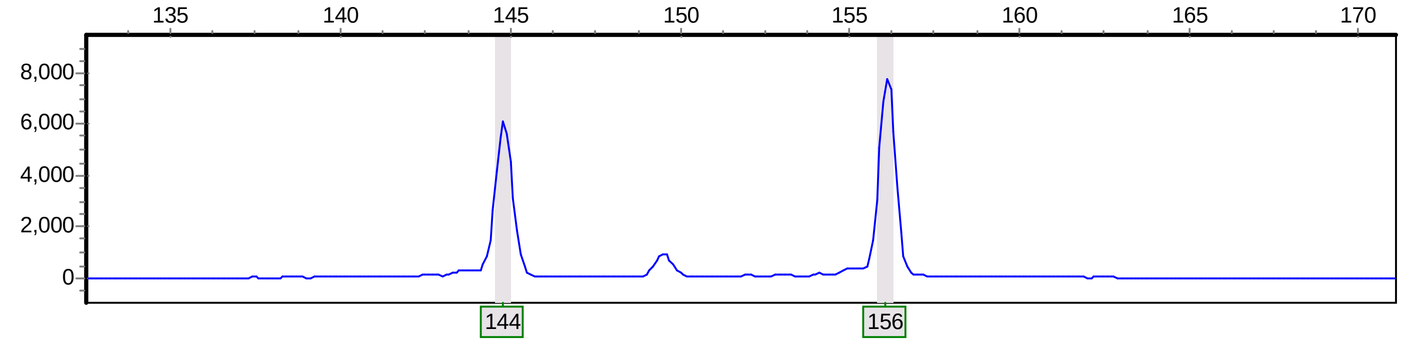

PDF 檔案使用 "pdfFactory Pro" 試用版本建立 www.pdffactory.com

Allele Report

Sample 5: sorsj16-07.E08.fsa Run date and time: 03/20/2017 - 22:22:03 -> 03/20/2017 - 23:06:41

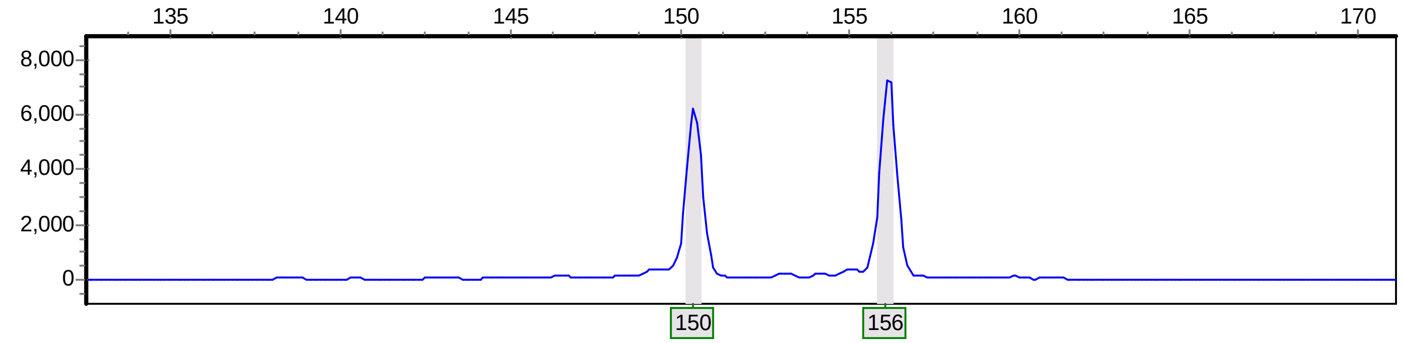

Sample 6 sorsj16-08-1.A06.fsa Run date and time: 03/19/2017 - 18:17:56 -> 03/19/2017 - 19:01:55

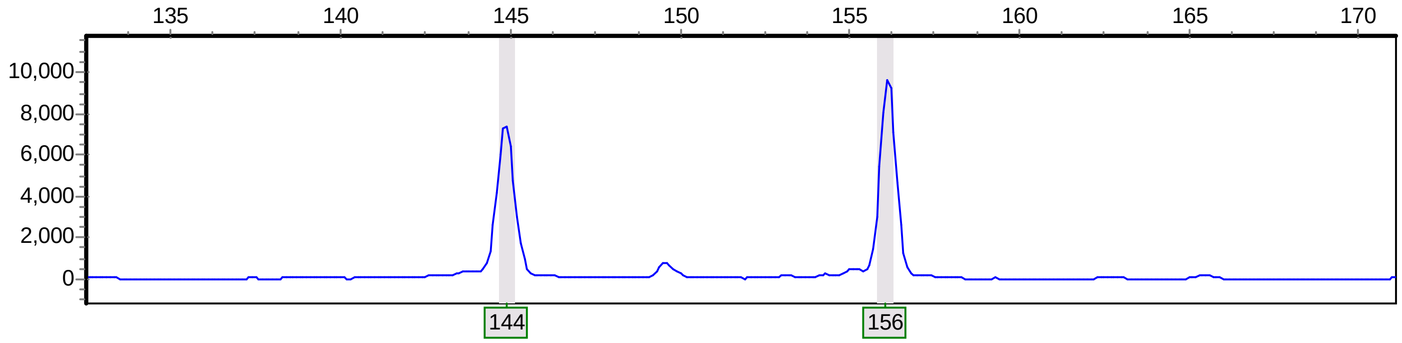

Sample 7 sorsj16-08.F08.fsa Run date and time: 03/20/2017 - 22:22:03 -> 03/20/2017 - 23:06:41

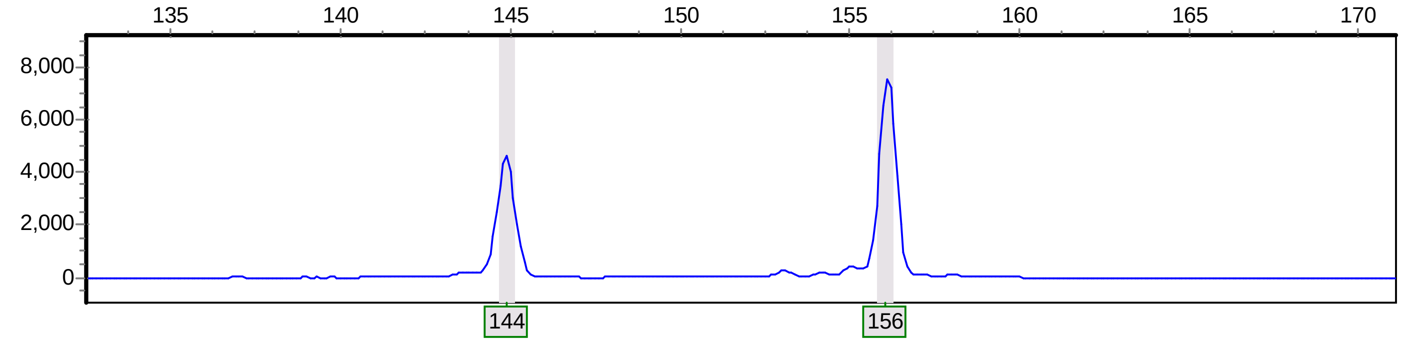

Sample 8 sorsj16-09.B06.fsa Run date and time: 03/19/2017 - 18:17:56 -> 03/19/2017 - 19:01:55

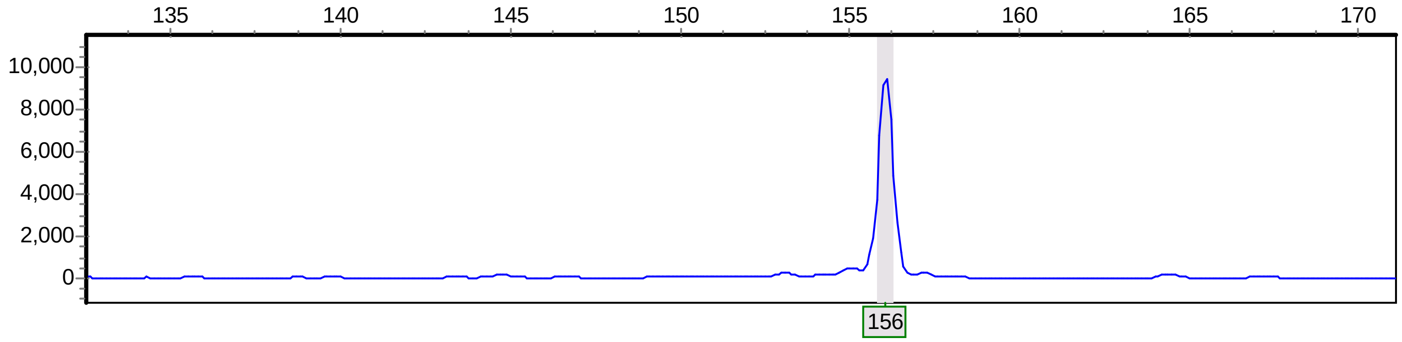

PDF 檔案使用 "pdfFactory Pro" 試用版本建立 www.pdffactory.com

Allele Report

Sample 1: sorsk10-01.A09.fsa Run date and time: 03/20/2017 - 22:22:03 -> 03/20/2017 - 23:06:41

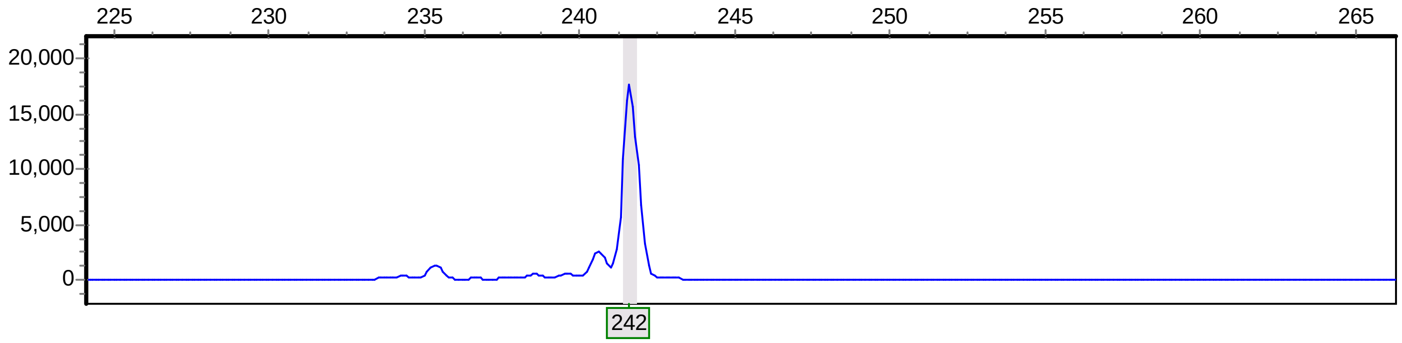

Sample 2 sorsk10-04.B09.fsa Run date and time: 03/20/2017 - 22:22:03 -> 03/20/2017 - 23:06:41

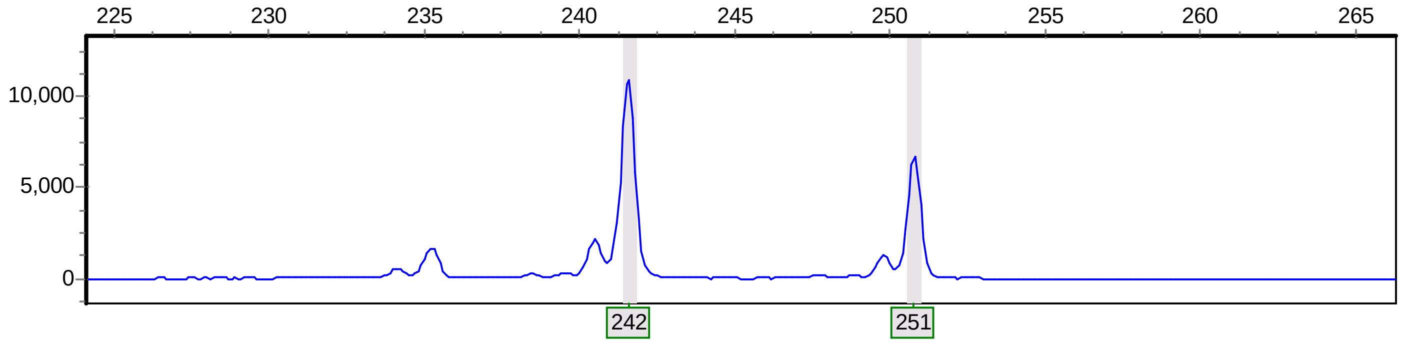

Sample 3 sorsk10-05.C09.fsa Run date and time: 03/20/2017 - 22:22:03 -> 03/20/2017 - 23:06:41

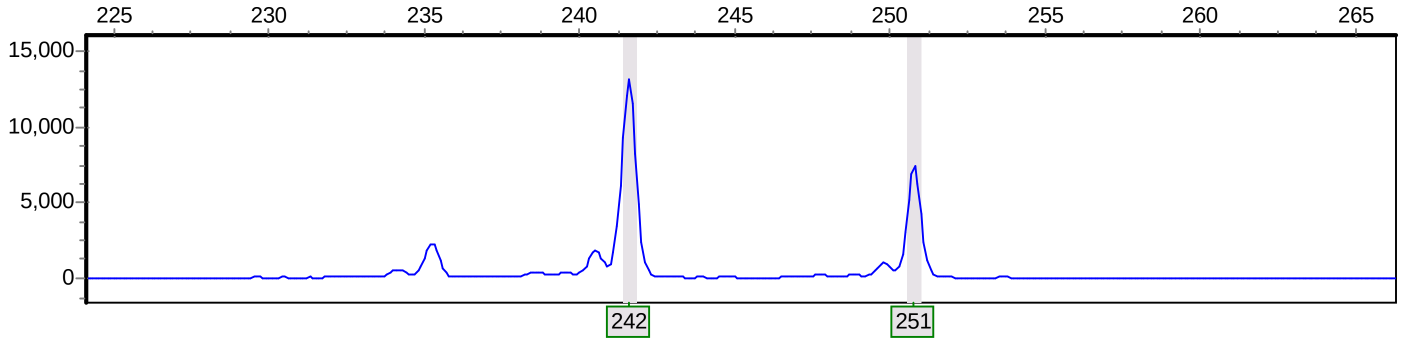

Sample 4 sorsk10-06.D09.fsa Run date and time: 03/20/2017 - 22:22:03 -> 03/20/2017 - 23:06:41

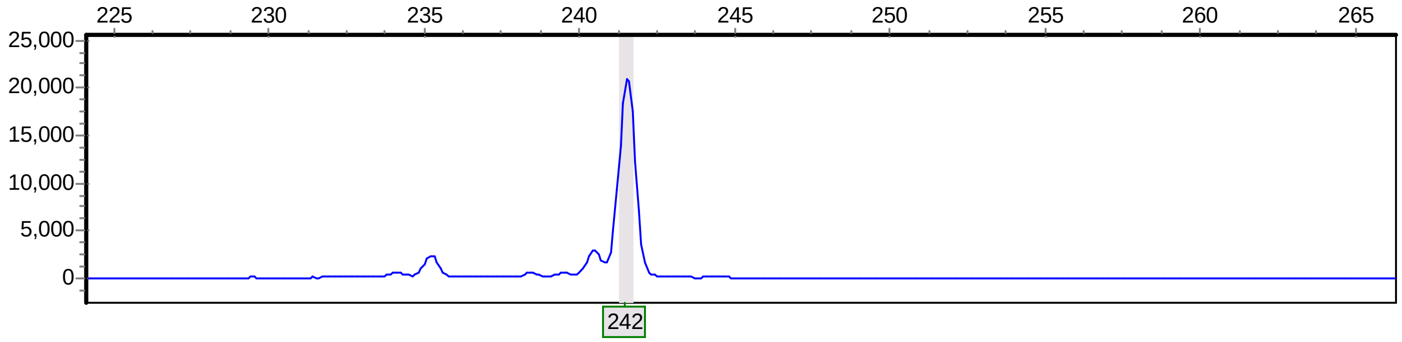

PDF 檔案使用 "pdfFactory Pro" 試用版本建立 www.pdffactory.com

Sample 5: sorsk10-07.E09.fsa Run date and time: 03/20/2017 - 22:22:03 -> 03/20/2017 - 23:06:41

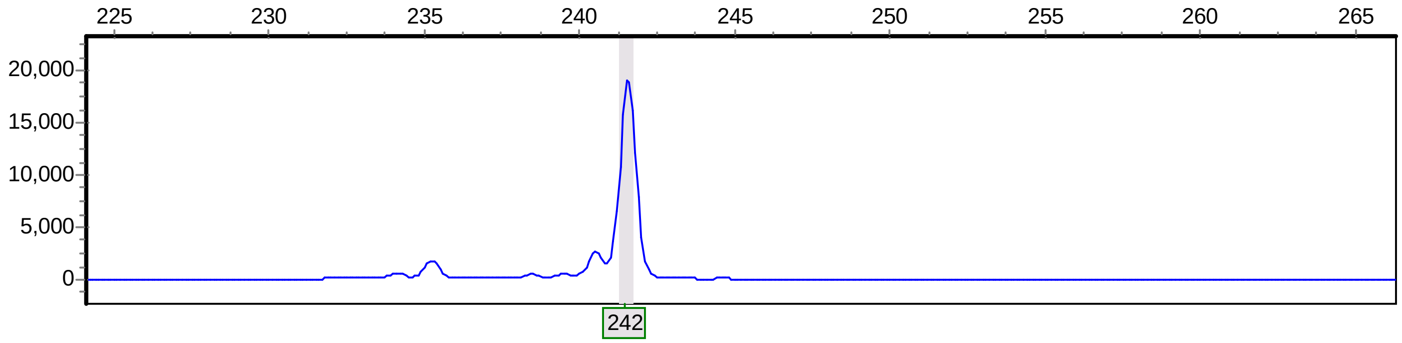

Sample 6 sorsk10-08-1.C06.fsa Run date and time: 03/19/2017 - 18:17:56 -> 03/19/2017 - 19:01:55

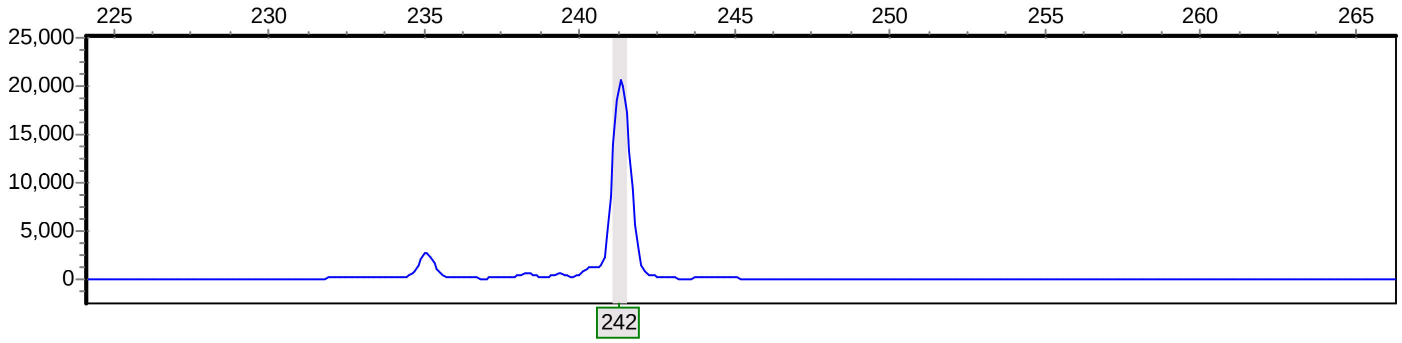

Sample 7 sorsk10-08.F09.fsa Run date and time: 03/20/2017 - 22:22:03 -> 03/20/2017 - 23:06:41

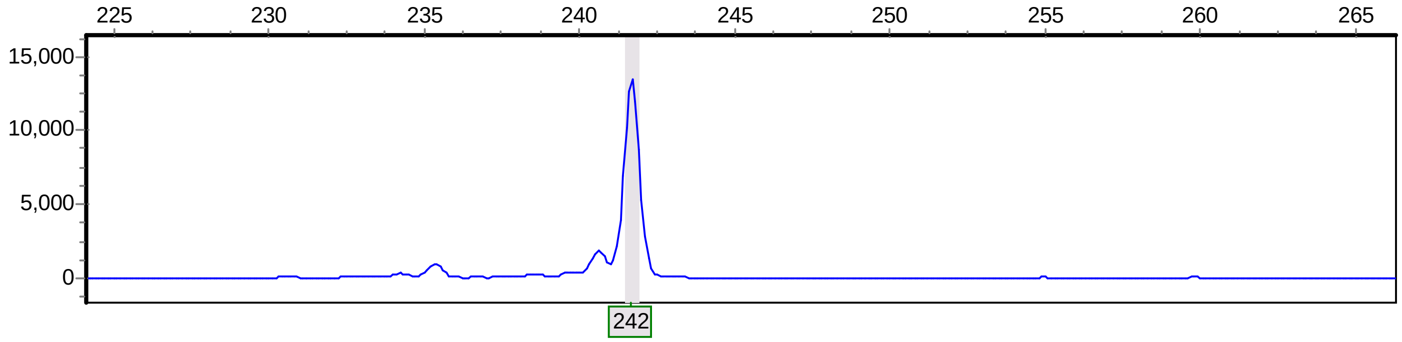

Sample 8 sorsk10-09.D06.fsa Run date and time: 03/19/2017 - 18:17:56 -> 03/19/2017 - 19:01:55

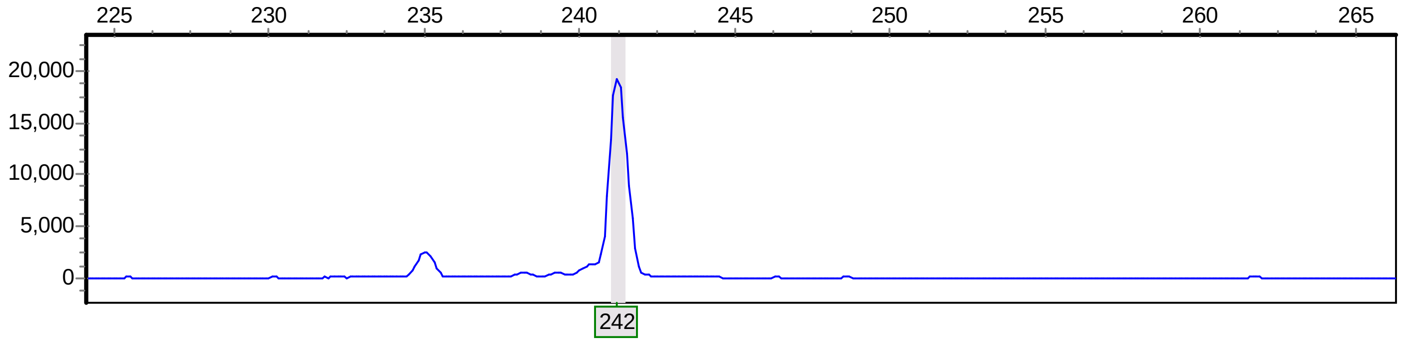

PDF 檔案使用 "pdfFactory Pro" 試用版本建立 www.pdffactory.com
